# Immune Dysregulation as a Driver of Coronary Heart Disease

**DOI:** 10.3390/ijms27209149

**Published:** 2026-10-15

**Authors:** Sijie Chu, Huifen Zhou, Haitong Wan

**Affiliations:** 1 School of Basic Medical Sciences, Zhejiang Chinese Medical University, Hangzhou 310053, China; 202211010211006@zcmu.edu.cn (S.C.); 20121021@zcmu.edu.cn (H.Z.); 2 Academy of Chinese Medical Sciences, Henan University of Chinese Medicine, Zhengzhou 450046, China

**Keywords:** coronary heart disease, immune cells, atherosclerosis, inflammatory cytokines

## Abstract

Coronary heart disease (CHD) affects millions of individuals worldwide and remains a leading cause of morbidity and mortality. It compromises quality of life primarily through myocardial ischemia resulting from progressive coronary arterial narrowing and contributes to a wide range of adverse clinical outcomes. Although current therapeutic strategies, including coronary revascularization, lipid-lowering therapy, and plaque stabilization, have substantially improved patient prognosis, they do not fully address the underlying immunopathological mechanisms driving disease progression. Increasing evidence suggests that immune-mediated inflammation and vascular injury play critical roles in the initiation, progression, and destabilization of atherosclerotic plaques, highlighting the immune system as a potential therapeutic target in CHD management. In this review, we summarize recent advances in our understanding of the contributions of innate and adaptive immune responses to CHD pathogenesis. Collectively, current evidence indicates that immune dysregulation is a central driver of atherogenesis and its clinical manifestations, supporting the development of targeted immunomodulatory strategies as promising approaches for the prevention and treatment of CHD.

## 1. Introduction

Coronary heart disease (CHD) is a leading cause of death worldwide. According to the Global Burden of Disease study, ischemic heart disease remains the leading cause of years of healthy life lost, accounting for approximately 175 million disability-adjusted life-years globally per annum. The World Health Organization projects that cardiovascular diseases will claim an estimated 23.6 million lives annually by 2030 [1,2]. Consequently, elucidating the pathogenesis of CHD is of paramount importance, as it provides a foundation for the development of preventive strategies, diagnostic approaches, and the development of targeted therapeutics [3].

The principal pathological basis of CHD is atherosclerosis (AS). This process is initiated by the accumulation and oxidative modification of lipoproteins within the arterial wall, triggering a complex pathological cascade involving cholesterol deposition, inflammatory cell recruitment, and atherosclerotic plaque formation. These events progressively lead to arterial luminal narrowing or occlusion, culminating in myocardial ischemia and potentially fatal cardiovascular events, including acute coronary syndromes [4]. The pathogenesis of AS is driven by four interconnected pathological processes: endothelial dysfunction, lipid accumulation, inflammatory infiltration, and thrombosis [5]. Importantly, maladaptive immune responses significantly influence AS progression and the incidence of cardiovascular events; thus, modulating immune cell function and associated cytokine networks represents a promising strategy to limiting AS progression [6,7]. Accordingly, current clinical management of CHD aims not only to restore coronary perfusion, lower cholesterol levels, and stabilize plaques, but also to mitigate inflammation and immune-mediated vascular injury [7,8,9].

### 1.1. Historical Perspective: From Lipid Hypothesis to Immune-Driven Atherosclerosis

The concept that immune pathways participate in atherogenesis was first proposed in 1959, when several investigators suggested that both innate and adaptive immune mechanisms participate in this process [10]. This paradigm was substantially advanced in 1992 by the “atherosclerotic autoimmunity” hypothesis. This theory posits that the early stages of AS are characterized by an inflammatory response triggered by heat shock protein 60 (HSP60); which promotes the infiltration of activated macrophages into the arterial intima and drives subsequent pathogenic events, including foam cell formation [11].

### 1.2. Innate Immunity in Atherosclerosis

Both innate and adaptive immune mechanisms orchestrate the inflammatory processes underlying AS development [12]. The innate immune response in atherosclerosis is mediated predominantly by monocytes and macrophages, although other innate immune cells—including neutrophils, dendritic cells, mast cells, and natural killer cells—also contribute significantly and respond to excessive lipoprotein accumulation. Modified low-density lipoprotein (LDL) particles activate circulating monocytes, promoting their recruitment to the vessel wall and driving intimal macrophage accumulation [13,14].

### 1.3. Adaptive Immunity in Atherosclerosis

Conversely, adaptive immunity in AS is mediated largely by T-cell subsets that secrete various cytokines and by B cells that produce antibodies, both of which exert complex pro-inflammatory or anti-inflammatory effects [15,16]. T-cell-derived chemokines amplify local immune signaling by recruiting additional immune cells to the arterial intima. Furthermore, T cells secrete growth factors and cytokines that stimulate the migration and proliferation of vascular smooth muscle cells, actively contributing to plaque progression and remodeling [17].

### 1.4. Trained Immunity: A Bridge Between Innate Memory and Chronic Inflammation

Distinct from classical innate and adaptive immunity, “trained immunity” refers to the capacity of the innate immune system to acquire a memory-like state following exposure to exogenous or endogenous stimuli. This adaptation results in an amplified response upon secondary stimulation [18,19]. In the context of atherosclerosis, monocytes and macrophages stimulated by oxidized LDL (ox-LDL) undergo extensive metabolic and epigenetic reprogramming. Such reprogramming leads to sustained hyperactivation of the innate immune system and maintenance of a pro-inflammatory phenotype [20].

### 1.5. Scope and Rationale of This Review

Basic and clinical research demonstrates that maladaptive immune responses persist throughout the course of CHD, actively promoting the initiation and progression of atherosclerosis [21]. Furthermore, a prospective cohort study showed that elevated granulocyte counts are associated with an increased risk of atherosclerotic cardiovascular disease in the general population. Notably, these elevated counts positively correlate with coronary calcification volume, underscoring the close relationship between immune-mediated inflammation and AS development [22].

#### 1.5.1. Experimental Animal Models of CHD: Stage-Specific Applicability and Limitations

In this review, we synthesize evidence from both clinical and preclinical studies. We primarily focus on studies published in recent years (summarized in Table 1), while appropriately referencing foundational literature to provide essential historical context. A diverse array of animal models has been employed to investigate the immunological mechanisms of CHD. Critically, different models recapitulate different pathological stages of the disease (see Section 2.1 for the three-stage framework), and no single model faithfully reproduces the entire spectrum from chronic atherogenesis through plaque rupture to post-infarction repair. Understanding the stage-specific applicability of each model is essential for correctly interpreting the immunological findings discussed in this review.

Stage I models (chronic atherogenesis): Apolipoprotein E-deficient (ApoE^−/−^) and low-density lipoprotein (LDL) receptor-deficient (Ldlr^−/−^) mice, maintained on high-fat or Western-type diets, are the most widely used models for studying the immunology of atherosclerotic plaque initiation and progression. These models develop lipid-rich plaques with macrophage infiltration, T-cell accumulation, and fibrous cap formation, which closely parallel human atherogenesis. These studies have been instrumental in establishing the roles of monocyte/macrophage subsets, T helper cell polarization, regulatory T cells (Tregs), B-cell antibody responses, and dendritic cell antigen presentation in plaque biology. However, these models have a critical limitation: they do not spontaneously develop plaque rupture with superimposed luminal thrombosis (Stage II), nor do they experience myocardial infarction (Stage III). Findings from ApoE^−/−^ and Ldlr^−/−^ models should therefore be interpreted strictly within the context of chronic atherogenesis (Stage I).

Stage II models (plaque destabilization and rupture): This remains the most challenging stage to model experimentally. Emerging approaches include the tandem stenosis model (which induces hemodynamic stress-driven plaque instability in ApoE^−/−^ mice) and the ApoE^−/−^Fbn1C1039G^+/−^ compound mutant mouse (which develops spontaneous plaque rupture due to defective fibrillin-1-mediated vascular integrity). Although these models show promise, their immunological characterization is still in the early stages, and the majority of immunological evidence for Stage II is currently derived from human clinical studies (thrombus aspirates, culprit lesion histology, and intracoronary imaging).

Stage III models (post-infarction repair and remodeling): Left anterior descending (LAD) coronary artery ligation [23,24]—either permanent or with transient ischemia followed by reperfusion—is the most widely used model for studying the immunology of myocardial infarction and subsequent wound healing. This model produces a focal ischemic infarct with a well-defined border zone, and elicits the characteristic biphasic immune response that governs post-MI remodeling. Critically, LAD ligation bypasses the atherosclerotic process entirely: no plaque formation, plaque rupture, or thrombosis occurs. Coronary occlusion is surgically imposed, rather than arising from endogenous vascular disease. Therefore, findings from this model should be interpreted as reflecting the immunology of myocardial wound healing (Stage III) and not the immunology of plaque biology (Stage I) or rupture (Stage II).

Isoproterenol-induced myocardial injury [25,26] represents an even more reductionist Stage III model. Isoproterenol, a synthetic catecholamine, induces diffuse subendocardial necrosis through β-adrenergic receptor-mediated oxidative stress and intracellular calcium overload, without any vascular pathology—specifically, it involves no atherosclerosis, coronary occlusion, or focal ischemia. This model is useful for studying generic myocardial injury and repair responses (e.g., inflammatory cell infiltration kinetics, fibroblast activation, and scar formation). However, its findings should not be extrapolated to the immunology of plaque-driven CHD without considerable caution.

These models successfully recapitulate key pathological features within their respective stages, including characteristic alterations in electrocardiography, echocardiography, and circulating cardiac biomarkers. Although no single animal model can fully replicate the pathophysiological complexity and multidecade timeline of human CHD, the complementary use of Stage I, II, and III models provides crucial mechanistic insights that are difficult to ascertain from clinical studies alone. Throughout this review, each cited animal study is annotated with its corresponding disease stage to facilitate accurate interpretation.

#### 1.5.2. Organizational Framework of This Review: A Three-Level Immunopathological Model

Given that the molecular pathways discussed in this review span a broad range—from innate immune mechanisms, such as NLRP3 inflammasome activation and GSNOR-mediated Akt S-nitrosylation, to adaptive immune events, including T cell repertoire alterations and memory B-cell class-switch recombination—a unified thematic framework is needed to integrate these seemingly disparate molecular discoveries into a coherent immunopathological narrative. To this end, this review adopts a three-level immunopathological model to organize its content:

Level 1: Innate immune initiation (sterile inflammation). Metabolic danger signals (e.g., oxidized LDL [Ox-LDL] accumulation and hyperhomocysteinemia) are first sensed by the innate immune system. NLRP3 inflammasome activation represents the classical innate inflammatory pathway: NLRP3 assembly in macrophages and endothelial cells leads to caspase-1 activation, promoting the maturation and release of IL-1β and IL-18 and establishing a persistent pro-inflammatory signaling loop. Concurrently, GSNOR-mediated Akt S-nitrosylation represents a non-classical innate pathway, and aberrant GSNOR activity in endothelial cells disrupts nitric oxide/redox signaling, compromising vascular homeostasis and enhancing leukocyte recruitment. These two pathways ultimately converge on NF-κB-dependent cytokine production, jointly establishing an inflammatory microenvironment that primes adaptive immune activation.

Level 2: Amplification and specialization of adaptive immunity. The inflammatory microenvironment established by the innate immune system activates and polarizes adaptive immune cells. Within this framework, T-cell repertoire alterations (including γδT-cell activation) and memory B-cell class-switch recombination are not isolated events but rather downstream consequences of sustained innate immune signaling. Specifically, dendritic cells activated by damage-associated molecular patterns (DAMPs) and NLRP3-derived IL-1β/IL-18 present modified self-antigens (e.g., ApoB peptide fragments and oxidation-specific epitopes [OSEs]) to T cells, driving Th1/Th17 polarization, Treg dysfunction, and antigen-specific clonal expansion. Simultaneously, T-cell-derived cytokines (e.g., IL-21 and IFN-γ) and innate cell-derived factors (e.g., BAFF/APRIL) synergistically promote B-cell activation, germinal center reactions, and isotype switching—from protective IgM to potentially pathogenic IgG subclasses.

Level 3—Tissue microenvironment integration. The immune responses of the preceding two levels ultimately converge within specific tissue microenvironments, particularly epicardial adipose tissue (EAT) and atherosclerotic plaques. In these niches, immune cells, lipids, and stromal cells interact in a spatially organized manner to determine disease trajectory. This level is elaborated on in the discussion section (Section 4.3).

This three-level framework serves as the organizational backbone of the review. Section 2 (innate immunity) corresponds to Level 1, Section 3 (adaptive immunity) corresponds to Level 2, and Section 4 (discussion) focuses on tissue microenvironment integration and cross-level signaling networks. In the subsequent sections, each finding is cross-referenced to its corresponding level to facilitate interpretation within this unified immunopathological framework.

**Table 1:** Regulation of various immune-related monitoring factors in immune cells during coronary heart disease.

Immune Cell Type	Disease Model/Cohort/ Experimental System	Immune-Related Molecules or Findings	Direction of Change	Species/ System	Species/ System	Ref.
Monocytes	CHD patients and healthy subjects	MDMTG2, CD206, CD163, TF	CD206 ↑, CD163 ↑ (associated with plaque morphology); TF ↑	Human	Stage I	[27]
	CHD patients and healthy subjects	Monocyte chemoattractant protein-1 (MCP-1)	MCP-1 ↑ (epigenetically upregulated in CD14^+^ monocytes)	Human	Stage I	[28]
	CHD patients	Classical (CD14^++^CD16^−^), non-classical (CD14^+^CD16^++^), intermediate (CD14^++^CD16^+^) monocyte subsets	Intermediate monocytes ↑, associated with 10-year CVD risk	Human	Stage I	[29]
	AMI patients, unstable angina patients, and healthy subjects	MHR	MHR ↑, significantly elevated in AMI and UA patients	Human	Stage II	[30]
	NHANES cohort	Monocyte-to-HDL ratio (MHR)	MHR ↑, positively correlated with CHD prevalence risk	Human	Cross-stage	[31]
	CHD patients	MHR	MHR ↑ predicts long-term adverse outcomes	Human	Cross-stage	[32,33]
	CHD patients	Inflammatory CD14^+^CD16^+^ monocytes increased; IL-15 and fractalkine elevated	CD14^+^CD16^+^ monocytes ↑; IL-15 ↑; fractalkine ↑	Human	Cross-stage	[34]
Macrophages	CHD patients	Higher monocyte infiltration; lower M0/M2 macrophages in high CHD score group; candidate markers: TLR3, MLKL, HMGB1, NDRG2	Monocyte infiltration ↑; M0 ↓, M2 ↓; TLR3 ↑, MLKL ↑, HMGB1 ↑, NDRG2 ↑	Human	Stage I	[35]
	ApoE^−/−^ mice	M1 markers (CD68, MCP-1, iNOS); M2 markers (Arg-1, IL-10); complement C3, lipoprotein lipase, LDLR-associated protein 1, TFR	M1 markers ↑ (IgE promotes M1-like polarization); M2 markers ↓; complement C3 ↑	Mouse (ApoE^−/−^)	Stage I	[36]
	CHD patients, ApoE^−/−^ mice, RAW264.7 macrophages	TXNIP, IL-1β, IL-18	TXNIP ↑, IL-1β ↑, IL-18 ↑ (nicotine exacerbates atherosclerosis)	Human + Mouse + in vitro	Stage I	[37]
	CHD patients (AMI)	TBK1, HIF-1α, IL-17/IL-10	TBK1 ↑, HIF-1α ↑, IL-17 ↑, IL-10 ↓ (aggravated by hyperglycemia)	Human + in vitro	Stage II	[38]
	C57BL/6J (WT) and PCSK9^−^/^−^ mice	PCSK9	PCSK9 deficiency → M2 polarization ↑, improved ventricular remodeling	Mouse	Stage III	[39]
	SD MI rats and RAW264.7 macrophages	Gamma-aminobutyric acid (GABA); M2 macrophages	GABA → M2 polarization ↑; improved sympathetic nerve remodeling	Rat + in vitro	Stage III	[40]
Eosinophils	CHD patients	Percentage of eosinophils	Eosinophils ↑, associated with coronary artery disease	Human	Stage I	[41]
	CHD patients	Activated mast cells positively correlated with eosinophils; eosinophils are involved in the immune inflammatory network of CHD	Eosinophils ↑, positively correlated with activated mast cells	Human	Stage I	[42]
	CHD patients (AMI)	Eotaxin-1/eosinophils were associated with CAD independent of known risk factors; Eotaxin-1 may assist in AMI diagnosis	Eotaxin-1 ↑, independently associated with CAD	Human	Stage II	[43]
	patients with STEMI	Percentage of eosinophils	Eosinophil percentage ↓ → predictor of poor prognosis	Human	Stage II	[44]
	ΔdblGATA mice and MI patients	IL-4, mEar1	Eosinophils → IL-4 ↑, mEar1 ↑ → cardiac function improved	Mouse + Human	Stage III	[45]
	C57BL/6 and eosinophil-KO (Gata1 KO) mice; human pericardial fluid cells	Eosinophils and IL-4	Eosinophils ↑ → IL-4 ↑ → post-infarction repair ↑	Mouse + Human	Stage III	[46]
	ΔdblGATA mice and MI patients	Eosinophil count; eosinophilic cationic protein (ECP) concentration	ECP ↑ → promotes vascular calcification and atherogenesis	Mouse + Human	Cross-stage	[47]
Basophils	CHD patients and controls (no stenosis or <30%)	Neutrophil-to-HDL-C ratio (NHR)	NHR ↑, positively correlated with stable CAD severity	Human	Stage I	[48]
	CHD patients	GO enrichment: neutrophil degranulation; hub genes: LYZ, CAMP, HP, ORM1, LTF; neutrophils increased	Neutrophils ↑; LYZ ↑, CAMP ↑, HP ↑, ORM1 ↑, LTF ↑	Human	Stage I	[42]
	CHD patients (AMI)	Neutrophil peptide-1, -2, and -3	HNP 1-3 ↑, correlated with coronary lesion severity	Human	Stage II	[49]
	AMI patients	S100A12, AQP9, CSF3R, S100A9, CD14	S100A12 ↑, AQP9 ↑, CSF3R ↑, S100A9 ↑, CD14 ↑ (AMI biomarkers)	Human	Stage II	[50]
	C57/BL gene knockout mice	IL-10, TNF-α, Th1, Th2	iNKT deficiency → IL-10 ↓, TNF-α ↑; Th1/Th2 imbalance → cardiac hypertrophy aggravated	Mouse	Stage III	[51]
	AMI patients	Neutrophils, eosinophils, lymphocytes, monocytes, and basophils	Basophil changes were associated with short-term prognosis of cardiogenic shock; leukocyte subset distribution altered	Human	Stage III	[52]
	UK Biobank cohort	White blood cells and lipids	Basophil counts were significantly associated with serum lipid levels	Human	Cross-stage	[53]
	Patients with and without CVD	WBC counts and neutrophils	Neutrophils ↑, positively associated with CVD and all-cause mortality	Human	Cross-stage	[54]
	CHD patients	NLR and Fibrosis-4 index	NLR ↑, FIB-4 ↑ → predicted all-cause mortality in CHD patients	Human	Cross-stage	[55]
	Chronic stable CAD, UA, NSTEMI, and STEMI	Neutrophil granulocytes	Neutrophil phenotypes differ across disease stages	Human	Cross-stage	[56]
Mast cells	EAT and SAT: CHD patients vs. 21 non-coronary patients	Mast cells	Mast cells ↑ (significantly increased in EAT of CHD patients, associated with inflammation)	Human	Stage I	[57]
	EAT, SAT, and RA in CHD patients	Mast cell count, CD117, chymase	Mast cell count ↑ (in EAT); CD117 ↑, chymase ↑	Human	Stage II	[58]
	CHD patients (NSTEMI and stable CHD)	Carboxypeptidase A3, tryptase G, tryptase 1	Carboxypeptidase A3 ↓ (decreased in advanced CAD patients)	Human	Cross-stage	[59]
NK cells	CHD patients	CD56dim NK cells decreased; candidate markers: CHFR, CEL, CCDC28A	CD56 DIM NK cells ↓; CHFR ↑/CEL ↑/CCDC28A ↑ (candidate biomarkers)	Human	Stage I	[60]
Dendritic cells	CHD patients	cDCs and pDCs	cDCs and pDCs altered in adipose tissue of CHD patients	Human	Stage I	[61]
	ApoE^−/−^ mice	CD86, CD40, MHC-II, Th1 and Th17, and ox-LDL	CD86 ↑, CD40 ↑, MHC-II ↑ → DC activation → Th1/Th17 ↑ → AS aggravated	Mouse (ApoE^−/−^)	Stage I	[62]
	CHD patients	Plasmacytoid DCs decreased; coexists with T-cell and B-cell immune remodeling	pDCs ↓; T/B-cell immune remodeling	Human	Cross-stage	[63]
T cells	CHD patients and CD4^+^ Th cells	IL-5, IL-17, IFN-γ, Th1, Tregs, Th17	IL-5 ↓; Th1 ↑, Th17 ↑; Tregs altered; IL-17 ↑, IFN-γ ↑	Human + in vitro	Stage I	[64]
	CHD patients	γδ T, NK cells, LDL-C	γδ T phenotypic changes ↓ (downregulation of activating molecules and co-stimulatory molecules)	Human	Stage I	[65]
	CHD patients	Tregs	Treg imbalance (RNA sequencing confirmed Treg pathway abnormalities)	Human	Stage I	[66]
	CHD patients	T-cell receptor (TCR) and CDR3	TCR diversity restricted ↓ (in epicardial adipose tissue of Npatients with STEMI)	Human	Stage II	[67]
	Spleen CD4^+^ T cells and THP-1 macrophages in HF rat/mouse models	Foxp3^+^ Tregs, CD4^+^ T cells	Tregs ↑ (after herbal medicine intervention); NLRP3 inflammasome inhibited	Rat/Mouse	Stage III	[68]
	CHD patients	Alterations in 15 T-cell subsets; Treg expression of CCR2, CCR4, CD38, CD45RO, HLA-DR, Ki67 were associated with increased CHD risk	Multiple T-cell subsets altered; Treg activation markers ↑ (CCR2, CCR4, CD38, CD45RO, HLA-DR, Ki67)	Human	Cross-stage	[63]
	CHD patients	Decreased T cells in iCHD and ASCHD; decreased DCs, lymphocytes, CD8^+^CD28^+^ T cells, CD57^+^ T cells; increased Tregs in iCHD; enhanced T-cell cytotoxicity in ASCHD	T cells ↓, DCs ↓, CD8^+^CD28^+^ ↓, CD57^+^ ↓; Tregs ↑ (iCHD); T-cell cytotoxicity ↑ (ASCHD)	Human	Cross-stage	[34]
	CHD patients	B-cell Receptor (BCR)	BCR diversity altered (clonal diversity changes in coronary in-stent restenosis patients)	Human	Stage I	[69]
	CHD patients	Memory B cells negatively correlated with naïve B cells; imbalanced B-cell subset distribution	Memory B cells ↑, naïve B cells ↓ (subset imbalance)	Human	Stage I	[42]
	CHD patients	Bregs	Bregs altered in AMI patients (pilot study)	Human	Stage II	[70]
	CHD patients	Increased markers of proliferation and differentiation in B cells; adaptive immune remodeling	B-cell proliferation ↑, differentiation markers ↑	Human	Cross-stage	[63]

Abbreviations and symbols: ↑, increased or upregulated; ↓, decreased or downregulated; →, induces or promotes; M0/M1/M2.

### 1.6. Literature Search Strategy

This article is a narrative review aimed at providing a comprehensive synthesis of the regulatory role of immune cells in the occurrence and development of CHD. To ensure transparency and reproducibility of the literature search, the search strategy is detailed below.

#### 1.6.1. Search Databases

The following electronic databases were searched for this review: PubMed/MEDLINE, Web of Science Core Collection, Scopus, and the Cochrane Library. In addition, CNKI and Wanfang Data were searched for high-quality Chinese articles that may not be included in English databases and that address immunoregulatory studies involving traditional Chinese medicine (TCM) and natural compounds.

#### 1.6.2. Search Terms and Search Strategy

The search strategy was constructed around two core conceptual axes and combined using Boolean operators (AND/OR): Conceptual axis I—Disease domain: MeSH terms and free-text keywords included “coronary heart disease,” “coronary artery disease,” “atherosclerosis,” “acute coronary syndrome,” “myocardial infarction,” “stable angina,” “vulnerable plaque,” and “in-stent restenosis.”

Immunology domain: search terms included specific immune cell types (“monocytes,” “macrophages,” “neutrophils,” “eosinophils,” “basophils,” “dendritic cells,” “mast cells,” “natural killer cells,” “T lymphocytes,” “B lymphocytes,” “Th17 cells,” “regulatory T cells,” “γδ T cells”) and immune mechanisms/pathways (“NLRP3 inflammasome,” “immune regulation,” “inflammatory cytokines,” “clonal hematopoiesis,” “Mendelian randomization,” “oxidation-specific epitopes,” “neutrophil extracellular traps,” and “immune checkpoints”).

#### 1.6.3. Time Range

The primary search window spanned from January 2015 to August 2026 to ensure that the content of the review fully reflected the latest progress in the field of cardiovascular immunology over the past decade. Landmark studies integral to the theoretical framework of this review—including founding mechanistic studies (e.g., the early formulation of the atherosclerosis immune hypothesis), pivotal clinical trials (e.g., large randomized controlled trials targeting inflammatory pathways), and seminal methodological articles—were also included, even if they were published before 2015. The final search update was completed in August 2026.

#### 1.6.4. Literature Inclusion and Exclusion Criteria

The inclusion criteria included: (1) original research articles (including in vitro experiments, animal models and human studies) exploring the role of immune cells (innate and/or adaptive immunity) in the pathogenesis of CHD and atherosclerosis; (2) observational cohort and case–control studies that reported the association of peripheral blood immunophenotypes, circulating inflammatory markers, or immunogenetic variants with clinical outcomes of coronary artery disease (CAD); (3) genetic epidemiological studies employing genome-wide association studies (GWAS) to identify susceptibility loci or MR to assess putative causal relationships between immunophenotypes and CAD; (4) randomized controlled clinical trials of immunotherapy for CAD; and (5) high-quality systematic reviews and meta-analyses. The exclusion criteria were as follows: (1) studies published only in the form of conference abstracts without full text, (2) preprint papers without peer review, (3) case reports with unclear mechanistic descriptions or an incomplete evidence chain, and (4) studies published in neither English nor Chinese.

#### 1.6.5. Supplementary Search Methods

In addition to the systematic search of electronic databases, we used the following supplementary strategies to minimize the risk of missing relevant articles. (1) Backward citation tracking: the reference lists of the included key review articles and highly cited original studies were manually screened to identify relevant studies that were not captured by the electronic search. (2) Forward citation tracking (via Web of Science’s “Cited Reference Search” and Google Scholar) of landmark studies in the field, such as original papers that first reported the role of specific immune cell subsets in CHD, was performed to incorporate important subsequent studies citing these seminal works. (3) Field expert consultation: domain experts in cardiovascular immunology and interventional cardiology were consulted to obtain information on the latest important findings and key studies in the field.

#### 1.6.6. Statement of Methodological Limitations

It should be clearly stated that this was a narrative review and not a systematic review or meta-analysis. Therefore, it inevitably involves professional judgment and informed selection by the authors rather than fully following the standardized process of the Preferred Reporting Items for Systematic Reviews and Meta-Analyses guidelines. No formal risk of bias assessment or quantitative data synthesis was performed for the included studies. Readers should take these methodological limitations into account when evaluating the evidence presented in this review. Nonetheless, we sought to maximize the completeness of literature coverage and transparency of the selection process within the framework of a narrative review through the structured search strategy described above, clear inclusion/exclusion criteria, and multiple supplementary search methods.

## 2. Immune Cells in Atherosclerosis: An Overview

Atherosclerosis is initiated by the recruitment of circulating monocytes the arterial intima, differentiate into macrophages, and internalize cholesterol-rich oxidized low-density lipoproteins (ox-LDL) particles. This process ultimately results in the generation of lipid-laden foam cells, which represent the pathological hallmark of early fatty streak lesions [71]. The immune cells implicated in this atherogenic process can be broadly categorized into two major groups: innate immune cells and adaptive immune cells.

The innate immune compartment comprises monocytes, macrophages, neutrophils, eosinophils, basophils, natural killer (NK) cells, dendritic cells (DCs, including Langerhans cells and interstitial, circulating, follicular, plasmacytoid, and thymic DCs), and mast cells. The adaptive immune compartment comprises T lymphocytes and B lymphocytes. T cells are classified by surface markers into CD4^+^ (including helper T cells [Th] and regulatory T cells [Treg]) and CD8^+^ (primarily cytotoxic T cells [Tc]) subsets. Functionally, CD4^+^ T-cells are further subdivided into Th1, Th2, Th17, and Treg populations, each exerting distinct and sometimes opposing effects on atherogenesis. Furthermore, B cells, including regulatory B cells (Bregs), actively participate in immune regulation within atherosclerotic lesions [72,73,74]. The following sections discuss each major immune cell type in detail, beginning with monocytes and macrophages, which represent key innate immune effectors in CHD.

### 2.1. Immunological Staging of CHD: A Three-Stage Conceptual Framework

**Figure 1:**
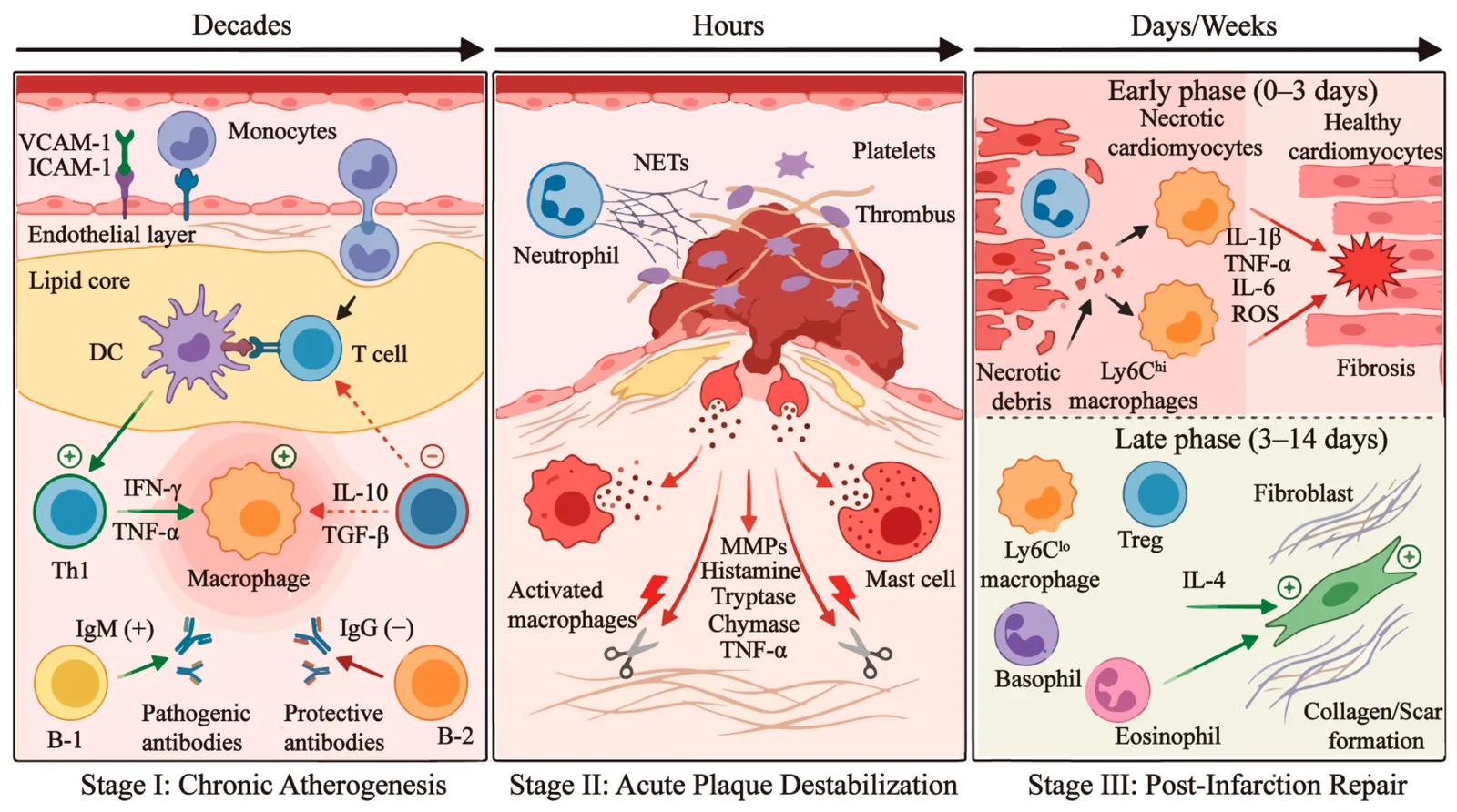
Dynamic immune remodeling across the three stages of coronary heart disease. Immune responses evolve in a stage- and time-dependent manner during the progression of coronary heart disease. Stage I: Chronic atherogenesis (decades). Endothelial activation and adhesion molecules, including VCAM-1 and ICAM-1, facilitate monocyte recruitment into the vascular wall, where monocytes differentiate into macrophages. Dendritic cells (DCs) activate T cells through antigen presentation. Th1 cells and macrophages produce pro-inflammatory mediators such as IFN-γ and TNF-α, whereas anti-inflammatory signals, including IL-10 and TGF-β, counterbalance excessive inflammation. Distinct B-cell subsets and antibody responses also contribute to the regulation of atherosclerotic progression. Stage II: Acute plaque destabilization (hours). Activated neutrophils release neutrophil extracellular traps (NETs), which cooperate with platelets to promote thrombus formation. Activated macrophages and mast cells release matrix metalloproteinases (MMPs), histamine, tryptase, chymase, TNF-α, and other inflammatory mediators, thereby promoting extracellular-matrix degradation, plaque rupture, and acute thrombosis. Stage III: Post-infarction repair (days to weeks). During the early phase (0–3 days), Ly6C^hi^ inflammatory macrophages participate in the clearance of necrotic debris, accompanied by increased production of IL-1β, TNF-α, IL-6, and reactive oxygen species (ROS). During the late phase (3–14 days), Ly6C^lo^ reparative macrophages, regulatory T cells (Tregs), basophils, and eosinophils contribute to inflammation resolution and promote fibroblast activation, collagen deposition, and scar formation, partly through IL-4-dependent signaling, thereby facilitating myocardial repair and remodeling. Different arrow types represent distinct biological interactions: solid arrows indicate direct regulation, while dashed arrows denote indirect or regulatory crosstalk. The symbol “+” indicates promotion or activation, and “−” indicates inhibition. Green arrows denote activating, promoting, or reparative effects, whereas red arrows denote inhibitory, pathological, or pro-inflammatory effects.

Before examining the roles of individual immune cell types, it is essential to establish a conceptual framework that explicitly distinguishes the three major pathological stages of CHD, as each stage is governed by fundamentally different immunological processes, involves distinct microenvironments, and engages the same immune cell types in different functional programs. Failure to delineate these stages risks conflating wound-healing functions with plaque-promoting functions and misattributing findings from one pathological context to another. In Section 2 and Section 3, each major finding is annotated with its corresponding stage to ensure interpretive clarity (Figure 1).

#### 2.1.1. Stage I—Chronic Atherogenesis (Plaque Initiation and Progression)

Stage I encompasses the decade-long process of arterial plaque initiation, lipid accumulation, foam cell formation, neointimal expansion, and fibrous cap development. The principal immunological features of this stage include: (a) endothelial activation and expression of adhesion molecules (VCAM-1 and ICAM-1) in response to modified lipoproteins and disturbed shear stress; (b) monocyte recruitment, subendothelial transmigration, and differentiation into lesional macrophages, which further polarize into pro-inflammatory (M1-like) or tissue-reparative (M2-like) phenotypes depending on the local cytokine milieu [59,60]; (c) T-cell infiltration, predominantly CD4^+^ Th1 cells that secrete IFN-γ and TNF-α to amplify macrophage activation, counterbalanced by Tregs that secrete IL-10 and TGF-β to restrain excessive inflammation [59]; (d) B-cell-mediated recognition of OSEs on modified LDL, with protective natural IgM antibodies (B-1 cells) versus potentially pathogenic IgG responses (follicular B-2 cells) [75]; and (e) dendritic cell antigen presentation linking innate recognition of danger signals to adaptive immune activation within the arterial wall and draining lymph nodes.

The principal experimental models for Stage I are ApoE^−/−^ and Ldlr^−/−^ mice maintained on high-fat or Western-type diets. These models robustly reproduce key features of the chronic inflammatory process of atherogenesis and have been instrumental in elucidating the roles of virtually all immune cell types in plaque biology. However, a critical limitation must be explicitly acknowledged: standard ApoE^−/−^ and Ldlr^−/−^ mice do not spontaneously develop plaque rupture or intraluminal thrombosis (Stage II events), nor do they experience myocardial infarction (Stage III). Consequently, findings from these models should be interpreted strictly within the context of Stage I pathobiology. In the human clinical setting, Stage I corresponds predominantly to stable CAD, including chronic stable angina and subclinical atherosclerosis, as detected by coronary calcium scoring or intravascular imaging.

#### 2.1.2. Stage II—Acute Plaque Destabilization and Rupture

Stage II represents the acute transition from a stable plaque to an unstable, thrombosis-prone lesion. Key pathological features include fibrous cap thinning (driven by matrix metalloproteinase [MMP] overexpression from activated macrophages and mast cells), intraplaque hemorrhage (often secondary to neovascularization from the fragile vasa vasorum), plaque rupture or erosion with consequent exposure of the thrombogenic lipid core to flowing blood, and platelet–fibrin thrombus formation. The principal immunological features of Stage II include: (a) release of histamine, tryptase, chymase, and TNF-α, which collectively promote extracellular matrix degradation and endothelial apoptosis; (b) neutrophil extracellular trap formation at the rupture site, providing a scaffold for thrombosis and amplifying local inflammation [76]; and (c) sudden activation of the complement cascade and coagulation–inflammation crosstalk.

Stage II is the most poorly modeled stage in experimental cardiovascular research. Standard murine atherosclerosis models (ApoE^−/−^, Ldlr^−/−^) develop advanced plaques but essentially do not undergo spontaneous rupture with luminal thrombosis in a manner analogous to human acute coronary syndrome (ACS). Emerging models that partially address this gap include the tandem stenosis model (which induces plaque instability through hemodynamic manipulation) and the ApoE^−/−^Fbn1C1039G^+/−^ mouse (which develops spontaneous plaque rupture due to defective fibrillin-1 and consequent vascular fragility); however, the immunological characterization of these models remains in its early stages. Much of the immunological evidence for Stage II is derived from human studies, including analyses of thrombus aspirates from patients with STEMI, histological examination of culprit lesions from sudden cardiac death victims, and intracoronary imaging studies. In the clinical setting, Stage II corresponds to ACS (unstable angina, NSTEMI, and STEMI).

#### 2.1.3. Stage III—Post-Infarction Myocardial Repair and Remodeling

Stage III encompasses the immunological response to established myocardial infarction, including cardiomyocyte necrosis, sterile inflammation, inflammatory cell infiltration, granulation tissue formation, fibrotic scar maturation, and ventricular remodeling. Unlike Stage I (where immune processes occur over decades within the arterial wall) and Stage II (which unfolds over hours at the plaque–thrombus interface), Stage III involves a temporally orchestrated biphasic immune response within the myocardium itself:

Early inflammatory phase (days 0–3 post-MI in mice): Extensive infiltration of neutrophils and pro-inflammatory Ly6C^hi^ monocytes/macrophages, which phagocytose necrotic debris but also cause collateral tissue damage through reactive oxygen species, proteases, and pro-inflammatory cytokines (IL-1β, TNF-α, IL-6).

Late reparative phase (3–14 days post-MI in mice): Transition to reparative Ly6C^lo^ macrophages (M2-like), accumulation of Tregs, eosinophil-derived IL-4 secretion—which promotes fibroblast activation and collagen deposition—and basophil-mediated reparative signaling. This phase is essential for adaptive scar formation and the prevention of ventricular rupture.

The principal experimental models for Stage III are as follows: (a) surgical LAD coronary artery ligation (permanent occlusion or transient ischemia–reperfusion), which produces a focal infarct with a well-defined border zone, and (b) isoproterenol injection, which induces diffuse subendocardial necrosis through catecholamine-mediated oxidative stress and calcium overload, without any vascular pathology. It is critical to recognize that neither model involves atherosclerosis or plaque rupture. LAD ligation produces an ischemic injury that mimics the downstream consequences of coronary occlusion (which might occur after Stage II plaque rupture) but bypasses the atherosclerotic process entirely. Isoproterenol injury remains a reductionist model, modeling only the myocardial necrotic response without any vascular components. Findings from these models should, therefore, be interpreted strictly as reflecting the immunology of myocardial wound healing, rather than the immunology of plaque biology. In the clinical setting, Stage III corresponds to the post-MI period, including the acute and subacute phases of myocardial healing and long-term ventricular remodeling.

#### 2.1.4. Implications for Interpreting This Review

This three-stage framework resolves several apparent contradictions that arise when Stage I (plaque), II (rupture), and III (repair) findings are conflated. For example:

Eosinophil IL-4 and ECP secretion (Section Dual and Conflicting Roles in Myocardial Infarction): This represents a Stage III wound-healing function in the infarcted myocardium. It does not, however, imply that eosinophils play the same IL-4-driven role in atherosclerotic plaques (Stage I), where their contribution is mechanistically distinct and less well-characterized.

Basophil-driven reparative fibrosis (Section Basophil-Driven Reparative Fibrosis): Basophil-driven reparative fibrosis is a Stage III phenomenon observed in murine LAD ligation models. Evidence of basophil involvement in chronic atherogenesis (Stage I) is primarily derived from Mendelian randomization studies of peripheral basophil counts, which represent a fundamentally different line of evidence.

Effects of Tregs on post-infarction healing (Section 3.2.3): Tregs suppress excessive inflammation and promote adaptive remodeling in Stage III. In Stage I, Tregs also exert atheroprotective effects, but through different mechanisms (suppression of Th1/Th17 effector responses within the plaque and IL-10-mediated macrophage deactivation) and in a different microenvironment (arterial intima versus infarcted myocardium).

Readers are encouraged to see the stage annotations provided in Section 2 and when evaluating the evidence and exercise caution before extrapolating findings across stages without explicit mechanistic justification.

#### 2.1.5. A Note on the “Redistribution” Hypothesis

Throughout this review, peripheral blood cytopenias observed in patients with CHD (e.g., eosinopenia in AMI, basophil count reduction in ACS, and NK cell count decrease in acute MI) are discussed in relation to a hypothesized “redistribution” mechanism, whereby circulating immune cells are recruited from the peripheral blood into affected tissues (atherosclerotic plaques or infarcted myocardium), resulting in a measurable decline in circulating counts. While this hypothesis is biologically plausible and consistent with the known leukocyte trafficking physiology, it is important to note that none of the clinical studies cited in support of this interpretation simultaneously measured peripheral blood cell counts and tissue-level immune cell infiltration in the same subjects. Therefore, the redistribution explanation remains an unvalidated hypothesis. Alternative mechanisms that could account for peripheral cytopenias include (a) stress-induced bone marrow suppression or altered hematopoietic output, (b) consumptive depletion (activation-induced cell death) within the circulation, (c) splenic sequestration, and (d) apoptosis-mediated clearance. Definitive resolution of this question will require prospective studies employing paired blood and tissue sampling (e.g., endomyocardial biopsy or thrombus aspirate analysis combined with simultaneous peripheral blood immunophenotyping) or advanced in vivo cell-tracking methodologies (e.g., deuterium-labeled leukocyte studies). All three instances where the redistribution explanation is invoked in this review (Section 2.5.1, Section 2.5.2 and Section 2.6) have been annotated with the qualifier “hypothesized” to alert readers to this evidentiary limitation.

### 2.2. Monocytes

#### 2.2.1. Biology and Role in Atherogenesis

Monocytes are key effectors of the innate immune system and serve as important mediators of atherogenesis and atherothrombosis. Following their development in the bone marrow, monocytes circulate in the bloodstream and are recruited to sites of vascular injury. Upon entering the tissue, they differentiate into macrophages with potent phagocytic activity [77,78]. In recent years, advances in high-throughput sequencing, proteomics, and machine learning have substantially reshaped our understanding of monocytes in CHD. These approaches have shifted their classification from relatively homogeneous pro-inflammatory population to highly heterogeneous effector populations. Consequently, monocyte-derived signatures now serve dual roles: as potential biomarkers of disease progression and as important regulators influencing plaque stability, myocardial fibrosis, and the risk of acute coronary events.

#### 2.2.2. Monocyte Subsets and Their Functional Heterogeneity

Human monocytes are classified based on CD14/CD16 expression patterns into three subsets: classical (CD14^++^CD16^−^), intermediate (CD14^++^CD16^+^), and non-classical (CD14^+^CD16^++^), exhibit distinct functional properties in CHD pathogenesis. The expansion of CD14^+^CD16^++^ non-classical monocytes is associated with vascular endothelial dysfunction and oxidative stress; however, accumulating evidence suggests, these cells may also participate in immune surveillance and help prevent excessive inflammatory activation, thereby limiting AS exacerbation [76,79]. Recent prospective evidence indicates that increased levels of intermediate monocytes are specifically associated with an increased risk of incident major adverse cardiovascular events (MACE) in patients with stable coronary artery disease [80]. Furthermore, marked sex-related differences in monocyte signaling pathways and subset distribution have been identified, suggesting that biological sex significantly modulates monocyte-mediated immune responses in CHD pathogenesis [81].

#### 2.2.3. Chemokine-Dependent Monocyte Recruitment

The migration of monocytes into the arterial intima is orchestrated by inflammatory chemokine receptor–ligand interactions/pathways, particularly CCR2–CCL2 and CCR5–CCL5 [82,83]. Autopsy studies have demonstrated elevated CCL2 expression in the intima of atherosclerotic arteries along with accompanied by enhanced expression of its cognate receptor CCR2 in diseased arteries [84]. Studies in genetically modified mice have confirmed that genetic deletion or pharmacological inhibition of these chemokine axes reduces atherosclerotic lesion development inhibition [85]. In ApoE^−/−^ mouse models, insulin-like growth factor-1 receptor (IGF1R) was found to be highly expressed in lesional macrophages, suggesting growth factor signaling pathways may represent as an additional regulatory layer in atherogenesis [86].

#### 2.2.4. Monocyte-Based Clinical Biomarkers

In the clinical setting, research has increasingly focused on peripheral blood monocyte-derived inflammatory indices because of their cost-effectiveness and accessibility. The monocyte-to-high-density lipoprotein (HDL) cholesterol ratio (MHR) and monocyte-to-lymphocyte ratio (MLR) were independently associated with the severity of coronary stenosis and carotid plaque instability in multivariable-adjusted analyses [87]. However, the degree of confounding adjustment varied across studies evaluating these ratios. While the principal studies cited here employed multivariable regression adjusting for age, sex, hypertension, diabetes mellitus, smoking, and LDL cholesterol, adjustments for renal function (estimated glomerular filtration rate; eGFR), body mass index (BMI), and concurrent medications (statins and antiplatelet agents) are inconsistent. Moreover, formal incremental discrimination analyses—such as change in C-statistic (ΔC), net reclassification improvement (NRI), or integrated discrimination improvement (IDI)—evaluating whether MHR or MLR improves risk prediction beyond established cardiovascular risk models (e.g., Framingham Risk Score) have not been reported in the studies cited. Consequently, these ratios should be regarded as associative biomarkers reflecting monocyte-driven inflammatory status rather than as validated additions to clinical risk prediction algorithms.

An important methodological consideration is that MHR incorporates HDL cholesterol as its denominator. Because low HDL-C is an established cardiovascular risk factor with a well-characterized inverse relationship with atherosclerotic burden, MHR inherently captures a component of lipid risk in addition to monocyte-mediated inflammation. This limitation is shared by the neutrophil-to-HDL ratio (NHR) discussed in Section 2.4.4. To disentangle the inflammatory information contributed by the monocyte count numerator from the lipid metabolic information contributed by the HDL-C denominator, future studies should include HDL-C as a separate covariate in multivariable models and explicitly compare the discriminative performance of the composite ratio versus its individual components. Recent retrospective studies have demonstrated that the integration of triglyceride–glucose (TyG) index with the systemic inflammatory response index (SIRI), which incorporates monocyte count as one of its components, may improve the diagnostic performance for coronary artery stenosis in patients with acute coronary syndrome (ACS) [88]. In patients with chronic total occlusion (CTO), an elevated monocyte-to-high-density lipoprotein cholesterol ratio (MHR) is independently associated with impaired coronary collateral circulation [89]. Moreover, dynamic changes in monocyte counts after percutaneous coronary intervention (PCI) have been associated with long-term cardiovascular outcomes [90].

#### 2.2.5. Intracellular Pathways Driving Monocyte Pathogenicity

Mechanistic studies over the past five years have identified multiple intracellular pathways that involved in regulating monocyte/macrophage pathogenicity in CHD: Ferroptosis and autophagy: The long non-coding RNA (lncRNA) NEAT1 promotes macrophage ferroptosis and aggravates atherosclerosis through STAT3-dependent signaling [91]. In parallel, DNA methylation-mediated regulation of galectin-8 has been reported to modulate macrophage autophagy through the MAPK/mTOR axis, thereby attenuating lesion progression [92]. Intercellular crosstalk: A complex NF-κB pathway-dependent interaction between vascular smooth muscle cells and monocytes has been identified as a implicated in the regulation of plaque stability [93]. Metabolic regulation: The glycolytic regulatory gene *Pfkfb3* exhibits a gene dosage effect in monocyte biology, linking cellular metabolic activity to the vascular inflammatory responses [94]. Additionally, the plasma protein SECTM1 has been reported to regulate monocyte levels and to correlate positively with CHD risk [95].

#### 2.2.6. Bioinformatics and Machine Learning Approaches

Advances in computational biology have facilitated the identification of monocyte-related diagnostic and prognostic signatures. By combining gene co-expression network analysis (WGCNA) with machine learning algorithms (including XGBoost and LASSO regression), researchers have identified specific DNA methylation-regulated genes as genes with potential predictive value for acute myocardial infarction (AMI) [96]. A novel four-gene signature reflecting myeloid cell immune infiltration has also been developed and internally validated for prediction of heart failure risk in patients with STEMI [97]. In older patients, machine learning models incorporating mononuclear cell-related features have been applied to predict risk of unplanned hospital readmission [98].

#### 2.2.7. Environmental Exposure, Comorbidities, and Therapeutic Implications

Environmental exposures and comorbid conditions may influence CHD risk through monocyte-mediated immune mechanisms. Microplastic exposure has been associated with monocyte-associated inflammatory responses and greater complexity of coronary lesion morphology [99]. In patients undergoing maintenance dialysis, has been reported to attenuate the association between elevated MLR and aortic calcification [100]. Variations in MCP-1 levels have been associated with clinical outcomes in CHD patients and may be influenced by nutritional status [101]. In preclinical studies and preliminary clinical investigations, traditional Chinese medicine formulations such as Compound Danshen Dripping Pills have shown potential to preserve endothelial function and reduce myocardial fibrosis associated with in-stent restenosis possibly through suppression of monocyte-mediated inflammatory pathways [102,103]. Taken together, research over the past five years has substantially expanded the conventional view of monocytes, from relatively simple inflammatory mediators to highly regulated, heterogeneous effector populations in CHD. Integrating inflammatory ratios (MHR, SIRI), epigenetic markers, and machine learning models into a multidimensional monocyte evaluation system has the potential to provide a multidimensional framework for precision diagnosis and targeted intervention in CHD.

### 2.3. Macrophages

#### 2.3.1. Origin, Polarization, and Functional Heterogeneity

As key components of the immune microenvironment in CHD and atherosclerosis, macrophages exhibit functional heterogeneity across the disease continuum—from plaque initiation and progression to rupture and post-MI repair. Several pattern recognition receptors (PRRs) expressed by macrophages recognize oxidized low-density lipoprotein (ox-LDL) and other damage-associated molecular patterns (DAMPs), thereby promoting pro-inflammatory cytokine release and activating downstream inflammatory cascades [104].

Macrophages within atherosclerotic lesions arise primarily from two sources: tissue-resident macrophages present within the vascular wall, and monocyte-derived macrophages recruited from the circulation to the lesion site [105]. The local microenvironment and cytokine milieu shape macrophage activation states, traditionally been described according to the M1/M2 polarization framework [106]. However, this binary classification is now recognized as an oversimplification; single-cell transcriptomic studies have identified additional macrophage states (e.g., Mox, M4, and Mhem) that highlighting the continuous and context-dependent nature of macrophage activation in vivo.

M1, or classically activated, macrophages are induced by pro-inflammatory stimuli such as interferon-γ (IFN-γ) and lipopolysaccharide (LPS), and produce mediators including interleukin-6 (IL-6), IL-12, tumor necrosis factor-α (TNF-α), and reactive oxygen species (ROS) [107,108]. M2 (alternatively activated) induced predominantly by the T-helper 2 (Th2) cytokines IL-4 and IL-13 [109] and are generally associated with the production of anti-inflammatory mediators IL-10 and transforming growth factor-β (TGF-β), thereby contributing to immune regulation, resolution of inflammation, and tissue repair [110].

#### 2.3.2. Clinical Evidence Linking Macrophage Phenotypes to Plaque Vulnerability

The phenotypic plasticity of macrophages is central role in regulating CHD progression. A study examining the association between in vivo coronary plaque characteristics assessed by optical coherence tomography (OCT) and morphological profiles of monocyte-derived macrophage (MDMs) demonstrated that plaque macrophage accumulation was more frequently observed in patients with a higher proportion of circular MDM, a morphological phenotype associated with a pro-inflammatory state [27]. In a retrospective analysis of 1867 participants, the monocyte-to-HDL-cholesterol ratio (MHR) was significantly higher in patients with CAD than in control participants [111]. Furthermore, analysis of epicardial, pericardial, and subcutaneous adipose tissue biopsies from patients undergoing coronary artery bypass grafting (CABG) or aortic valve replacement showed that altered circulating levels and transcriptomic signatures of CD206, NOS2, and galectin-9 (Gal-9) were associated with clinical status in CHD, with macrophage polarization in epicardial adipose tissue (EAT) appearing to be particularly relevant [112].

Recent studies have proposed that macrophage phagocytic capacity may serve as an potential biomarker for predicting the risk of CHD and acute coronary syndrome (ACS) [113]. Furthermore, detailed characterization of markers related to M1-like polarization and efferocytosis has may provide a molecular basis for CHD risk stratification [114]. During MI and myocardial ischemia–reperfusion injury (MIRI), macrophages exert context-dependent effects in tissue damage and repair; the balance between the early inflammatory responses and subsequent reparative processes is a may influence clinical outcomes [115].

#### 2.3.3. Core Regulatory Pathways

Non-coding RNAs and ferroptosis. The long non-coding RNA (lncRNA) NEAT1 has been shown to promote macrophage ferroptosis through STAT3 activation, thereby exacerbating atherosclerotic progression [91]. In parallel, miR-127-3p has been reported to enhance and promotes lesion evolution by altering fatty acid metabolism and oxidative phosphorylation [116].

Epigenetic regulation. Epigenetically mediated upregulation of CLEC5A has been implicated in monocyte/macrophage dysfunction in CHD patients [117]. Bioinformatics analysis of lipid metabolism-related gene networks has further provided insight into how metabolic stress may contribute to CHD pathogenesis via macrophage reprogramming [118]. Genetic susceptibility. A meta-analysis has supported an association between polymorphisms in the macrophage migration inhibitory factor (MIF) gene—notably rs755622—and susceptibility to CHD [119].

#### 2.3.4. Macrophage-Derived Extracellular Vesicles and Intercellular Communication

Macrophage-derived extracellular vesicles (EVs), including exosomes, have emerged as important mediators for intercellular communication that in cardiovascular disease [120]. Notably, environmental toxins such as microcystin-LR have been shown to influence cardiovascular risk by altering macrophage polarization through extracellular vesicle-mediated intercellular signaling, suggesting a potential mechanistic link between environmental pollution and perturbation of the cardiovascular immune homeostasis [121].

#### 2.3.5. Macrophage–Adipose Tissue Crosstalk

The interaction between macrophages and surrounding tissues—particularly epicardial adipose tissue (EAT)—have attracted increasing attention. Excessive EAT accumulation has been associated with microvascular obstruction following myocardial ischemia–reperfusion potentially through the promotion of a pro-inflammatory phenotype [122]. Computational approaches have further characterized macrophage infiltration patterns in EAT of CHD patients, identified associations with plaque vulnerability [123]. Multimodal imaging combining coronary computed tomography angiography (CCTA) and OCT has demonstrated a associations among pericoronary adipose tissue attenuation, macrophage infiltration, and adverse clinical outcomes [124].

#### 2.3.6. Emerging Therapeutic Strategies Targeting Macrophages

Therapeutic strategies targeting macrophage-mediated pathways are increasingly being explored for translational and clinical applications. Novel dual-gas-delivery nanomedicines designed for the plaque microenvironment designed to respond to the physicochemical milieu of lesion sites and enhance site-specific therapeutic effects by modulating macrophage activation states [125]. randomized controlled trials, such as the SPECIAL trial, are evaluating the effects of the proprotein convertase subtilisin/kexin type 9 (PCSK9) inhibitor evolocumab on macrophage burden within non-culprit coronary lesions, with the potential to improve long-term outcomes in ACS patients by reducing macrophage-associated inflammation [126].

In summary, our understanding of macrophages in CHD has evolved from conventional inflammatory classifications to encompass a complex framework encompassing metabolic, epigenetic, and intercellular mechanisms. Integrating macrophage activation states, efferocytic function, and adipose tissue-associated immune features into multi-omics prognostic models may provide new opportunities for early intervention and precision therapy of CHD.

#### 2.3.7. Clonal Hematopoiesis of Indeterminate Potential (CHIP) and Macrophage-Driven Atherogenesis

An emerging paradigm linking hematopoietic stem cell biology to macrophage-driven cardiovascular inflammation is CHIP. CHIP is characterized by the acquisition of somatic mutations in hematopoietic stem cells, most commonly in epigenetic regulators such as TET2, DNMT3A, and ASXL1, or in signaling molecules such as JAK2, which confer a selective proliferative advantage, leading to the clonal expansion of mutant leukocyte populations in the absence of overt hematological malignancies. CHIP prevalence increases with age, affecting more than 10% of individuals older than 70 years, and population-based studies have demonstrated that CHIP carriers harbor a 1.9- to 4.0-fold elevated risk of CHD and myocardial infarction independent of traditional cardiovascular risk factors [127].

The mechanistic basis of this association centers on macrophage inflammatory reprogramming. In murine models, Tet2-deficient bone marrow-derived macrophages exhibit enhanced NLRP3 inflammasome activation, leading to increased secretion of IL-1β and IL-6 and accelerated atherosclerotic lesion development [128]. *DNMT3A* mutations promote macrophage-mediated vascular inflammation by altering DNA methylation patterns at inflammatory gene loci. These findings directly implicate CHIP-driven macrophage dysfunction—via the NLRP3 inflammasome–IL-1β–IL-6 axis—as a cellular origin for the “residual inflammatory risk” observed in a substantial proportion of cardiovascular patients despite optimal lipid-lowering therapy. Clinically, CHIP carriers exhibit elevated high-sensitivity C-reactive protein (hsCRP) and IL-6 levels, and the CHIP status represents a potential confounder in observational studies examining monocyte/macrophage phenotypes and CHD outcomes without accounting for clonal hematopoiesis.

### 2.4. Neutrophils

#### 2.4.1. Overview: From Acute Inflammatory Effectors to Immune Regulatory Hubs

Neutrophils constitute the most abundant leukocyte population in peripheral blood. Although neutrophils were long regarded as transient effectors of acute inflammation, our understanding of their role in CHD has substantially expanded over the past decade. Neutrophils are now understood to be important components in a complex immune regulatory network that involved throughout the course of atherosclerosis—from plaque initiation and progression to plaque destabilization to post-interventional complications. Recent advances have provided evidence supporting a potential causal contribution between neutrophil-related inflammatory traits and CHD using genetic approaches and have yielded a range of neutrophil-based composite inflammatory markers that serve as tools for risk stratification, severity assessment, and prediction of adverse outcomes after coronary intervention.

#### 2.4.2. Genetic Evidence for a Causal Role in CHD

Mendelian randomization (MR) studies have provided genetic evidence for the potential causal association between neutrophil-related inflammatory traits and CHD in CHD. Cupido et al. reported that a genetically predicted higher NLR was associated with an increased risk between genetically predicted higher neutrophil-to-lymphocyte ratio (NLR) and CHD risk, suggesting a possible causal contribution of systemic inflammation reflected by NLR in atherogenesis [129]. This genetic evidence complements observational data showing that NLR is associated with the severity of coronary artery disease [130] and may independently predict of cardiac mortality [131]. As a relatively inexpensive and readily obtainable biomarker, NLR has potential clinical utility; however, further prospective and mechanistic studies are required to clarify its biological significance and clinical applicability.

#### 2.4.3. Mechanisms of Neutrophil-Mediated Plaque Development and Destabilization

Accumulating evidence indicates that neutrophils actively contribute to atherosclerotic plaque development and destabilization [132]. Following tissue injury or inflammatory stimulation, the alterations in the local cytokine and chemokine milieu can disrupt neutrophil homeostasis [133]. Under conditions of persistent inflammation, activated neutrophils accumulate at vascular lesions and release cytotoxic granule proteins and enzymes—including myeloperoxidase (MPO), lactoferrin, proteinase 3, matrix metalloproteinase-9 (MMP-9), and defensins that thereby contributing to tissue injury and sustained vascular inflammation [131,134]. These neutrophil-derived effector molecules may promote atherogenesis through several mechanisms: MPO participates in lipid peroxidation of LDL, promotes macrophage uptake of Ox-LDL to form foam cells, and thereby induces atherosclerotic plaque formation [135,136]. Neutrophil-associated lipid transporters form heterodimers with MMP-9, protecting MMP-9 from degradation and prolonging its extracellular matrix-degrading activity the extracellular matrix, which may weaken the fibrous cap and contribute to plaque instability [137]. Neutrophils interact with endothelial cells to stimulate chemokine secretion, which triggers a feed-forward loop of pro-inflammatory mediator release and sustained neutrophil activation, thereby amplifying the systemic inflammatory response [138,139].

A clinical study examining neutrophil numbers and phenotypes in 55 stable patients with chronic coronary syndrome (CCS) demonstrated that neutrophil activation is primarily reflected in the upregulation of the adhesion molecule CD11b, which facilitates neutrophil accumulation within coronary atherosclerotic plaques and contributes to plaque development and expansion [140]. Future studies exploring neutrophil surface molecules and biomarkers of plaque progression in larger CHD cohorts will provide further insights into the pathophysiological mechanisms underlying plaque instability and acute ischemic events.

#### 2.4.4. Neutrophil-Based Composite Inflammatory Markers for Clinical Risk Stratification

The combination of neutrophil count with other metabolic or immune parameters has shown significant associations with the severity of coronary artery lesions and has been proposed for clinical risk stratification, although formal evidence of incremental predictive value beyond established risk models remains limited for most indices.

NHR: NHR is significantly correlated with the degree of coronary artery stenosis in patients with stable CAD and has been proposed as a marker of plaque vulnerability [48]. As with MHR (Section 2.2.4), NHR incorporates HDL-C as its denominator, and its association with coronary disease severity may therefore partly recapitulate established lipid risk rather than provide purely independent information about neutrophil-mediated inflammation. Studies evaluating NHR should include HDL-C as a separate covariate to isolate the inflammatory contribution of the neutrophil count numerator. In patients undergoing PCI, elevated NHR levels are closely associated with the postoperative “no-reflow” phenomenon, reflecting the synergistic effects of abnormal lipid metabolism and neutrophil activation on microcirculatory dysfunction [141]. Neutrophil-to-albumin ratio (NAR): A prospective cohort study of 2990 patients demonstrated that NAR is independently associated with long-term mortality risk in patients with CHD in a multivariable-adjusted analysis, with discrimination metrics numerically exceeding those of single inflammatory markers [142]. However, the covariates adjusted in this study and whether formal incremental analyses (ΔC-statistic, NRI) were performed against established risk prediction models should be considered when interpreting these findings. Systemic immune-inflammation index (SII): In patients with chronic kidney disease (CKD), SII—which integrates neutrophil, platelet, and lymphocyte counts—correlates significantly with the Gensini score and has been proposed as a marker for assessing CAD severity in the context of complex comorbidities [143]. It should be noted that SII shares components with both NLR and PLR, creating potential collinearity when multiple ratios are evaluated simultaneously. Formal incremental discrimination analyses (ΔC-statistic, NRI) evaluating SII against established cardiovascular risk models have not been reported.

Combined metabolic–inflammatory indices: Integrating the triglyceride–glucose (TyG) index with inflammatory parameters, including neutrophil count has been reported to improve the prediction of all-cause and cardiovascular mortality in CHD patients [144]. Similarly, the combination of NLR and the LDL-C/HDL-C ratio has been associated with greater coronary lesion complexity in patients with acute coronary syndrome (ACS) [145].

Neutrophil-to-platelet ratio (NPR): In studies on long-term prognosis following PCI, NPR has been associated with in-hospital adverse events and poor long-term outcomes [146]. Collectively, these neutrophil-based composite indices reflect the integration of neutrophil-mediated innate immune activation with metabolic and other immune parameters and offer the advantages of low cost and routine availability. However, several methodological limitations remain. First, the degree of confounder adjustment varies substantially across studies. While most studies adjust for age, sex, and traditional cardiovascular risk factors, adjustment for renal function (eGFR), BMI, and concurrent medications is inconsistent. Second, for the majority of these ratios (NHR, NAR, SII, and NPR), formal incremental discrimination analyses (ΔC-statistic, NRI, and IDI) demonstrating added predictive value beyond established risk models have not been reported. Third, indices incorporating HDL-C as a denominator (NHR) partly recapitulate lipid risk, and indices sharing cellular components (SII vs. NLR vs. PLR) exhibit collinearity that complicates independent interpretations. Therefore, these ratios should be regarded as associative biomarkers of systemic immune-inflammatory status, the clinical predictive utility of which requires formal prospective validation with incremental discrimination analyses.

#### 2.4.5. Neutrophils in Acute Post-Infarction Complications

Beyond their involvement in chronic atherosclerotic plaque biology, neutrophils also contribute to in acute complications following MI. A recent mechanistic study revealed that following MI, neutrophil recruitment and activation after MI promoted an acute arrhythmogenic state characterized by electrical instability that promoted an acute arrhythmogenic state, whereas macrophages appeared to exert partially counter-regulatory effects [147]. This finding highlights the distinct and potentially opposing functions of innate immune cell subsets in the acute post-infarction phase and suggests that selective modulation of neutrophil activity may represent a potential strategy for reducing post-MI arrhythmic risk.

#### 2.4.6. Neutrophil Degranulation and Extracellular Vesicles

Multi-omics analyses have identified neutrophil degranulation-related signatures as potential indicators for CHD. Degranulation products may contribute to vascular remodeling and immune dysregulation of the vessel wall through exosome-mediated molecular communication, providing an additional mechanism linking between neutrophil activation and vascular immune remodeling [42]. These findings suggest that neutrophil-derived extracellular vesicles may serve as both candidate biomarkers and functional mediators of disease progression.

#### 2.4.7. Pharmacological Interventions Targeting Neutrophil-Mediated Damage

Pharmacological strategies aimed at limiting neutrophil-mediated tissue injury have shown potential therapeutic value. For example, nicorandil has been reported to attenuate acute kidney injury associated with neutrophil activation by modulating inflammatory and immune responses in the setting of cardiorenal injury [148]. Such findings suggest that neutrophil-directed therapies may provide organ-protective effects extending beyond the coronary vasculature.

In summary, studies over the past five years have further delineated the multifaceted roles of neutrophils across the spectrum of CHD. Integration of composite indicators such as NLR, NHR, and NAR with improved mechanistic understanding of neutrophil extracellular traps (NETs), degranulation, and EV-mediated signaling—neutrophil biology offers new entry points for individualized therapeutic approaches and risk stratification in CHD.

### 2.5. Eosinophils and Basophils

Although immunological research in CHD has predominantly focused on neutrophils and monocytes/macrophages, eosinophils and basophils—components of the granulocyte compartment of innate immunity—have attracted increasing attention for their potential roles in cardiovascular inflammation, plaque stability, and microvascular dysfunction. Emerging evidence suggests that these granulocyte subsets are involved in the pathophysiology of the pathogenesis of ACS but also may also have potential value as biomarkers for in-stent restenosis (ISR), coronary microvascular dysfunction, and disease differentiation.

#### 2.5.1. Eosinophils

##### Eosinophil Biology and Effector Molecules in CHD

Eosinophils contain a broad repertoire of preformed mediators—including cytokines such as IL-4, IL-5, and IL-13, chemokines, growth factors, eosinophil cationic protein (ECP), and eosinophil-derived neurotoxin (EDN)—that are can be released through multiple secretory pathways to contribute to intercellular signaling and modulate the local inflammatory microenvironment [149].

##### Dual and Conflicting Roles in Myocardial Infarction

The findings described in this subsection pertain specifically to Stage III (post-infarction myocardial repair) and reflect the wound-healing function of eosinophils in the infarcted myocardium. Eosinophil-derived IL-4 promotes reparative fibroblast activation and adaptive scar formation, a process that is mechanistically distinct from the potential role of eosinophils in atherosclerotic plaque inflammation (Stage I), which remains poorly characterized. The role of eosinophils in cardiovascular disease exhibits a dichotomous nature, with evidence supporting both cardioprotective and detrimental effects. A study integrating data from Danish cardiovascular screening cohorts with murine MI models demonstrated that eosinophil accumulation in the infarcted myocardium. eosinophil-derived mediators, including IL-4, ECP, and EDN may exert cardioprotective effects by limiting cardiomyocyte apoptosis, modulating fibroblast activity, and regulating post-MI inflammation and repair [45].

Conversely, contrasting clinical findings have emerged. In separate cohorts of patients undergoing coronary angiography, those with non-ST-elevation myocardial infarction (NSTEMI) and ST-elevation myocardial infarction (STEMI) exhibited significantly lower peripheral eosinophil counts compared with non-CHD controls [44]. This apparent discrepancy has been hypothesized to reflect the dynamic redistribution of eosinophils from the systemic circulation to the infarcted myocardium during acute ischemic events, which manifests as peripheral eosinopenia despite local tissue accumulation. However, it should be emphasized that this redistribution explanation remains an unvalidated hypothesis, as none of the cited studies simultaneously quantified peripheral blood eosinophil counts and myocardial tissue eosinophil infiltration in the same patients. Alternative explanations for the observed peripheral eosinopenia include stress-induced suppression of bone marrow eosinophil production, activation-induced apoptosis, splenic sequestration, and glucocorticoid-mediated eosinophil marginalization during the acute stress response.

##### ECP as a Biomarker for CHD Severity

Despite inconsistent findings regarding circulating eosinophil counts, markers of eosinophil activation, particularly eosinophil cationic protein (ECP), appear to show more consistent associations with CHD severity. Patients with angiographically confirmed CHD, including those with stable angina and non-ST-segment elevation myocardial infarction (NSTEMI), exhibited significantly higher circulating ECP levels than individuals with angiographically normal coronary arteries, supporting ECP as a potential surrogate marker of eosinophil activation in vivo [75]. Furthermore, patients who experienced major adverse cardiovascular events (MACE) had higher serum ECP levels than those who remained event free [150]. Collectively, these findings suggest that eosinophil activation status may provide clinically relevant information beyond absolute circulating eosinophil counts. However, prospective studies are needed to determine whether eosinophil-derived markers provide independent prognostic value and can be incorporated into clinically useful risk-stratification strategies [151].

##### Eosinophils in Post-PCI Prognosis and In-Stent Restenosis

Circulating eosinophil-related parameters have been investigated as potential prognostic biomarkers following percutaneous coronary intervention (PCI). Elevated baseline eosinophil counts have been associated with an increased risk of in-stent restenosis (ISR) after drug-eluting stent (DES) implantation, potentially reflecting enhanced eosinophil-associated inflammatory activity within the coronary vasculature [152]. During post-PCI follow-up, the monocyte-to-eosinophil ratio (MER) has also been associated with the development of de novo coronary lesions. Patients who developed de novo lesions exhibited lower baseline MER values than those without such lesions, suggesting that alterations in the relative balance between monocytes and eosinophils may be associated with vascular changes after coronary intervention [39]. Further studies are required to clarify the underlying biological mechanisms and to determine the incremental prognostic value of these eosinophil-related indices [90].

##### Eosinophils in Immune Infiltration Networks and Microvascular Dysfunction

Bioinformatics analyses using weighted gene co-expression network analysis (WGCNA) have identified an association between eosinophil infiltration and activated mast-cell signatures within the immune landscape of CHD [42]. These findings suggest that eosinophils may participate in broader innate immune networks rather than acting as isolated effector cells. However, associations derived from computational immune-infiltration analyses should not be interpreted as direct evidence of functional cellular interactions without further experimental validation.

Additional evidence for the potential involvement of eosinophils in coronary microvascular dysfunction comes from studies of the coronary slow-flow phenomenon (CSFP). Patients with CSFP have been reported to exhibit higher peripheral blood eosinophil counts, as well as elevated eosinophil-to-basophil ratio (EBR) and monocyte-to-basophil ratio (MBR), compared with control participants [153]. As inexpensive and readily available hematological indices, these parameters may have potential value in the clinical assessment of CSFP. Nevertheless, their diagnostic performance and incremental value over established clinical markers require further validation.

#### 2.5.2. Basophils

##### Basophils and Metabolic Risk Factors

Compared with eosinophils, evidence regarding the role of basophils in CHD remains relatively limited. A Japanese cohort study investigating associations between CHD risk factors and differential leukocyte counts found that higher basophil counts were associated with adverse lipid profiles, particularly hypertriglyceridemia [154]. The same study reported that, among patients with clinically stable CHD, elevated neutrophil and basophil counts were associated with increased risks of cardiovascular and all-cause mortality and with higher fibrinogen levels. These findings suggest a potential relationship between circulating basophil levels, systemic inflammation, and adverse cardiovascular outcomes, although the underlying mechanisms remain incompletely understood.

##### Basophil-Driven Reparative Fibrosis

Basophil-mediated reparative fibrosis, as described in this subsection, is a Stage III (post-infarction) phenomenon demonstrated in murine LAD artery ligation models. Basophil-derived signals that promote fibroblast activation and collagen deposition have a wound-healing function in the infarcted myocardium. This should not be conflated with any potential role of basophils in chronic atherogenesis (Stage I), for which the evidence is derived primarily from MR studies of peripheral basophil counts, a fundamentally different type of evidence that establishes genetic causal associations but does not directly demonstrate basophil infiltration or functional activity within atherosclerotic plaques. Despite their association with adverse metabolic profiles, basophils also contribute to beneficial post-infarction processes. Basophils promote healing and appropriate scarring of the infarcted heart by secreting IL-4 and IL-13, which facilitate the transition from acute inflammation to reparative fibrosis [155]. These findings further highlight the context-dependent functions of basophils in cardiovascular disease.

##### Genetic Evidence and Clinical Phenotype Associations

Recent Mendelian randomization analyses have suggested an inverse causal association between genetically predicted basophil counts and CHD risk, with an odds ratio (OR) of 0.92 reported in one analysis [156]. In clinical phenotyping, patients with early-onset coronary artery disease (EOCAD) and MI frequently exhibit significantly decreased basophil proportions. One hypothesized explanation is the redistribution of basophils from circulation into inflamed cardiac tissues; however, this remains speculative, as none of the available studies simultaneously measured peripheral blood basophil counts and tissue-level basophil infiltration in the same subjects. Other plausible mechanisms include activation-induced apoptosis, consumptive depletion through degranulation, cytokine-driven alterations in bone marrow output, and altered splenic sequestration [157].

##### Basophil Ratios in Differential Diagnosis and Severity Assessment

Basophil counts and basophil-derived ratios have also been explored as potential adjunctive biomarkers for differential diagnosis and disease severity assessment. For example, the basophil-to-lymphocyte ratio (BLR) has been reported to be higher in patients with Takotsubo syndrome (TTS) than in those with acute coronary syndrome (ACS), suggesting potential utility in distinguishing between these two conditions with overlapping clinical presentations [158]. In addition, basophil counts have been associated with the Gensini score, a measure of the angiographic extent and severity of coronary atherosclerosis, and may therefore provide additional information for evaluating coronary disease burden in patients with ACS [159].

#### 2.5.3. Summary and Future Directions

**Figure 2:**
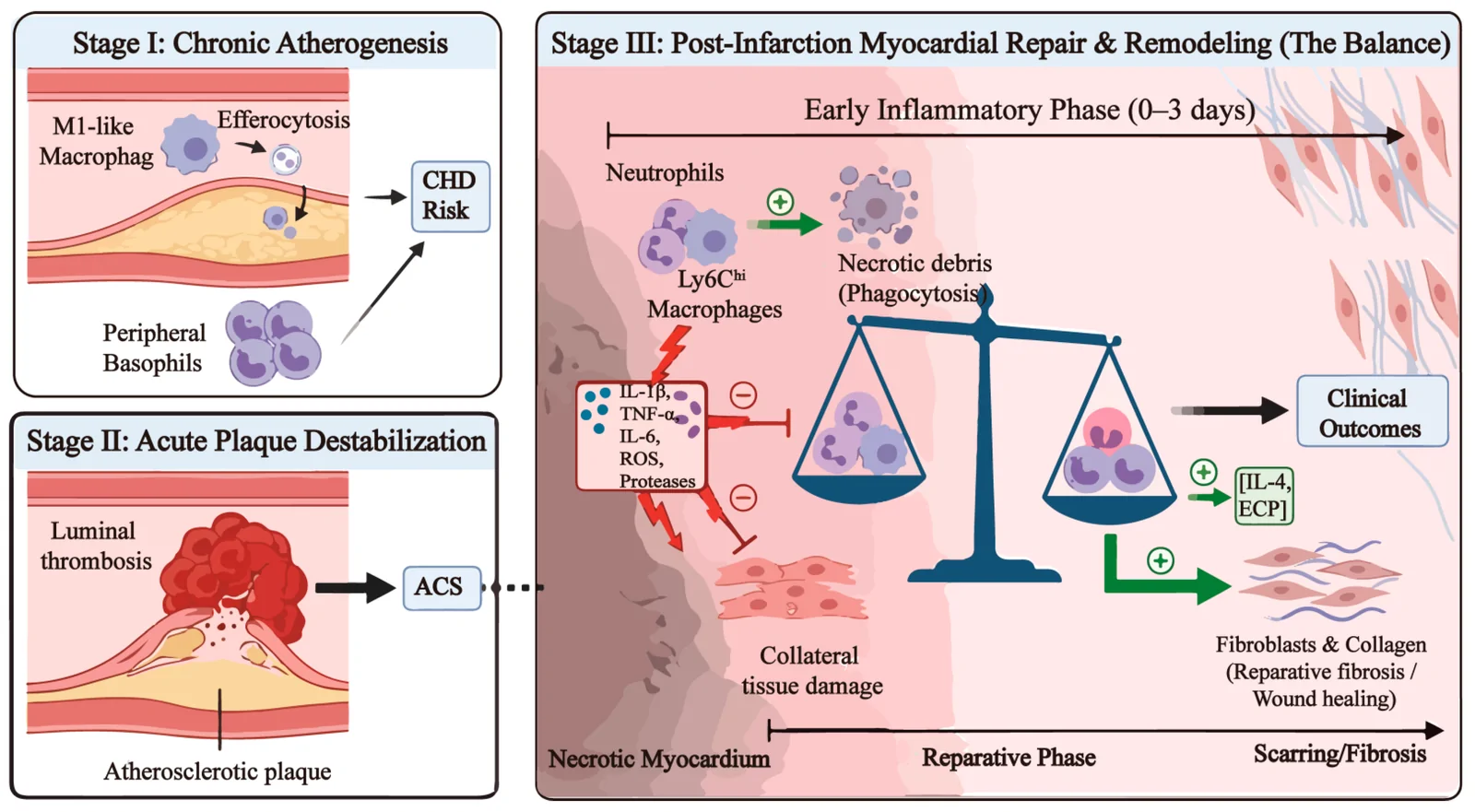
Dynamic evolution of immune responses across coronary heart disease stages and the inflammation–repair balance after myocardial infarction. The progression of coronary heart disease is accompanied by stage-dependent alterations in immune-cell activity and inflammatory responses. Stage I: Chronic atherogenesis. During long-term atherosclerotic progression, pro-inflammatory M1-like macrophage activity and impaired clearance of apoptotic or necrotic cells (efferocytosis) contribute to plaque development and progression, while alterations in circulating basophils are also associated with coronary heart disease (CHD) risk. Stage II: Acute plaque destabilization. With disease progression, plaque rupture or erosion followed by luminal thrombosis may precipitate acute coronary syndrome (ACS), marking the transition from chronic atherosclerosis to an acute ischemic event. Stage III: Post-infarction myocardial repair and remodeling. During the early inflammatory phase (0–3 days), neutrophils and Ly6C^hi^ inflammatory macrophages accumulate in the infarcted myocardium and participate in the phagocytic clearance of necrotic debris. These cells also release inflammatory mediators, including IL-1β, TNF-α, IL-6, reactive oxygen species (ROS), and proteases; excessive or prolonged inflammation may result in collateral tissue injury. This is followed by a reparative phase characterized by progressive resolution of inflammation and activation of repair-associated immune pathways. Eosinophil- and basophil-associated responses, together with mediators such as IL-4 and eosinophil cationic protein (ECP), contribute to the regulation of tissue repair and promote fibroblast activation, collagen deposition, and reparative fibrosis/scar formation. The dynamic balance between inflammatory clearance and reparative responses ultimately influences ventricular remodeling and clinical outcomes after myocardial infarction. Different arrow types represent distinct biological interactions: green arrows denote activating, promoting, or reparative effects, whereas red arrows denote inhibitory, pathological, or pro-inflammatory effects. Abbreviations: ACS, acute coronary syndrome; CHD, coronary heart disease; ECP, eosinophil cationic protein; IL, interleukin; ROS, reactive oxygen species; TNF-α, tumor necrosis factor-α.

Recent studies have expanded our understanding of the potential roles of eosinophils and basophils in CHD, ranging from genetic associations and disease severity assessment to post-PCI outcomes, microvascular dysfunction, and differential diagnosis. Eosinophil-related markers such as ECP, circulating eosinophil counts, and MER, together with basophil-related measures such as BLR, may provide complementary information for cardiovascular risk stratification in selected clinical settings. Composite indices integrating granulocyte subsets with lymphocyte or monocyte counts may further capture aspects of systemic immune balance that are not reflected by individual leukocyte counts alone. The dynamic interplay between macrophage-mediated clearance and granulocyte-driven repair during these stages is summarized in Figure 2.

### 2.6. Natural Killer Cells

#### 2.6.1. Overview: From Innate Immune Effectors to Regulators of Vascular Pathology

Natural killer (NK) cells are innate lymphoid cells that express a diverse repertoire of activating and inhibitory receptors, enabling them to sense cellular stress and pathological alterations. Upon activation, NK cells secrete cytokines such as interferon-γ (IFN-γ) and release cytotoxic molecules, including perforin and granzymes, thereby mediating potent cytotoxic and immunoregulatory responses [160]. Recent prospective cohort studies, single-cell multi-omics analyses, and genetic investigations have expanded our understanding of the involvement of NK cells in inflammation and vascular pathology across different stages of coronary heart disease (CHD), ranging from subclinical coronary artery calcification (CAC) to acute coronary syndrome (ACS). Rather than functioning solely as innate cytotoxic effectors, NK cells appear to exert context-dependent immunoregulatory effects during the development and progression of coronary atherosclerosis.

#### 2.6.2. NK Cells in Subclinical Coronary Artery Calcification

At the subclinical stage of coronary atherosclerosis, circulating NK cell profiles have been associated with the development of CAC. Data from the Multi-Ethnic Study of Atherosclerosis (MESA) showed that a higher baseline NK cell frequency was independently associated with a greater risk of incident CAC during long-term follow-up (HR = 1.26 per SD increase) [161]. These findings suggest that NK cell-related immune activity may be involved in the early stages of coronary atherosclerosis, although the observational nature of the association does not establish a direct pathogenic role. Genetic analyses have provided additional evidence linking NK cell phenotypes, lipid metabolism, and coronary atherosclerosis. Mendelian randomization (MR) analyses identified genetically predicted HLA-DR-positive (HLA-DR^+^) NK cell traits as being associated with CHD or CAC in the context of lipid-related traits, including diacylglycerol and triglyceride levels [162]. These findings support a potential interaction between metabolic dysregulation and NK cell-related immune pathways in atherogenesis. However, they should be interpreted cautiously, as MR findings support causal inference at the level of genetically proxied traits but do not by themselves establish that NK cells directly mediate lipid-induced vascular injury.

#### 2.6.3. Phenotypic Heterogeneity Between Circulation and Plaque Tissue

NK cells exhibit marked phenotypic and functional heterogeneity across anatomical compartments. Cellular Indexing of Transcriptomes and Epitopes by Sequencing (CITE-seq) analyses revealed that SPON2 expression in peripheral blood NK cells was positively associated with the severity of coronary artery stenosis, suggesting its potential value as a marker of disease burden. In contrast, SPON2 expression was lower in NK cells within pro-inflammatory atherosclerotic plaques, and this compartment-specific expression pattern was influenced by human cytomegalovirus (HCMV) infection status and comorbid conditions such as diabetes [163]. These contrasting findings indicate that circulating and plaque-resident NK cells may exhibit distinct transcriptional and functional states. Rather than demonstrating a definitive transition from a “pro-homeostatic” circulating phenotype to a “pro-inflammatory” plaque phenotype, the available evidence supports substantial compartment-dependent heterogeneity. This observation underscores the importance of examining NK cells within their local tissue microenvironment rather than inferring plaque-level biology solely from peripheral blood measurements.

#### 2.6.4. Chemokine-Mediated Recruitment and Dual Functional Roles

The recruitment and activation of NK cells within atherosclerotic lesions are regulated by multiple chemokines and cytokines. Chemotactic mediators such as monocyte chemoattractant protein-1 (MCP-1/CCL2) and CX3CL1 (fractalkine), together with cytokines including interleukin-15 (IL-15), IL-12, and IL-18, can influence NK cell trafficking, activation, survival, and effector functions [164,165]. In activated NK cells, these signals may promote the production of IFN-γ, which can amplify local inflammatory responses and potentially contribute to atherogenesis [166]. These observations illustrate the context-dependent functions of NK cells in cardiovascular disease: NK cell activation may contribute to inflammatory processes during chronic atherogenesis, whereas specific NK cell subsets or functional states may support tissue protection and repair following acute myocardial injury.

#### 2.6.5. Clinical Evidence: NK Cell Depletion in CHD Patients

Clinical studies have reported alterations in circulating NK cell abundance in patients with established CHD. In a cohort of 190 participants, including 98 patients with CHD and 92 healthy controls, patients with CHD exhibited an approximately 15% lower circulating NK cell frequency. Reduced NK cell frequencies were observed across several clinical presentations of CHD, including unstable angina (UA), non-ST-segment elevation myocardial infarction (NSTEMI), and ST-segment elevation myocardial infarction (STEMI) [167]. Circulating NK cell abundance has also been reported to be inversely associated with the angiographic severity of coronary stenosis. In a study of 150 patients stratified according to stenosis severity, NK cell counts were lower in patients with coronary stenosis than in those without angiographically detectable stenosis, with reduced levels observed across groups with different degrees of stenosis [168]. This inverse relationship between NK cell number and disease severity has been hypothesized to reflect the increased recruitment and consumption of NK cells at sites of active vascular inflammation, mirroring the peripheral depletion observed in eosinophils during acute coronary events (see Section Dual and Conflicting Roles in Myocardial Infarction). However, this redistribution hypothesis has not been directly validated, as no study to date has simultaneously quantified circulating NK cell counts and tissue-resident NK cell density in the same patient at the same time point. Alternative mechanisms that may account for the observed peripheral NK cell cytopenia include catecholamine-mediated alterations in NK cell margination dynamics, activation-induced cell death following exposure to circulating DAMPs, altered splenic sequestration, and reduced bone marrow output under acute inflammatory stress.

#### 2.6.6. Environmental Exposures and NK Cell-Mediated Immune Responses

Emerging studies have explored potential links between environmental exposures, NK cell-related immune responses, and cardiovascular disease. Blood microplastic concentrations have been reported to be higher in patients with ACS, and microplastic burden has been positively associated with circulating NK cell counts and levels of inflammatory mediators such as IL-6 and IL-12p70 [99]. These observations raise the possibility that microplastic exposure may be associated with immune-inflammatory activation in ACS.

However, the available observational evidence does not establish that microplastics directly trigger NK cell-mediated vascular injury or acute cardiovascular events. Further mechanistic and prospective studies are required to determine whether NK cells participate causally in the cardiovascular effects associated with microplastic exposure and to clarify the relevant cellular and molecular pathways.

### 2.7. Dendritic Cells

#### 2.7.1. Overview: The Bridge Between Innate and Adaptive Immunity in CHD

Dendritic cells (DCs) are highly specialized professional antigen-presenting cells that serve as an important bridge between innate and adaptive immunity. They play central roles in antigen sensing and processing, T-cell activation and polarization, regulation of inflammatory responses, and maintenance of immune tolerance [169,170]. Given that atherosclerosis, the principal pathological substrate of coronary heart disease (CHD), is characterized by chronic lipid accumulation and inflammation, increasing attention has been directed toward the involvement of DCs in lipid and antigen uptake, clearance of dying cells, sensing of damage-associated molecular patterns (DAMPs), and modulation of the local immune microenvironment. Recent studies using clinical cohorts, high-dimensional immune profiling, transcriptomic analyses, genetic approaches, and experimental models have implicated DCs in multiple stages of CHD pathogenesis, including plaque formation and destabilization, systemic immune dysregulation, inflammatory signaling, and interactions with adaptive immune responses. These findings have also raised interest in DC-associated pathways as potential therapeutic targets.

#### 2.7.2. DC Subtypes and Functional Biology

DCs comprise several phenotypically and functionally distinct populations, among which conventional dendritic cells (cDCs) and plasmacytoid dendritic cells (pDCs) represent two major lineages [171]. Conventional DCs are further divided into cDC1 and cDC2 subsets, whereas monocyte-derived dendritic cells (MoDCs) arise predominantly under inflammatory conditions and are generally considered distinct from the canonical cDC lineage [172]. These populations perform partially overlapping but specialized functions. cDC1 cells are particularly efficient at cross-presentation of exogenous antigens to CD8^+^ T cells, whereas cDC2 cells are more closely associated with the activation and polarization of CD4^+^ T-cell responses. pDCs are characterized by their capacity to produce large amounts of type I interferons, particularly in response to nucleic acid sensing. MoDCs exhibit features of both dendritic cells and monocyte/macrophage-lineage cells and can acquire potent antigen-presenting and inflammatory functions under appropriate conditions [61,173].

During maturation, DCs increase the expression of major histocompatibility complex class II (MHC-II) molecules and co-stimulatory molecules such as CD80 and CD86, thereby enhancing their capacity to activate T cells. Cytokines and growth factors, including granulocyte-macrophage colony-stimulating factor (GM-CSF), can influence DC differentiation, survival, maturation, and functional state depending on the cellular and inflammatory context [174]. Activated DCs also produce a diverse range of cytokines, including IL-12 and IL-10, which can shape downstream immune responses [175]. Through antigen presentation, co-stimulatory signaling, and cytokine production, DCs contribute to the differentiation and functional polarization of naïve CD4^+^ T cells toward distinct effector or regulatory states, including Th1, Th2, Th17, and regulatory T-cell (Treg) responses [176]. These T-cell programs may have divergent effects on atherosclerotic inflammation. Accordingly, DCs occupy an important regulatory position at the interface between innate immune sensing and adaptive immune activation. Their ability to integrate local danger signals and subsequently shape T-cell responses provides a mechanistic link between the innate immune pathways discussed in Section 2.2, Section 2.3, Section 2.4, Section 2.5 and Section 2.6 and the adaptive immune mechanisms described in Section 3.2 and Section 3.3.

#### 2.7.3. Early Evidence: DCs in Myocardial Infarction and Cardiac Injury

Early experimental studies provided evidence that DCs participate in the immune response to myocardial injury. Increased numbers of DCs were observed within infarcted myocardial tissue in rat models of myocardial infarction (MI) [177]. Experimental studies of cardiac injury have further demonstrated that interactions among antigen-presenting cells, macrophages, and other immune populations contribute to post-injury inflammation and tissue repair. In murine models of left ventricular cryoinjury, macrophage depletion was associated with increased mortality, left ventricular dilation, wall thinning, impaired wound healing, and adverse cardiac remodeling [178]. Although these findings do not isolate a DC-specific causal effect, they provided early evidence that the antigen-presenting-cell network and its interactions with other myeloid populations actively participate in the response to myocardial injury rather than simply representing passive inflammatory infiltration. Subsequent studies have further supported context-dependent roles for DCs in myocardial inflammation, repair, and remodeling.

#### 2.7.4. Clinical Evidence: DC Alterations in CHD Patients

Accumulating evidence implicates DCs in the initiation and regulation of immune responses associated with atherosclerosis [179,180,181,182]. Atherosclerotic inflammation involves coordinated chemokine and cytokine signaling, expression of regulatory and co-stimulatory molecules, antigen presentation, and interactions between innate and adaptive immune cells [183,184,185]. Consistent with this framework, clinical studies have reported alterations in circulating DC abundance and activation phenotypes in patients with CHD.

One cohort study reported higher peripheral blood DC frequencies in patients with CHD than in individuals with normal coronary angiography, together with findings consistent with enhanced Th1-associated immune responses [186]. In another study, DCs from patients with CHD exhibited higher expression of the co-stimulatory molecule CD86 than those from healthy controls, suggesting an enhanced activation or co-stimulatory phenotype. CD86 expression was significantly associated with C-reactive protein levels but not with LDL cholesterol in that cohort. Rather than demonstrating that DC activation is independent of lipid metabolism, these findings suggest a closer association between the measured DC activation phenotype and systemic inflammatory burden than with circulating LDL cholesterol at the time of assessment [187]. High-dimensional immunophenotyping has provided additional evidence of immune-system remodeling in CHD. Kott et al. (2023), using mass cytometry to characterize circulating immune-cell populations in patients with CHD, identified alterations across multiple immune compartments, including monocytes, neutrophils, T cells, and antigen-presenting-cell populations such as DCs [63]. Collectively, these observations support the concept that CHD is associated with coordinated remodeling of a multicellular immune network rather than abnormalities in a single immune-cell population. Within this network, DCs may contribute to the integration of innate inflammatory signals with adaptive immune activation.

#### 2.7.5. The DAMPs–DC Axis: Danger Signal Transduction and Immune Amplification

A potentially important mechanism linking tissue injury to immune activation in CHD involves DAMP sensing by DCs. Atherosclerotic plaques contain accumulated lipids, dying and necrotic cells, extracellular matrix fragments, and products of oxidative stress, all of which may contribute to the generation or release of endogenous danger signals. DAMPs implicated in atherosclerotic inflammation include high-mobility group box 1 (HMGB1), members of the S100 protein family, and heat shock proteins (HSPs).

These signals can be sensed through pattern-recognition receptors (PRRs) expressed at the cell surface, within endosomal compartments, or in the cytosol of DCs and other innate immune cells. Depending on the ligand, receptor, and cellular context, DAMP sensing can promote DC activation or maturation, alter cytokine production, influence migration, and modify their capacity to activate T cells. Through these mechanisms, DCs may contribute to the propagation of local inflammatory responses and communication between innate and adaptive immunity.

Using transcriptomic and single-cell analytical approaches, Li et al. (2025) investigated DAMP-related immune signatures in CHD and reported alterations in DAMP-associated signaling together with changes in immune-cell populations, including DC-associated signatures [188]. These findings support the potential involvement of DCs in DAMP-related immune responses in CHD. However, transcriptomic and computational associations alone do not establish that DCs are the principal cellular mediators of DAMP signaling or that a specific DAMP–DC pathway directly drives plaque progression.

Therefore, the DAMP–DC axis is best regarded as a plausible mechanistic framework through which tissue damage may influence antigen-presenting-cell function and downstream adaptive immunity. Further spatial and functional studies are required to define the relative contributions of individual DAMPs, PRRs, and DC subsets within human coronary plaques.

#### 2.7.6. Necroptosis-Related Signals and DC Activation

Programmed inflammatory cell-death pathways may provide an additional source of endogenous signals capable of influencing DC function. Chen et al. (2024) analyzed necroptosis-related gene-expression patterns in CHD and identified TLR3, MLKL, HMGB1, and NDRG2 as candidate diagnostic markers, together with alterations in inferred immune-cell infiltration [35]. These findings establish associations between necroptosis-related molecular signatures and CHD but do not by themselves demonstrate active necroptosis or direct activation of DCs.

Nevertheless, several of the identified molecules have biological relevance to DC function. TLR3 is an endosomal pattern-recognition receptor that recognizes double-stranded RNA and can activate inflammatory and antiviral signaling pathways in TLR3-expressing DC subsets. HMGB1, which may be released or exposed during cellular stress, injury, or death, can act as an extracellular danger signal and modulate DC maturation, cytokine production, antigen-presenting capacity, and co-stimulatory molecule expression in a context-dependent manner.

These observations provide a mechanistic rationale for a potential connection between inflammatory cell death, DAMP release, and DC-associated immune responses in CHD. However, direct evidence demonstrating a sequential necroptosis–DAMP–DC pathway in human coronary disease remains limited. Accordingly, DCs should be considered potential participants in, rather than definitively established central mediators of, this pathological cascade.

#### 2.7.7. Genetic Causal Evidence: Mendelian Randomization Studies

Mendelian randomization (MR) has been employed to evaluate potential causal relationships between immunophenotypes and cardiovascular diseases. Wang et al. (2024), using MR to assess the causal relationships between genetically proxied immunophenotypes and cardiovascular disease, revealed that multiple immune cell signatures—including DC-associated immune signatures—may be causally linked to cardiovascular disease risk [189]. These findings imply that DC number, maturation status, or surface molecular expression characteristics may not simply reflect disease consequences but may be related to disease susceptibility itself. For CHD, this suggests that DCs may not only drive the inflammatory evolution of established plaques but may also play a predisposing role in establishing an individual’s underlying genetic susceptibility to coronary inflammation.

#### 2.7.8. Therapeutic Targeting of DCs: Dimethyl Fumarate as Proof of Concept

At the therapeutic level, Sun et al. (2022) reported that dimethyl fumarate (DMF) can significantly inhibit the maturation and migration of dendritic cells, proposing DMF as a potential protective agent for CHD [190]. This study carries clear translational implications. In coronary atherosclerosis, DCs initiate adaptive immune responses by migrating to lymphoid organs following antigen uptake. Inhibiting DC maturation and migration may attenuate the amplification of T-cell-mediated chronic inflammation, thereby alleviating immune-mediated vascular damage. DMF, which possesses both immunomodulatory and antioxidant properties and is already approved for other immune-inflammatory diseases, represents a proof of concept that DC function in CHD is modifiable and therapeutically targetable [5]. This finding provides further evidence that the role of DCs in CHD is functionally relevant rather than merely an epiphenomenon of the disease.

#### 2.7.9. Summary, Limitations, and Future Directions

Research over the past five years has established DCs as indispensable components of the CHD immunopathological network. Current evidence demonstrates the following: (i) CHD patients exhibit abnormalities in DC-related immune profiles; (ii) DAMPs and necroptosis-related molecules may mediate inflammatory amplification through DC activation (iii) genetic immunophenotypic studies support a potential causal link between DC characteristics and cardiovascular disease risk [189]; and (iv) pharmacological inhibition of DC maturation and migration by agents such as DMF suggests that DCs may represent a novel target for immunotherapy in CHD [190]. However, several limitations persist in this field. First, regarding subset resolution, many studies treat DCs as a single category in immune infiltration analyses, lacking detailed differentiation of cDC1, cDC2, pDC, and inflammatory DC subsets. Second, regarding the evidence base, most evidence derives from peripheral blood transcriptomics, bioinformatics inference, or genetic association analyses. Functional studies directly examining DCs within coronary plaques or ischemic myocardial tissue remain relatively limited. Third, regarding bidirectional functionality, DCs may exert opposing effects at different disease stages—promoting inflammation in some contexts while maintaining immune tolerance and facilitating tissue repair in others. Therefore, categorically defining DCs merely as “pro-atherogenic” cells oversimplifies their complex biology. Future research should integrate single-cell transcriptomics, spatial omics, plaque histology, and in vivo functional experiments to clarify the precise roles of different DC subsets at different stages of CHD.

### 2.8. Mast Cells

#### 2.8.1. Overview: Beyond Allergy to Cardiovascular Immunopathology

Mast cells (MCs) are tissue-resident effector cells of the innate immune system that are widely distributed in perivascular tissues and at mucosal barrier sites. Upon activation, they can rapidly release preformed mediators, including histamine and proteases, as well as newly synthesized cytokines, chemokines, and lipid mediators. Through these mediators, MCs regulate vascular permeability, leukocyte recruitment, inflammatory responses, and tissue remodeling. Although MCs have traditionally been recognized for their central role in allergic and hypersensitivity reactions, accumulating evidence has implicated them in a broader spectrum of cardiovascular inflammatory disorders, including atherosclerosis and coronary heart disease (CHD). Their potential contributions span chronic vascular inflammation, plaque progression and destabilization, coronary vasomotor abnormalities, and allergy-associated acute coronary syndromes (ACS).

#### 2.8.2. Causal Evidence and Biomarker Discovery

Recent integration of proteome-wide Mendelian randomization with multi-omics analyses has provided genetic evidence linking mast cell-associated molecular signatures to atherosclerotic cardiovascular disease. In particular, CALB2 (calbindin 2) was identified as a circulating protein with a genetically supported association with atherosclerosis and coronary artery disease. Single-cell transcriptomic analyses further demonstrated that CALB2 is specifically enriched in mast cells within atherosclerotic plaques and adipose tissue. In addition, plasma CALB2 levels were significantly elevated in patients with moderate-to-severe carotid artery stenosis (>50%) compared with those with <50% stenosis, suggesting that CALB2 may represent a candidate biomarker and potential therapeutic target [191]. Bioinformatics-based immune infiltration analyses have also revealed altered mast cell-associated signatures in CHD. Fu et al. reported increased mast cell and neutrophil infiltration signatures in patients with CHD, while activated mast cells were positively correlated with eosinophil infiltration [103]. In addition, necroptosis-related transcriptomic analyses have identified alterations in mast cell-associated immune infiltration patterns in CHD. However, mast cells were not the principal biomarkers used to construct the necroptosis-related diagnostic model; rather, the model was based on hub genes including TLR3, MLKL, HMGB1, and NDRG2. Therefore, these findings support an association between mast cell-related immune profiles and CHD molecular phenotypes but do not yet establish mast cells themselves as validated diagnostic biomarkers.

#### 2.8.3. Kounis Syndrome: Mast Cells as Mediators of Allergic Acute Coronary Syndrome

Kounis syndrome (KS) represents a clinically important intersection between allergic hypersensitivity and acute coronary disease. It is characterized by the occurrence of ACS in the setting of an allergic or anaphylactic reaction, with mast cell activation and mediator release playing central pathogenic roles. Histamine, tryptase, leukotrienes, platelet-activating factor, and other inflammatory mediators can induce coronary vasoconstriction, endothelial dysfunction, platelet activation, and potentially plaque destabilization. Clinical manifestations of KS include allergic coronary vasospasm, acute myocardial infarction associated with pre-existing or newly destabilized atherosclerotic disease, and allergic stent thrombosis. Case reports have described ST-segment elevation myocardial infarction following allergic reactions triggered by agents or exposures such as aspirin, fish, and rifampicin, with mast cell-mediated hypersensitivity proposed as an underlying mechanism [192]. Kounis syndrome therefore provides a clinically relevant example of how mast cell-mediated hypersensitivity can interact with coronary vascular pathology. However, because much of the literature consists of case reports and small case series, the incidence, risk factors, and optimal management of KS remain incompletely defined.

#### 2.8.4. Synergy with Lipid Metabolism: The ox-LDL–Histamine–H1R–HMGB1 Axis

Experimental evidence suggests that dysregulated lipid metabolism may modify mast cell-dependent allergic and vascular responses. In experimental hyperlipidemic settings, oxidized low-density lipoprotein (ox-LDL) has been reported to enhance histamine release from mast cells. Histamine may subsequently promote high-mobility group box 1 (HMGB1) release from vascular endothelial cells through signaling involving the histamine H1 receptor (H1R), thereby increasing endothelial permeability and amplifying inflammatory vascular injury [193]. These findings suggest a potential ox-LDL–histamine–H1R–HMGB1 signaling axis linking lipid-related stress, mast cell activation, and endothelial inflammatory responses. Importantly, however, the available evidence appears to arise primarily from experimental models of hyperlipidemia and allergic or anaphylactic injury. Therefore, this pathway should not yet be regarded as an established mechanism of CHD progression in humans. HMGB1 may nevertheless represent a mechanistically relevant downstream mediator because of its ability to promote inflammatory signaling and endothelial dysfunction. Further studies are required to determine whether targeting this pathway can provide therapeutic benefit in atherosclerosis, CHD, or allergy-associated cardiovascular injury.

#### 2.8.5. Classical Mechanisms: Mast Cells in Plaque Initiation and Foam Cell Formation

Histopathological studies have demonstrated the presence and accumulation of mast cells in the intima and adventitia of human arteries during atherosclerotic lesion development and progression [194,195,196]. Their localization within plaque-prone regions provides anatomical support for a potential role in local vascular inflammation. Experimental studies have also suggested that mast cells can facilitate macrophage lipid uptake. Mast cell granule components, including heparin-containing proteoglycans, can interact with LDL particles and promote the formation or modification of lipoprotein complexes that are subsequently taken up by macrophages, thereby facilitating foam cell formation under experimental conditions [197,198]. These findings provide a potential mechanistic link between mast cell degranulation and lipid accumulation within the plaque microenvironment. Chemokine signaling may also contribute to mast cell recruitment into atherosclerotic lesions. Eotaxin-1 (CCL11) and its receptor CCR3 have been implicated in mast cell trafficking and accumulation within atherosclerotic tissue [199]. In ApoE^−/−^ mouse models, pharmacological interference with CCR3 signaling has been reported to reduce mast cell accumulation at lesion sites and attenuate atherosclerotic progression [200].However, because CCR3 is also expressed by other immune-cell populations, including eosinophils, these experiments do not necessarily demonstrate that the observed vascular effects are mediated exclusively through mast cells.

#### 2.8.6. Translational Caveat: Species Differences in Mast Cell Distribution

An important consideration when interpreting experimental data is the species-specific difference in mast cell distribution. In mice, mast cells are predominantly found in the perivascular and adventitial tissues and are rare within the intima, whereas in human coronary atherosclerosis, activated mast cells accumulate in both the intima, particularly at the plaque shoulder [201], and adventitia. These species-specific differences limit the direct translational applicability of murine mast cell studies [202]. This limitation does not invalidate animal studies but underscores the importance of validating mechanistic findings in human atherosclerotic tissue. Future research would benefit from combining conventional animal models with human plaque histology, ex vivo vascular systems, and, where appropriate, humanized experimental models.

#### 2.8.7. Summary, Current Limitations, and Future Directions

Research over the past five years has consolidated the multi-level role of mast cells in CHD. In the chronic stage, mast cells promote coronary artery disease progression through immune infiltration, inflammatory amplification, and remodeling of the plaque microenvironment [203]. Mechanistically, the ox-LDL–histamine–H1R–HMGB1 axis demonstrates synergistic action among mast cells, lipid toxicity, and endothelial injury [35]. At the translational level, mast cell-related molecules such as CALB2 represent promising new biomarkers of atherosclerosis [42]. In the acute clinical setting, Kounis syndrome highlights the capacity of mast cells to directly induce coronary vasospasm, plaque instability, and stent thrombosis [192]. However, several important limitations remain in this field. First, regarding the evidence base, research on mast cells in CHD relies predominantly on bioinformatics analysis, case reports, and indirect evidence; direct plaque histology and functional experiments remain relatively scarce. Second, regarding phenotypic characterization, descriptions of mast cell “increase” or “decrease” across studies may be confounded by incomplete differentiation between resting and activated states, heterogeneous detection platforms, and variable sample sources. Third, regarding peripheral versus local assessment, peripheral blood measurements may not accurately reflect local mast cell activity within coronary arteries.

Studies integrating spatial transcriptomics, single-cell sequencing, and histopathology are needed to clarify the true role of mast cells in the coronary plaque microenvironment. In summary, research over the past five years has established mast cells as multifaceted regulators of CHD pathogenesis, operating across the disease spectrum from subclinical calcification to acute coronary events. The integration of genetic causal evidence (MR studies), bioinformatics profiling, and clinical case reports has revealed mast cells as a convergence point for lipid toxicity, immune hypersensitivity, and vascular injury.

## 3. Adaptive Immune Cells in CHD

### 3.1. Antigen-Specific Adaptive Immunity in Atherosclerosis: Mechanistic Foundation

**Figure 3:**
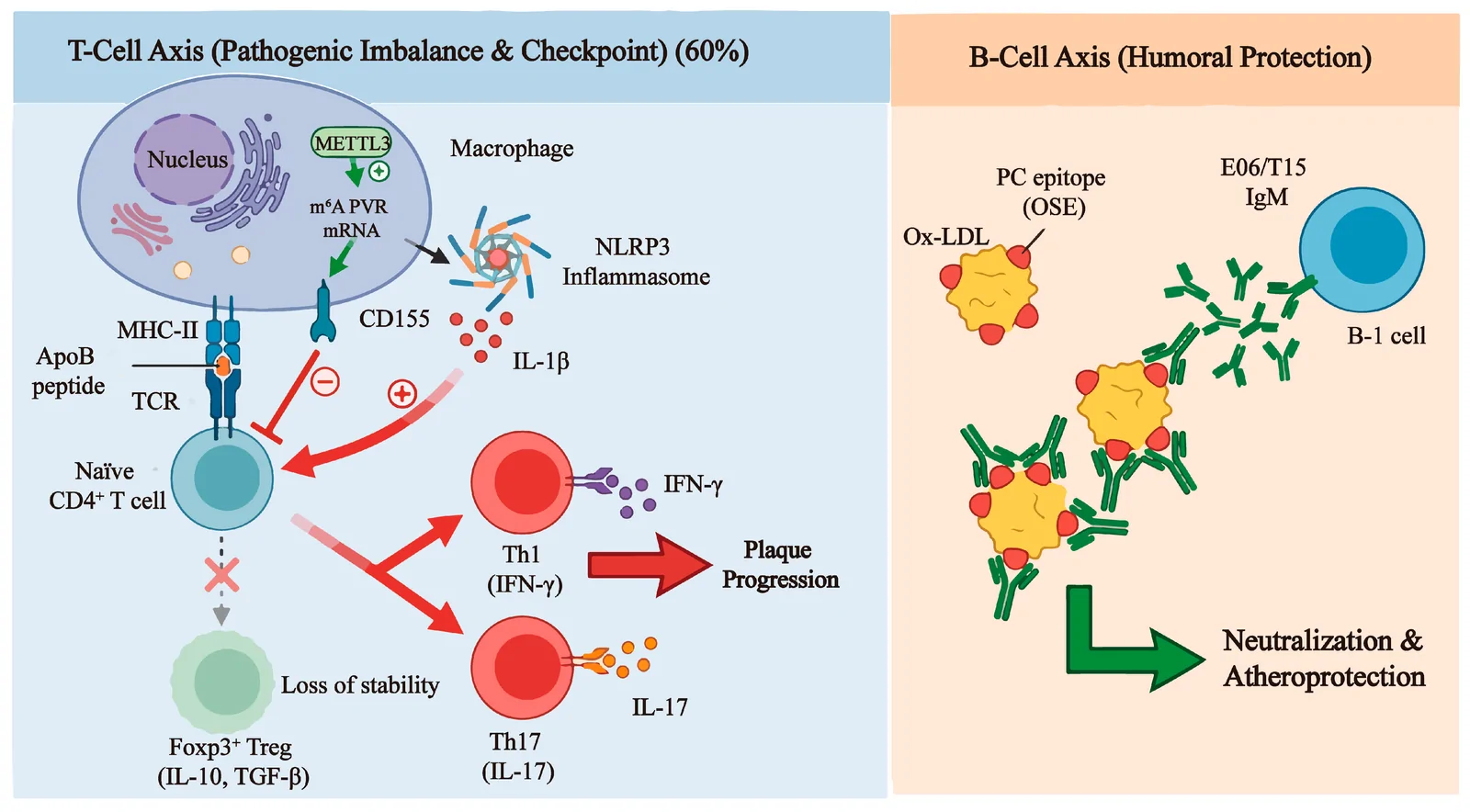
Schematic illustration of pathogenic T-cell imbalance, immune checkpoint regulation, and B-cell-mediated humoral protection during atherosclerotic progression in coronary heart disease. The left panel depicts the pro-atherogenic T-cell axis. ApoB-derived peptides are presented by MHC class II molecules and recognized by the T-cell receptor (TCR), promoting the activation and differentiation of naïve CD4^+^ T cells. Under conditions of immune dysregulation, CD4^+^ T-cell responses shift toward pro-inflammatory Th1 and Th17 phenotypes, which produce IFN-γ and IL-17, respectively, thereby amplifying vascular inflammation and promoting plaque progression. In parallel, loss of stability of Foxp3^+^ regulatory T cells (Tregs) weakens protective IL-10- and TGF-β-mediated immunoregulation. Macrophage-associated immune checkpoint signaling further modulates this response: METTL3-dependent m^6^A RNA modification regulates PVR/CD155-associated expression, whereas CD155-mediated checkpoint signaling influences T-cell activation. Concurrent activation of the NLRP3 inflammasome and release of IL-1β further amplify inflammatory signaling, collectively favoring pathogenic T-cell responses and plaque progression. The right panel illustrates the protective B-cell humoral axis. B-1 cells produce natural E06/T15-like IgM antibodies that recognize phosphocholine (PC) epitopes on oxidized low-density lipoprotein (ox-LDL). By neutralizing oxidation-associated lipid antigens and limiting their pro-inflammatory effects, these antibodies contribute to atheroprotection. Overall, the figure highlights the functional balance between pathogenic T-cell immunity and protective B-cell-mediated humoral responses in determining the trajectory of atherosclerotic disease. Different arrow types represent distinct biological interactions: solid arrows indicate direct regulation, while dashed arrows denote indirect or regulatory crosstalk. Green arrows denote activating, promoting, or reparative effects, whereas red arrows denote inhibitory, pathological, or pro-inflammatory effects. Abbreviations: ApoB, apolipoprotein B; IFN-γ, interferon-γ; IL, interleukin; METTL3, methyltransferase-like 3; MHC-II, major histocompatibility complex class II; NLRP3, NOD-like receptor family pyrin domain-containing 3; ox-LDL, oxidized low-density lipoprotein; PC, phosphocholine; PVR/CD155, poliovirus receptor/CD155; TCR, T-cell receptor; TGF-β, transforming growth factor-β; Th, T helper cell; Treg, regulatory T cell.

Before examining the roles of individual adaptive immune cell subsets in CHD (Section 3.2, Section 3.3 and Section 3.4), it is essential to establish the antigen-specific basis of adaptive immune responses in atherosclerosis. Unlike innate immune cells, which recognize broad pathogens or DAMPs, T and B lymphocytes mediate antigen-specific responses via clonally distributed receptors (T-cell receptor [TCR] and B-cell receptor [BCR]) (Figure 3). A growing body of evidence demonstrates that atherosclerosis involves bona fide antigen-driven adaptive immune responses directed against defined and modified self-antigens, rather than merely nonspecific immune activation [204].

#### 3.1.1. ApoB-Reactive CD4^+^ T Cells and Phenotypic Plasticity

Apolipoprotein B-100 (ApoB), the principal protein component of LDL and its oxidized derivatives, is a major CD4^+^ T-cell autoantigen in atherosclerosis. Using MHC II tetramers loaded with ApoB-derived peptide epitopes, Kimura et al. demonstrated the presence of ApoB-reactive CD4^+^ T cells in both the peripheral blood and atherosclerotic plaques of human subjects [205].Critically, these antigen-specific T cells displayed distinct phenotypic profiles depending on disease status: in healthy individuals, ApoB-reactive CD4^+^ T cells predominantly exhibited a Foxp3^+^ Treg phenotype, whereas in patients with cardiovascular disease, a significant proportion had undergone phenotypic drift toward T-bet^+^ IFN-γ^+^ Th1 and RORγt^+^ IL-17^+^ Th17 effector states [205,206]. Wolf et al. further demonstrated that this Treg-to-effector transition is associated with loss of suppressive function and acquisition of pro-inflammatory cytokine production, directly linking antigen-specific autoreactive T-cell plasticity to plaque progression [206]. These findings provide a critical mechanistic anchor for the Th17/Treg imbalance observed in patients with CHD (discussed in Section 3.2.3): the shift in bulk Th17/Treg ratios reported in clinical studies reflects, at least in part, the phenotypic conversion of ApoB-specific Tregs into pro-atherogenic effector cells, rather than merely a nonspecific perturbation of immune homeostasis [207].

#### 3.1.2. Oxidation-Specific Epitopes and B-Cell Antigen Specificity

On the humoral side, OSEs are the dominant B-cell antigenic targets in atherosclerosis. OSEs are generated during oxidative modification of LDL and cellular membrane phospholipids, including phosphorylcholine (PC) head groups on oxidized phospholipids (OxPLs), malondialdehyde–lysine (MDA)–lysine adducts, and 4-hydroxynonenal (4-HNE) modifications [208]. These neoepitopes are abundantly present on Ox-LDL particles, apoptotic cells, and within atherosclerotic plaques, creating a rich antigenic landscape for both innate-like and conventional B-cell responses.

A particularly well-characterized component of anti-OSE humoral immunity is the E06/T15 natural IgM antibody produced by B-1 cells in a T-cell–independent manner. The E06 antibody (sharing the T15 idiotype originally identified as an anti-*Streptococcus pneumoniae* polysaccharide) specifically recognizes the PC moiety on OxPLs and Ox-LDL [209]. Functionally, E06/T15 IgM neutralizes the pro-inflammatory effects of Ox-LDL, promotes the clearance of apoptotic cells, and inhibits Ox-LDL uptake by macrophages, collectively exerting atheroprotective effects. The causal nature of this protection was established by Que et al., who demonstrated that transgenic overexpression of a single-chain E06 antibody in Ldlr^−^/^−^ mice reduced atherosclerotic lesion formation by up to 28–57%, even in the absence of changes in plasma cholesterol levels [210]. Circulating levels of OxPLs and E06-reactive epitopes on apolipoprotein(a) have been identified as potential biomarkers of cardiovascular risk, with potential utility in risk stratification beyond traditional lipid measurements [211].

The B-cell antigen-specific response to OSE thus operates through two functionally opposing arms: atheroprotective natural IgM antibodies (predominantly B-1 cell-derived) that neutralize and clear OSE, and potentially pro-atherogenic adaptive IgG responses (from follicular B-2 cells) that may form immune complexes and activate complement within plaques (further discussed in Section 3.3.2).

#### 3.1.3. TCR and BCR Repertoire Evidence for Antigen-Driven Clonal Responses

Complementing the antigen-specific studies described above, unbiased high-throughput sequencing of TCR and BCR repertoires has provided additional evidence for antigen-driven adaptive immune responses in atherosclerotic lesions. TCR repertoire analysis of plaque-infiltrating T cells has revealed restricted clonal diversity with oligoclonal expansions, indicating that a limited number of T-cell clones undergo selective proliferation in response to plaque-associated antigens, rather than representing nonspecific bystander infiltration [67]. Similarly, BCR repertoire profiling of B cells in atherosclerotic tissue has identified the enrichment of specific immunoglobulin variable region gene usage and clonotypes, some of which correspond to antibodies reactive against oxidized lipid epitopes [69]. These repertoire-level findings provide unbiased, genome-wide confirmation that adaptive immune responses in atherosclerosis are antigen-driven and clonally focused, supporting the antigen-specific mechanisms detailed in Section 3.1.1 and Section 3.1.2.

Taken together, the evidence summarized in this section establishes that adaptive immunity in atherosclerosis is not merely a process of nonspecific inflammatory amplification but also involves defined autoantigens (ApoB peptides, OSE), specific lymphocyte receptors (tetramer-identified TCR, E06/T15 BCR), and clonally restricted responses (oligoclonal TCR/BCR repertoires). This antigen-specific framework should be kept in mind when interpreting the T- and B-cell subset frequency and functional data presented in the following Section 3.2, Section 3.3 and Section 3.4.

### 3.2. T Cells

#### 3.2.1. Overview: T Cells as Central Mediators of CHD Immunopathology

As key components of the adaptive immune system, T cells contribute to the development, progression, and destabilization of atherosclerotic plaques. Furthermore, they play pivotal roles in endothelial injury, immune senescence, and metabolic–inflammation coupling. Studies over the past five years have demonstrated that T-cell abnormalities in CHD extend far beyond the traditional Th1 pro-inflammatory paradigm, encompassing Treg dysfunction, Th17/Treg imbalance, γδ T cell abnormalities, altered immune checkpoint signaling, CCR5-mediated T-cell recruitment/trafficking, and causal molecular mechanisms of T-cell activation. These advances have significantly deepened our understanding of the immunopathological mechanisms of CHD and provided new targets for precision immunotherapy.

Multi-omics and single-cell studies have revealed substantial compositional and transcriptional alterations in T-cell populations in the immune remodeling of CHD patients. Consistent with the single-cell transcriptomic observations of DAMP-driven immune activation described in Section 2.7.5 [188], T-cell subsets exhibit prominent compositional shifts in CHD, indicating that adaptive immune dysregulation is a central feature of CHD immunopathology. DAMP-driven activation of antigen-presenting cells, together with lipid-derived antigens and chronic inflammation, can promote persistent T-cell activation and effector differentiation., thereby aggravating vascular wall inflammation, smooth muscle cell remodeling, and plaque vulnerability. Consequently, T-cell abnormalities are no longer viewed merely as “concomitant markers of inflammation,” but rather as an important component of the immunopathological framework underlying CHD heterogeneity and progression.

#### 3.2.2. Classical T-Cell Subset Biology in Atherosclerosis

Activated and antigen-experienced T cells are present in atherosclerotic lesions and contribute to adaptive immune responses during plaque development [196,212]. Upon antigen stimulation, CD4^+^ T cells can differentiate into Th1, Th2, Th17, and regulatory T-cell (Treg) subsets, among other specialized and phenotypically plastic populations, each exerting distinct biological effects through characteristic cytokine profiles and participating in immune regulation during plaque development [213]. The pathogenic significance of adaptive T-cell responses has been demonstrated in experimental models in which the transfer of atherosclerosis-associated antigen-specific CD4^+^ T cells into immunodeficient hyperlipidemic mice aggravated lesion development, whereas atherosclerosis was attenuated in models deficient in adaptive lymphocytes [214,215]. However, antigen-specific CD4^+^ T-cell responses are heterogeneous and may exhibit either protective or pathogenic phenotypes depending on disease stage and differentiation state. Atherosclerosis-associated antigens, including ApoB-derived epitopes associated with native or modified LDL, can induce antigen-specific CD4^+^ T-cell responses. Th1 cells produce pro-inflammatory cytokines, particularly IFN-γ and TNF-α, and generally exert pro-atherogenic effects [180]. IFN-γ is a major pro-atherogenic factor that activates macrophages, promotes monocyte infiltration, and drives foam cell formation [216,217]. Th2 cells characteristically produce IL-4, IL-5, and IL-13. IL-5 and IL-13 have generally been associated with atheroprotective effects, whereas the role of IL-4 and the overall contribution of Th2 responses to atherosclerosis remain context dependent [183,184]. Th2 cells are relatively sparse in human atherosclerotic plaques, and their precise contribution to atherogenesis remains incompletely defined. Dendritic cells, including myeloid DCs and plasmacytoid DCs, regulate adaptive immune responses by promoting the differentiation and polarization of naïve CD4^+^ T cells into different effector and regulatory subsets, thereby influencing the balance among Th1, Th2, Th17, and Treg responses within atherosclerotic lesions [186]. The role of Th17 cells and IL-17 signaling in atherosclerosis remains controversial and appears to be context dependent. On the one hand, Th17-derived IL-17 can promote vascular inflammation by stimulating the production of pro-inflammatory cytokines and chemokines [186]. On the other hand, IL-17-associated responses have been reported in some experimental settings to limit IFN-γ production, reduce adhesion molecule expression, and promote type I collagen synthesis by vascular smooth muscle cells, potentially contributing to plaque stabilization [218]. Flow-cytometric analysis of peripheral blood from hyperlipidemic individuals demonstrated a positive correlation between Th17-cell and neutrophil frequencies, while experimental studies in atherosclerotic mouse models further indicated that both cell populations vary across different stages of lesion development [219]. In one clinical cohort, circulating Th1 and Th17 frequencies were higher in patients with stable angina than in those with acute myocardial infarction, highlighting substantial stage- and cohort-dependent heterogeneity in T-cell responses [220].

Clinical evidence of T-cell subset alterations. Patients with CHD have been reported to exhibit lower plasma IL-5 levels than non-CHD controls, with IL-5 concentrations progressively decreasing from stable angina to unstable angina and acute myocardial infarction. In vitro, recombinant IL-5 inhibited ox-LDL-induced Th1 and Th17 differentiation, suggesting that reduced IL-5 signaling may contribute to dysregulated pro-inflammatory T-cell responses in CHD [64]. In patients with CHD combined with major depressive disorder, the CD4^+^/CD8^+^ ratio was significantly higher and CD3^+^CD8^+^ T-cell levels were lower than in CHD patients without major depressive disorder [221]. These findings suggest that neuropsychiatric comorbidity may be associated with additional alterations in peripheral T-cell homeostasis in patients with CHD.

#### 3.2.3. Treg Effects on Post-Infarction Healing

The cardioprotective effects of Tregs described in this subsection are specific to Stage III (post-infarction remodeling) and have been demonstrated primarily in murine myocardial injury models (LAD ligation and ischemia–reperfusion). In this context, Tregs suppress excessive pro-inflammatory responses during the acute post-MI phase and promote the transition to reparative macrophage phenotypes, facilitating adaptive scar formation and preventing adverse ventricular remodeling. While Tregs also exert atheroprotective effects during Stage I (chronic atherogenesis)—primarily through IL-10 and TGF-β-mediated suppression of Th1/Th17 effector responses and macrophage deactivation within the atherosclerotic plaque—the mechanisms, microenvironmental context, and functional outcomes differ substantially between these two stages. Among all T-cell populations, regulatory T cells (Tregs) have emerged as a primary focus in CHD research over the past five years. Tregs maintain immune tolerance and limit vascular inflammation by secreting IL-10 and TGF-β and inhibiting effector T-cell activation [222,223]. The transcription factor Foxp3 serves as the canonical marker for human Tregs. Using genetic Treg ablation (Foxp3-DTR mice) in an MI model, researchers demonstrated that Tregs exert beneficial effects on post-MI wound healing by regulating monocyte/macrophage differentiation [224]. Furthermore, 4-1BB expression on Tregs correlates positively with coronary artery stenosis severity [225].

Th17/Treg imbalance. Numerous studies have demonstrated that CHD patients exhibit decreased Treg numbers or impaired Treg function, often accompanied by a relative increase in Th17 cells, forming a pro-inflammatory dominant state [226]. Emerging clinical evidence indicates that serum NLRP3 levels are inversely correlated with the Treg/Th17 ratio in patients with CHD. Mechanistically, NLRP3 inflammasome activation—originally established as a causal driver of atherogenesis through cholesterol crystal sensing [227]—generates IL-1β, which promotes Th17 polarization [228] while potentially destabilizing Foxp3 expression in Tregs, thereby sustaining and exacerbating coronary inflammation. This finding establishes a mechanistic link between innate immune inflammasomes, adaptive T-cell imbalance, and coronary artery disease progression. The development of CHD is highly influenced by the dynamic interplay between Tregs and Th17 cells, both of which contribute to the establishment of immunological inflammation [219]. Importantly, the Th17/Treg imbalance observed in CHD patients is a nonspecific perturbation of immune homeostasis that also has a defined antigen-specific basis. As described in Section 3.1.1, MHC II-tetramer studies have demonstrated that ApoB-reactive CD4^+^ T cells, which predominantly display a Foxp3^+^ Treg phenotype in healthy individuals, undergo phenotypic drift toward Th1 and Th17 effector states in patients with cardiovascular disease [205,206]. This antigen-specific Treg-to-effector conversion provides a direct molecular mechanism for the clinical observation that Treg frequency decreases and Th17 frequency increases as the disease progresses. The CCR5-mediated Treg dysfunction described above may further facilitate this phenotypic drift, as chemokine receptor signaling can modulate the stability of the Foxp3 locus in antigen-experienced Treg cells [206]. These findings elevate the Th17/Treg imbalance narrative from a descriptive clinical observation to a mechanistically grounded account of antigen-specific autoreactive T-cell plasticity in atherosclerosis. Metabolic receptor signaling on Tregs. A recent study by Lv et al. (2025) further linked aberrant Treg function to metabolic receptor signaling, reporting that GLP-1 receptor expression on Tregs correlated with the severity of coronary stenosis and inflammation dysregulation [229]. This finding is particularly novel because the GLP-1 receptor is conventionally associated with glucose metabolism and cardiovascular protection. Its expression on Tregs may reflect or regulate the immune inflammatory state in CHD patients, providing a potential immunological explanation for the observation that GLP-1 receptor agonists improve the inflammatory microenvironment of CHD through Treg modulation.

Early Treg reprogramming in at-risk individuals. Functional reprogramming of Tregs has been demonstrated in individuals susceptible to early atherosclerosis. In young individuals with dyslipidemia, Tregs exhibit a mixed Th1/Th17/Treg phenotype with decreased suppressive function. Treatment with the CCR5 inhibitor DAPTA restored the immunosuppressive phenotypes and functions of both Tregs and myeloid-derived suppressor cells (M-MDSCs), while decreasing IL-12 and increasing IL-10 [230]. This suggests that Treg dysfunction may precede clinically overt CHD and represents a key target for early intervention.

#### 3.2.4. CCR5–CCL5 Axis: Chemotactic Migration and Vascular Inflammation

Chemotactic receptor signaling plays a critical role in T-cell recruitment and vascular inflammation. The CCR5–CCL5 axis has emerged as a focal point in CHD immune regulation. A 2025 study demonstrated that CCR5-related immune abnormalities contribute to the progression of atherosclerosis by promoting immune cell adhesion and migration across dysfunctional endothelium in asymptomatic individuals exposed to CHD risk factors [230]. Importantly, CCR5 inhibition not only affects cell migration but also corrects Treg dysfunction (see Section 3.2.3), establishing a dual therapeutic rationale for targeting this axis.

#### 3.2.5. γδ T Cells: Bridging Innate and Adaptive Immunity

Beyond classical CD4+ helper T cells and Tregs, non-classical T-cell subsets have attracted increasing attention. Li et al. (2022) demonstrated significant quantitative and phenotypic alterations in peripheral γδ T cells and their subsets in patients with CAD [65]. Beyond classical CD4+ helper T cells and Tregs, non-classical T-cell subsets have attracted increasing attention. Li et al. (2022) demonstrated significant quantitative and phenotypic alterations in peripheral γδ T cells and their subsets in patients with CAD [65]. Compared with healthy controls, patients with CAD showed reduced frequencies and absolute numbers of total γδ T cells and Vδ2^+^ T cells, together with an altered Vδ1/Vδ2 ratio and reduced expression of several activation- and immune-function-related surface markers. These findings indicate substantial dysregulation rather than simple activation of the γδ T-cell compartment in CAD.

As detailed in Section 4.3.2, γδ T cells possess both innate-like and adaptive immune characteristics. Experimental studies suggest that γδ T cells, particularly IL-17-producing subsets, may contribute to early atherosclerotic inflammation; however, their effects appear to be subset-, tissue-, and disease-stage-dependent, and findings from different animal models have not been entirely consistent. In human CAD, current evidence remains largely based on peripheral immunophenotyping and association studies. Therefore, γδ T cells represent a potentially important but still insufficiently characterized component of CHD immunopathology and may constitute a future therapeutic or biomarker target after further mechanistic and clinical validation.

#### 3.2.6. Immune Checkpoint Pathways in CHD: From Endogenous Immunoregulation to Therapeutic Cardiotoxicity

**Figure 4:**
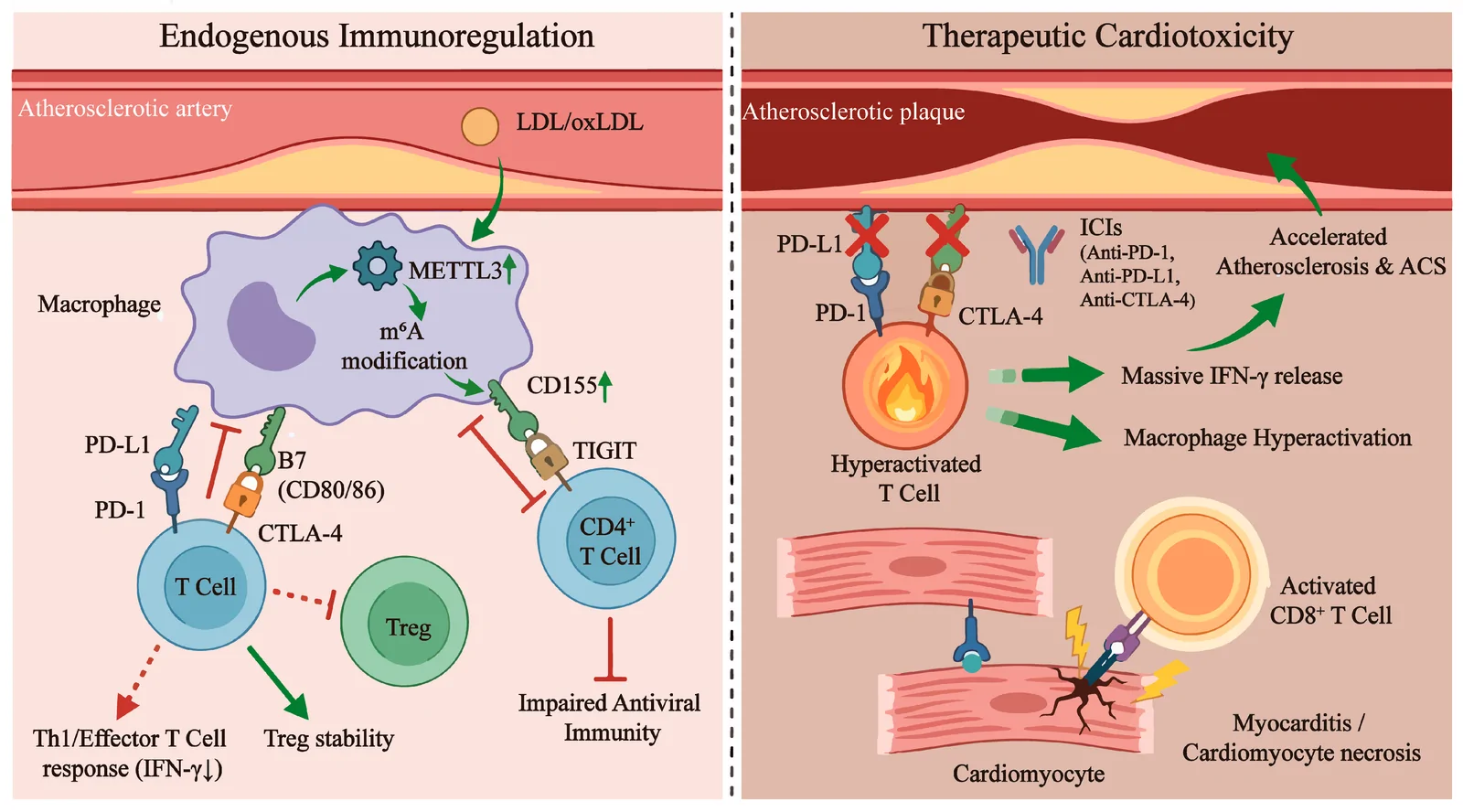
Immune checkpoint-mediated endogenous immunoregulation in coronary heart disease and cardiovascular toxicity associated with immune checkpoint inhibitors. The left panel illustrates endogenous immune checkpoint regulation within the atherosclerotic milieu. PD-L1 and B7 (CD80/CD86) expressed on macrophages/antigen-presenting cells engage PD-1 and CTLA-4, respectively, on T cells, thereby restraining T-cell activation, Th1/effector T-cell responses, and IFN-γ production while supporting Treg stability. These inhibitory pathways limit vascular inflammation and atherosclerotic progression. In parallel, LDL/oxidized LDL (oxLDL) promotes increased METTL3 expression in macrophages from patients with coronary artery disease (CAD), enhancing m^6^A modification and stability of PVR/CD155 mRNA and consequently increasing surface CD155 expression. CD155 engagement of TIGIT on CD4^+^ T cells suppresses clonal expansion and antiviral effector responses, resulting in impaired antiviral immunity. The right panel depicts cardiovascular toxicity induced by immune checkpoint inhibitors (ICIs). Blockade of PD-1, PD-L1, or CTLA-4 removes inhibitory immune restraint, leading to enhanced effector T-cell activation, increased IFN-γ production, and macrophage activation, thereby accelerating atherosclerotic progression and increasing the risk of acute coronary syndrome (ACS). Disruption of protective PD-1/PD-L1 signaling also permits activated CD8^+^ T-cell-mediated cardiomyocyte injury and necrosis, contributing to ICI-associated myocarditis. Green solid arrows indicate stimulatory or enhancing effects, red inhibitory lines/dashed lines indicate suppressive regulation, and red crosses denote ICI-mediated blockade of immune checkpoint interactions.

Immune checkpoint receptors are co-inhibitory molecules that maintain peripheral tolerance and prevent excessive immune activation. In atherosclerosis, these pathways serve dual and context-dependent roles: they restrain anti-plaque immune responses but may also contribute to dysfunctional T-cell states. Moreover, the clinical use of immune checkpoint inhibitors (ICIs) in oncology has revealed that disruption of these pathways can precipitate severe cardiovascular complications, underscoring their functional importance in cardiovascular immune homeostasis (Figure 4).

##### PD-1/PD-L1 Axis in Atherosclerosis and CHD

The programmed cell death protein-1 (PD-1) receptor and its ligands PD-L1 (B7-H1/CD274) and PD-L2 (B7-DC/CD273) constitute a major co-inhibitory checkpoint that attenuates T-cell receptor signaling, limits effector T-cell proliferation and cytokine production, and promotes peripheral immune tolerance [212].

Endogenous role in atherosclerosis. PD-1 is expressed on T cells infiltrating human atherosclerotic plaques, and its expression increases with lesion progression [212]. Experimental studies have demonstrated that genetic deficiency of PD-1 or PD-L1 in hyperlipidemic mouse models significantly exacerbates atherosclerotic lesion development, with increased plaque T-cell infiltration, enhanced IFN-γ production, and elevated macrophage activation [231,232]. These findings establish PD-1/PD-L1 signaling as an endogenous atheroprotective brake that constrains pro-inflammatory T-cell responses within the vascular wall.

Clinical associations in CHD. In patients with coronary artery disease, altered PD-1 expression on circulating CD4^+^ and CD8^+^ T cells has been reported. Several studies have identified increased frequencies of PD-1^+^ T cells in peripheral blood of patients with acute coronary syndromes (ACS), potentially reflecting chronic antigen stimulation and T-cell exhaustion [212]. Conversely, soluble PD-L1 (sPD-L1) levels have been associated with inflammatory burden and cardiovascular outcomes in CHD cohorts [233]. The functional significance of PD-1^+^ T cells in CHD remains under active investigation: while PD-1 expression may indicate an exhausted, hypofunctional state that limits further tissue damage, the loss of PD-1-mediated suppression—whether through receptor downregulation, ligand insufficiency, or therapeutic blockade—can unleash pro-atherogenic T-cell effector responses.

Interaction with Treg biology. PD-1 signaling promotes the differentiation and maintenance of induced Tregs (iTregs) and enhances Foxp3 stability [234]. Disruption of PD-1/PD-L1 interactions may therefore contribute to the Treg-to-effector phenotypic drift described in Section 3.1.1, providing an additional mechanistic link between checkpoint dysfunction and the Th17/Treg imbalance observed in CHD patients (Section 3.2.3).

##### CTLA-4 in Atherosclerosis and CHD

Cytotoxic T-lymphocyte-associated protein 4 (CTLA-4/CD152) is a co-inhibitory receptor that competes with the co-stimulatory receptor CD28 for binding to B7-1 (CD80) and B7-2 (CD86) on antigen-presenting cells, thereby attenuating T-cell activation at the priming stage [235].

Experimental evidence. CTLA-4 blockade or deficiency in atherosclerosis-prone mice accelerates lesion development and increases plaque inflammation, with augmented Th1 responses and reduced Treg suppressive capacity [236]. Conversely, CTLA-4-Ig fusion proteins, which block CD28-mediated co-stimulation, have shown atheroprotective effects in experimental models, reducing lesion size and T-cell activation within plaques. Clinical relevance. Polymorphisms in the CTLA-4 gene have been associated with susceptibility to coronary artery disease in several population-based studies, although findings are not entirely consistent across ethnic groups [236].

##### Additional Inhibitory Receptors on Adaptive and Innate Immune Cells

Beyond PD-1 and CTLA-4, several additional co-inhibitory receptors have emerged as modulators of vascular inflammation. The counterpart receptors for CD155/PVR are TIGIT (an inhibitory molecule, in addition to CD96, which is another inhibitory molecule) and DNAM1 (an activating molecule), which are expressed on innate and adaptive effector cells.

TIGIT (T-cell immunoreceptor with Ig and ITIM domains): expressed on T cells and NK cells within atherosclerotic lesions; TIGIT^+^ Tregs exhibit enhanced suppressive function, and TIGIT deficiency exacerbates experimental atherosclerosis [237,238].

TIM-3 (T-cell immunoglobulin and mucin domain-3): expressed on exhausted T cells and macrophages in plaques; TIM-3 signaling regulates macrophage efferocytosis and inflammasome activation [239]. Galectin-3 is one of the putative ligands for TIM-3. The TIM-3–Gal-9 interaction plays an important immunoregulatory role by promoting T-cell exhaustion and modulating innate immune responses, which may be relevant to vascular inflammation.

LAG-3 is a negative regulator of T-cell expansion that inhibits early events in primary activation of human CD4 and CD8 T cells. Blocking LAG-3/MHC class II interactions augments CD69 expression, T-cell expansion, and Th1 cytokine production. Soluble LAG-3 levels are significantly lower in patients with CAD compared with controls and are negatively associated with BMI and diabetes mellitus, suggesting that reduced sLAG-3 may represent a novel risk factor for CAD development. Plasma LAG-3 has also been identified as a potential independent predictor of HDL-C levels and CHD risk [240].

Inhibitory receptors on innate cells: macrophages, dendritic cells, and NK cells within atherosclerotic lesions express a repertoire of inhibitory receptors including SIRPα (interacting with CD47), LILRB2/ILT4, and Siglec family members, which regulate phagocytosis, antigen presentation, and cytokine production [241,242]. These innate immune checkpoints represent an expanding area of investigation in cardiovascular immunology.

##### CD155/METTL3 Axis: Macrophage-Mediated Checkpoint Dysregulation

A mechanistic study demonstrated that patients with CAD exhibit excessive CD155-mediated inhibitory signaling that suppresses antiviral CD4^+^ T-cell responses. Importantly, the primary abnormality identified in this study was not intrinsic overactivation of CD155 in T cells. Instead, macrophages from patients with CAD overexpressed the methyltransferase METTL3, which increased N^6^-methyladenosine (m^6^A) modification and stability of PVR/CD155 mRNA, resulting in excessive surface expression of the immunoinhibitory ligand CD155. CD155^high^ macrophages subsequently delivered inhibitory signals to antigen-reactive CD4^+^ T cells expressing TIGIT and/or CD96, thereby suppressing their clonal expansion and antiviral effector responses [243]. This mechanism extends the concept of immune dysfunction in CHD beyond chronic vascular inflammation by demonstrating that a pro-inflammatory cardiovascular environment can coexist with impaired pathogen-specific adaptive immunity. The study specifically demonstrated reduced CD4^+^ T-cell responses to SARS-CoV-2 and Epstein–Barr virus antigens and showed that LDL and oxidized LDL could promote the METTL3^high^ CD155^high^ macrophage phenotype. These observations reveal an important interaction between dyslipidemia, macrophage reprogramming, immune checkpoint signaling, and defective antiviral T-cell immunity in CAD. However, the available evidence does not establish generalized systemic immunosuppression in all patients with CHD, nor does it directly demonstrate that CD155-mediated T-cell inhibition causes post-infectious cardiovascular events. Rather, it identifies a specific mechanism by which antiviral CD4^+^ T-cell responses can be impaired in the setting of CAD.

This finding positions CD155 alongside PD-L1 and B7 ligands as a macrophage-expressed immunomodulatory molecule that shapes T-cell function in the atherosclerotic milieu, and highlights the role of epitranscriptomic regulation (m^6^A modification) in checkpoint ligand expression—a mechanism not yet described for PD-L1 or CTLA-4 ligands in this context.

##### Immune Checkpoint Inhibitor-Associated Cardiovascular Toxicity

The critical importance of immune checkpoints in cardiovascular homeostasis is dramatically illustrated by the cardiovascular toxicities observed during therapeutic ICI use in oncology. ICIs—including anti-PD-1 (nivolumab, pembrolizumab), anti-PD-L1 (atezolizumab, durvalumab), and anti-CTLA-4 (ipilimumab) antibodies—have revolutionized cancer treatment but are associated with a spectrum of cardiovascular adverse events [244].

ICI-associated myocarditis. ICI-related myocarditis is the most feared cardiovascular complication, occurring in approximately 0.5–1.7% of ICI-treated patients but carrying a case fatality rate of 25–50% [245,246]. Histopathologically, ICI myocarditis is characterized by dense T-cell (predominantly CD8^+^) and macrophage infiltration of the myocardium, with evidence of cardiomyocyte necrosis [247]. Combination therapy with anti-PD-1 and anti-CTLA-4 confers the highest risk, consistent with synergistic removal of immune restraint [248]. Mechanistically, PD-L1 is constitutively expressed on cardiomyocytes and serves as a protective mechanism against autoreactive T-cell-mediated damage; its blockade permits T-cell-mediated cardiac injury [249].

ICI-associated accelerated atherosclerosis and ACS. Emerging evidence indicates that ICI therapy is associated with accelerated atherosclerotic progression and increased risk of acute cardiovascular events. A landmark study by Drobni et al. (2020) demonstrated that ICI-treated cancer patients had a three-fold increased rate of atherosclerotic cardiovascular events (myocardial infarction, coronary revascularization, ischemic stroke) compared with matched controls, with aortic plaque volume progression rates increasing by more than 300% after ICI initiation [250]. Subsequent studies have confirmed increased rates of acute coronary syndromes, aortic plaque inflammation, and coronary artery calcification in ICI-treated populations [251].

Clinical implications. These observations provide compelling “loss-of-function” evidence in humans that PD-1/PD-L1 and CTLA-4 checkpoints are functionally essential for restraining atherosclerotic inflammation and maintaining cardiovascular immune homeostasis. They also highlight the need for cardiovascular risk assessment and monitoring in ICI-treated patients, and raise the possibility that targeted restoration of checkpoint signaling could represent a novel atheroprotective therapeutic strategy.

#### 3.2.7. Genetic Causal Inference and Drug Target Discovery

With the advancement of genetic epidemiology and causal-inference approaches, the identification of T-cell-related molecular targets in CAD has entered a new stage. A transcriptome-wide Mendelian randomization study integrating dynamic single-cell eQTL data across different stages of CD4^+^ T-cell activation evaluated the putative causal effects of genetically predicted gene expression on coronary artery disease and type 2 diabetes [174]. The analysis identified multiple gene–CAD associations supported by Mendelian randomization and colocalization evidence, including activation-state- and T-cell-subset-specific signals. It also prioritized a number of immune-related genes with potential relevance to drug development or drug repurposing [174]. These findings suggest that gene-expression programs operating during CD4^+^ T-cell activation may contain molecular determinants that contribute to CAD risk rather than merely reflecting secondary inflammatory responses. Nevertheless, Mendelian randomization supports putative causal effects of genetically predicted gene expression and does not by itself demonstrate that the corresponding proteins or pharmacological interventions will produce equivalent clinical effects. Thus, these results are best viewed as a strategy for prioritizing candidate causal genes and therapeutic targets for subsequent functional and clinical validation. This research paradigm advances T-cell research in CAD from conventional association studies toward genetically informed causal inference and target prioritization.

A review published online in 2025 further highlighted Tregs as a potentially shared immunoregulatory axis linking psoriasis and atherosclerosis [252]. Tan et al. summarized evidence that Treg dysfunction may contribute to both psoriasis-associated IL-17/IL-23-driven inflammation and atherosclerotic inflammatory responses, and proposed Treg-centered mechanisms as a framework for understanding psoriasis–atherosclerosis comorbidity. Because this work is a review rather than a CHD-specific causal or interventional study, it should not be interpreted as direct evidence that Treg dysfunction causes coronary disease in patients with psoriasis. Nevertheless, it illustrates how disturbances in immune tolerance and T-cell regulation may be shared across cardiovascular and systemic chronic inflammatory diseases. This comorbidity-immunology perspective supports investigation of common immune pathways that may contribute to the excess cardiovascular burden associated with chronic inflammatory disorders.

#### 3.2.8. Summary, Limitations, and Future Directions

Recent research on T cells in CHD reveals several major trends. First, the field has expanded beyond the traditional Th1-dominated model toward parallel characterization of multiple T-cell populations, including Tregs, Th17 cells, γδ T cells, and senescent or highly differentiated T-cell phenotypes [65,253]. Second, research has shifted from measuring changes in circulating T-cell numbers alone toward characterizing functional state, differentiation, chemokine-guided trafficking, immune checkpoint signaling, immunosenescence, and metabolic–immune interactions. Third, emerging approaches, including single-cell sequencing, dynamic eQTL analysis, and Mendelian randomization, have improved the ability to distinguish cell-state-specific associations from genetically supported candidate causal mechanisms [188].

However, several limitations persist. First, many clinical studies rely primarily on peripheral blood samples, which may not accurately reflect the abundance, phenotype, clonality, or functional state of T cells within coronary atherosclerotic plaques. Second, T-cell subset definitions and phenotypic markers are not fully standardized across studies. Differences in the identification of Tregs, highly differentiated or senescent T cells, and effector T-cell subsets can complicate cross-study comparisons. Third, many findings concerning newly identified T-cell populations, immune checkpoints, and molecular targets remain observational, computational, or preclinical and lack validation in large prospective cohorts or randomized intervention trials. Fourth, substantial heterogeneity related to disease stage, age, metabolic status, medications, and comorbid inflammatory conditions can influence circulating and tissue T-cell phenotypes and should be more carefully addressed in future studies. Future research should integrate coronary plaque tissue analysis with spatial transcriptomics, single-cell multi-omics, T-cell receptor repertoire analysis, and longitudinal clinical cohorts to clarify the spatial and temporal dynamics of T-cell subsets during atherosclerosis progression and acute coronary events. Genetically prioritized targets identified by Mendelian randomization should also undergo functional validation before being considered established therapeutic targets. Overall, T cells represent an important mechanistic component of CHD immunopathology and a potential source of biomarkers and therapeutic targets. The substantial T-cell immune remodeling observed in CHD—including Treg dysfunction, altered Th17/Treg balance, changes in chemokine-guided trafficking, γδ T-cell dysregulation, immunosenescent T-cell phenotypes, and excessive CD155-mediated inhibitory signaling—indicates that T-cell abnormalities can contribute to both chronic vascular inflammation and alterations in systemic immune responses. However, the roles of individual T-cell subsets are highly context dependent and vary according to disease stage, tissue compartment, and inflammatory environment. Precision immunomodulatory strategies targeting specific T-cell pathways may therefore have future value in CHD, but their utility for risk stratification, prevention, or individualized treatment requires prospective clinical validation.

### 3.3. B Cells

#### 3.3.1. Overview: Heterogeneous Roles of B-Cell Subsets in Atherosclerosis

The role of B cells in atherosclerosis is markedly heterogeneous, with different subsets exerting distinct and sometimes opposing effects on disease progression. Current evidence has established a subset-specific framework for understanding B-cell functions in atherosclerosis. B1 cells are generally considered atheroprotective, largely through the production of natural IgM antibodies that recognize oxidation-specific epitopes on modified lipids and apoptotic cells, thereby facilitating their neutralization and clearance. In contrast, the effects of B2 cells are more heterogeneous. Follicular B2 cells are generally considered pro-atherogenic in experimental models because they can promote antigen-specific antibody responses and T-cell activation, whereas marginal-zone B cells can exert atheroprotective effects through mechanisms including IgM production and regulation of T-cell responses. Beyond antibody production, B cells can modulate local and systemic immune responses through cytokine secretion, including IL-10, TNF-α, and IL-6, depending on B-cell subset and activation state. B cells also function as antigen-presenting cells and regulate T-cell responses through MHC-II-dependent antigen presentation and co-stimulatory pathways, including CD40–CD40L interactions [254]. Consequently, alterations in B-cell populations in CHD may reflect not only changes in humoral immunity but also broader remodeling of adaptive immune regulation. However, the effects of B cells should be interpreted at the level of specific subsets rather than as a uniform pro- or anti-atherogenic function.

#### 3.3.2. B-Cell Subset Biology in Atherogenesis

B cells contribute to both innate-like and adaptive immune responses through antibody production, cytokine secretion, antigen presentation, and interactions with other immune cells. Their effects in atherosclerosis are strongly subset dependent. B1 cells, particularly B1a and B1b populations in experimental models, generally exert atheroprotective effects. Natural IgM antibodies produced by B1 cells recognize apoptotic-cell determinants and oxidation-specific epitopes on oxidized LDL (ox-LDL), facilitating the clearance or neutralization of these antigens and limiting their uptake by macrophages [209]. Thus, the effects of B2 cells on atherogenesis depend substantially on the specific B-cell subset, activation state, and experimental context. The extent to which these murine B-cell classifications and functions can be directly extrapolated to human CHD remains incompletely defined. [255]. OSEs are the most well-characterized antigenic targets of the humoral immune response in atherosclerosis. Generated during oxidative modification of LDL and cellular membranes, OSEs include PC on OxPLs, MDA–protein adducts, and 4-HNE modifications (reviewed comprehensively in Section 3.1.2) [208]. Humoral responses to OSEs occur via functionally distinct antibody classes. B-1 cell-derived natural IgM antibodies, particularly those of the E06/T15 clonotype, recognize PC on OxLDL and exert atheroprotective effects by neutralizing OxPL-mediated inflammation, promoting apoptotic cell clearance, and inhibiting macrophage foam cell formation [209]. The causal atheroprotective role of E06 was established by transgenic overexpression studies demonstrating a 28–57% reduction in atherosclerotic lesion area in Ldlr^−^/^−^ mice [210]. Clinically, OxPL/ApoB- and E06-reactive epitope levels are independently associated with cardiovascular events and aortic valve calcification, suggesting their translational biomarker potential [211]. In contrast, class-switched IgG antibodies against OSE produced by conventional B-2 cells following T-cell-dependent germinal center reactions may exert pro-atherogenic effects through immune complex formation and complement activation within plaques [208].This dual humoral response to OSE, comprising atheroprotective IgM versus potentially proatherogenic IgG, provides an antigen-specific mechanistic basis for the functionally opposing roles of B-1 and B-2 cells discussed below.

#### 3.3.3. Regulatory B Cells: Controversial but Clinically Relevant

The role of regulatory B cells (Bregs) in atherosclerosis remains incompletely defined. Experimental studies using different Breg populations, including IL-10-producing B cells and B10-like subsets, have produced inconsistent results, partly because Bregs lack a universally accepted lineage-defining phenotype and are frequently defined according to functional properties rather than a single set of surface markers [256,257]. A clinical pilot study comparing patients with acute myocardial infarction (MI), stable angina pectoris, and controls found no significant difference in the frequency of CD24^+^CD27^+^ B10-like cells among the groups. In contrast, circulating CD20^+^CD24^hi^CD38^hi^ transitional Breg-like cells were significantly reduced in patients with acute MI compared with patients with stable angina [116]. Therefore, the available evidence does not support a generalized reduction in all circulating Breg populations during MI. Rather, it suggests that specific regulatory B-cell phenotypes may be differentially altered during acute coronary events. Whether these changes contribute causally to plaque destabilization or instead arise as a consequence of acute inflammation remains unknown.

A separate transcriptomic study involving patients with acute MI, stable angina, and healthy volunteers identified extensive dysregulation of B-cell-associated genes during acute MI [205]. Expression changes among B-cell receptor (BCR)-related genes were heterogeneous: some genes, including CD45, NFAM, SYK, and LYN, were upregulated, whereas others, including CD79B, CD19, CD81, FYN, BLK, CD22, and CD5, were downregulated. Alterations were also observed in genes associated with T-dependent B-cell responses and B-cell regulatory pathways. The authors interpreted these findings as evidence of impaired B2-cell activation and weakened T-cell–B-cell interactions and proposed enhancement of B2-cell-mediated humoral immunity as a potential therapeutic concept [205]. However, this hypothesis was derived from a small observational transcriptomic study and should be interpreted cautiously, particularly because experimental studies have demonstrated pro-atherogenic effects of follicular B2 cells and beneficial effects of B2-cell depletion in several preclinical settings. Therefore, enhancement of B2-cell immunity cannot currently be considered an established therapeutic strategy for MI or CHD.

#### 3.3.4. Multi-Omics Evidence for B-Cell Immune Remodeling in CHD

Multi-omics studies consistently indicate that B cells are an important component of peripheral immune remodeling in CHD patients. Several key findings have emerged: Chen et al. (2024) found increased proportions of B cells and activated dendritic cells in the peripheral blood of CHD patients. The high CHD score group was characterized by greater infiltration of neutrophils and monocytes in the context of necroptosis and immune infiltration [35]. This study suggests that B cells may participate in the amplification of necroptosis-related inflammatory responses, particularly in the context of DAMP release and innate immune activation, jointly shaping adaptive immune responses with dendritic cells and T cells.

Furthermore, a recent study employing multiple machine learning algorithms to establish genetic diagnostic models for CHD with immune-related features identified CD8^+^ T cells and naïve B cells as the most important abnormal immune cell populations in peripheral blood [258]. These results support the concept that B-cell subset changes, rather than total B-cell number, may better reflect the immune status of CHD. Naïve B-cell alterations, in particular, suggest possible remodeling of the processes involved in B-cell development, activation, or differentiation into effector/memory phenotypes.

#### 3.3.5. B Cells and Subclinical Coronary Artery Calcification

A longitudinal exploratory analysis from the Multi-Ethnic Study of Atherosclerosis examined associations between circulating immune-cell subsets and the incidence and progression of coronary artery calcification (CAC) [259]. Among participants with detectable CAC at baseline, each 1-standard-deviation increase in the proportion of circulating B cells was associated with approximately 53.1 Agatston units greater CAC progression during follow-up. In contrast, the study did not establish that higher B-cell proportions increased the incidence of CAC among participants without baseline calcification. These findings suggest that circulating B-cell abundance may be associated with the progression of established subclinical coronary calcification. However, the analysis was exploratory and observational and therefore does not demonstrate that B cells directly promote vascular calcification. Moreover, total circulating B-cell frequency does not distinguish potentially protective from potentially pathogenic B-cell subsets. The association should therefore be interpreted as a signal requiring replication and mechanistic investigation rather than evidence for a direct calcification-promoting effect of B cells. Together with the reported association between NK-cell abundance and incident CAC described in Section 2.6.2, these observations suggest that distinct immune-cell populations may be associated with different aspects of subclinical coronary atherosclerosis. Whether these immune signatures participate directly in calcification or instead reflect the underlying inflammatory and metabolic environment remains to be determined.

#### 3.3.6. B-Cell Surface Molecules: CD27 as an Emerging Target

The role of B-cell-associated membrane molecules in CHD represents a new direction of investigation. Recent investigations by Song et al. elucidated the mechanistic role of CD27 in coronary atherosclerosis. CD27, a member of the TNF receptor superfamily, is mainly expressed on memory B cells, some T cells, and plasma cell precursors and participates in B-cell activation, survival, and antibody responses [260]. This finding demonstrates that B-cell abnormalities in CHD are reflected not only in cell number and subset proportions but also in key surface molecules and co-stimulatory pathway reprogramming, providing a basis for the future development of B-cell-related biomarkers and immunotherapy targets.

#### 3.3.7. Immunosenescence and Lipid–Immune Interactions

Current perspectives on immunosenescence highlight that CHD is not exclusively a lipid-driven disorder but is intrinsically linked to immune aging. B cells, like T cells and macrophages, are important effector cells in the immunosenescence process. A recent Mendelian randomization analysis systematically evaluated the causal link between immune cells, lipids, and cardiovascular disease, demonstrating that immune cell subsets and lipid characteristics jointly affect CVD risk and emphasizing the importance of immune–lipid interactions for targeted therapy [261]. These perspectives provide a theoretical basis for re-understanding the role of B cells in CHD and metabolic inflammation, particularly in elderly populations.

#### 3.3.8. Summary, Limitations, and Future Directions

Research over the past five years has elevated the role of B cells in CHD from that of traditional “antibody generators” to that of multifunctional immune regulatory cells operating at multiple levels: antigen presentation, cytokine secretion, T-cell regulation, and lipid–immune crosstalk [188]. Transcriptome, single-cell, and immune infiltration analyses consistently indicate B-cell abnormalities in CHD patients, especially naïve B-cell alterations and altered global B-cell infiltration patterns. The finding that B cells are associated with CAC progression suggests a unique role in subclinical coronary structural damage. CD27 and other B-cell-related molecules may become new diagnostic or mechanistic research targets [262]. he perspectives of immunosenescence and lipid–immune interaction provide a theoretical basis for understanding B-cell contributions to CHD and metabolic inflammation in the elderly [261]. However, several limitations remain: (1) Subset resolution: Most studies use total B-cell populations or limited markers, lacking detailed stratification of B1 cells, B2 cells, Bregs, plasmablasts, and memory B cells. (2) Sample source: The majority of evidence derives from peripheral blood rather than local coronary plaque tissue, which cannot fully reflect B-cell behavior at the lesion site. (3) Functional validation: Although bioinformatics results are abundant, functional validation through mechanistic experiments remains insufficient. Future research should integrate single-cell sequencing, spatial transcriptomics, plaque tissue immunophenotyping, and animal functional experiments to clarify the disease-promoting or protective effects of different B-cell subsets at different stages of CHD.

### 3.4. Summary of Adaptive Immunity in CHD

Studies in both CHD patients and animal models strongly support the role of immune dysregulation in cardiac damage. Autoreactivity and sterile inflammation are inextricably intertwined with CHD pathogenesis. The physiological purpose of these immune responses is likely the clearance of modified endogenous lipids and cellular debris from the arterial wall; however, chronic immune dysregulation ultimately drives plaque progression. Within the immune system, there exists a self-regulatory balance between pro-inflammatory and anti-inflammatory responses, and modulating this self-regulatory balance may offer novel strategies for the treatment of CHD.

## 4. Discussion

### 4.1. Immune Network of CHD: From Individual Cell Types to Systems-Level Integration

**Figure 5:**
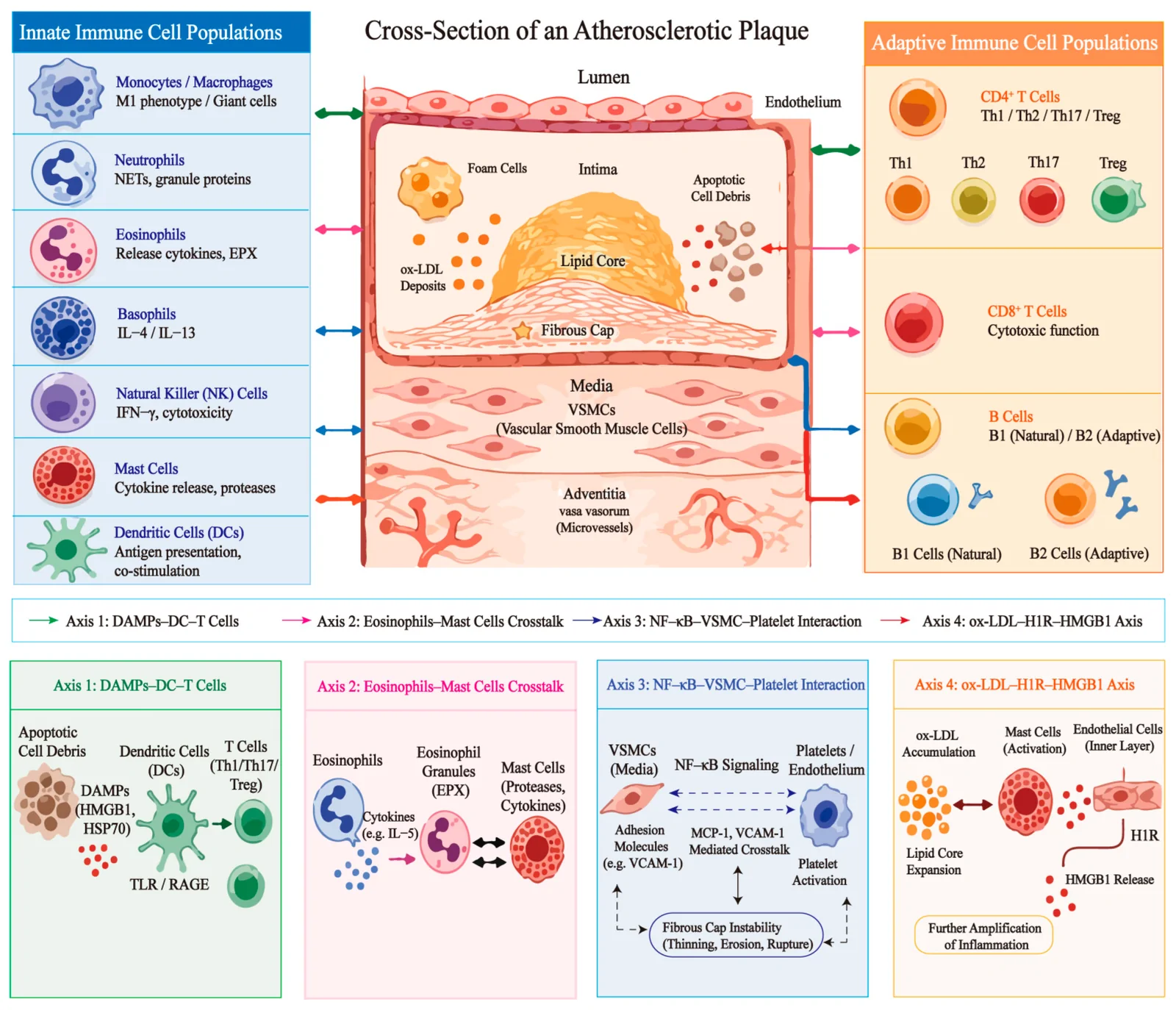
Immune Network Architecture in Coronary Artery Disease: Transcellular Signaling Axes in Atherosclerotic Plaque Progression. Schematic representation of the interconnection network of innate and adaptive immunity within the atherosclerotic artery wall. The central panel shows a cross-section of the diseased artery, including the endothelium, intima (containing foam cells, lipid core, and necrotic debris), media (containing vascular smooth muscle cells, VSMCs), and adventitia. Left panel: innate immune cell populations. Right panel: adaptive immune cell populations. Colored arrows and bottom panels highlight the four key transcellular signaling axes discussed in Section 4.1: (1) Green: DAMPs–DC–T cell axis; (2) Pink: eosinophil–mast cell co-infiltration network; (3) Blue: NF-κB-dependent VSMC-monocyte crosstalk axis; (4) Red: ox-LDL–histamine–H1R–HMGB1 axis. This figure illustrates that the pathogenesis of CHD involves a systems-level immune network rather than isolated cellular responses. Different arrow types represent distinct biological interactions: solid arrows indicate direct regulation, while dashed arrows denote indirect or regulatory crosstalk. Abbreviations: DAMPs, damage-associated molecular patterns; DCs, dendritic cells; EPX, eosinophil peroxidase; H1R, histamine receptor 1; HMGB1, high-mobility group box 1; IFN-γ, interferon-γ; MCP-1, monocyte chemoattractant protein-1; NETs, neutrophil extracellular traps; NF-κB, nuclear factor kappa B; ox-LDL, oxidized low-density lipoprotein; Th, T helper cells; TLR, toll-like receptor;

The preceding sections have systematically reviewed the roles of individual immune cell types in CHD, spanning monocytes and macrophages (Section 2.2 and Section 2.3), neutrophils (Section 2.4), eosinophils and basophils (Section 2.5), NK cells (Section 3.2.6), dendritic cells (Section 2.7), mast cells (Section 2.8), T cells (Section 3.2), and B cells (Section 3.3). A central theme emerging from this body of evidence is that no single immune cell type operates in isolation. Rather, CHD is driven by a dynamic, interconnected immune network in which innate and adaptive immune cells engage in continuous bidirectional crosstalk. These examples emphasize that future research and therapeutic strategies must adopt a systems-level perspective rather than targeting individual cell populations in isolation (Figure 5).

Several intercellular signaling axes illustrate this network architecture. The DAMPs–DC–T cell axis (Section 2.7.5) describes how danger signals released from necrotic plaque tissue activate dendritic cells, which in turn polarize T-cell responses toward pro-inflammatory Th1/Th17 phenotypes [176,188]. The eosinophil–mast cell co-infiltration network (Section Eosinophils in Immune Infiltration Networks and Microvascular Dysfunction and Section 2.7.2) reveals coordinated innate immune cell recruitment driven by neutrophil degranulation products [42]. The NF-κB-dependent crosstalk between vascular smooth muscle cells and monocytes (Section 2.2.5) establishes a checkpoint for plaque stabilization that operates independently of adaptive immunity [118]. The ox-LDL–histamine–H1R–HMGB1 axis linking mast cells to endothelial injury (Section 2.7.4) demonstrates how lipid toxicity and immune activation converge at the molecular level [193]. These examples underscore that future research and therapeutic strategies must adopt a systems-level perspective rather than targeting individual cell populations in isolation.

### 4.2. Converging Pathological Endpoints: Subclinical Calcification as a Shared Immune Target

A striking finding across multiple immune cell types is their shared association with coronary artery calcification (CAC) at the subclinical stage. NK cells (Section 2.6.2, HR = 1.26 per SD in the MESA study) [161], B cells (Section 3.3.5, 53.1-unit increase in Agatston score per SD) [260], and monocyte-derived inflammatory indices [31,111] have all been independently linked to CAC progression. This convergence suggests that immune-mediated vascular calcification represents a common downstream pathway through which diverse immune cell types contribute to early coronary structural damage. The identification of CAC as a shared immune target has important clinical implications: immune biomarker panels incorporating multiple cell types may improve the prediction of subclinical disease beyond traditional risk factors, potentially enabling earlier intervention in at-risk populations.

### 4.3. EAT as an Immunological Microenvironment: Convergence of Innate and Adaptive Immunity

This review discusses the independent roles of T cells, γδT cells, memory B cells, and specific lipids (e.g., apolipoprotein A-I [ApoA-I]) in CAD. However, these immune subsets do not operate in a vacuum but rather interact closely within a specific tissue microenvironment. EAT, a metabolically active tissue adjacent to the coronary artery without a fascial barrier, is increasingly being recognized as a crucial immunological microenvironment in CHD, where innate immune signals, adaptive immune responses, and lipid metabolism disorders converge to determine the disease course of the coronary artery.

#### 4.3.1. ApoA-I Dysfunction: Metabolic Triggers in the EAT Microenvironment

ApoA-I is the main structural protein of HDL, and its classical function is to mediate reverse cholesterol transport. The function of ApoA-I in the EAT microenvironment is particularly significant. Impaired function or decreased concentration of ApoA-I in patients with CAD impairs the cholesterol efflux capacity of EAT-resident macrophages, leading to local lipid accumulation and foam cell formation, and also affects the local immune microenvironment through the following mechanisms: (1) Functional ApoA-I has been shown to have direct anti-inflammatory properties, including inhibition of monocyte chemotaxis, dendritic cell (DC) maturation, and T-cell activation; its loss of function thus lifts the brake on local immune activation. (2) Increased local lipid oxidation caused by ApoA-I dysfunction, such as the accumulation of Ox-LDLs and OxPLs, produces a large number of OSEs, which are taken up and presented by local DCs as neoantigens and directly initiate adaptive immune responses. (3) ApoA-I also maintains local immune tolerance by binding to lipopolysaccharide and scavenging DAMPs released by injured cells. Its deletion leads to an elevated DAMP concentration within the EAT, which further activates the NLRP3 inflammasome and forms a positive feedback loop. Therefore, ApoA-I dysfunction can be regarded as a key “switch” for the transition from metabolic stress to immune activation in the EAT microenvironment.

#### 4.3.2. γδ T Cells: The Bridge Cells Between Innate and Adaptive Immunity in EAT

γδT cells are a unique subset of T cells with both innate and adaptive immune characteristics. Unlike classical αβT cells, γδT cells do not rely on peptide antigens presented by MHC molecules. Instead, γδT cells are able to directly recognize non-peptide ligands, including phosphoantigens, lipid metabolites, and stress-induced molecules. Of note, γδT cells are naturally enriched in adipose tissue and are also significantly distributed in EAT.

Metabolic disturbances in the EAT microenvironment, especially enhanced lipid stress due to ApoA-I dysfunction, can activate resident γδT cells through several pathways during the progression of CHD: (1) Lipid metabolic intermediates (e.g., isopentenyl pyrophosphate and mevalonate pathway metabolites) can be directly recognized by the Vγ9Vδ2 TCR on the surface of γδT cells, triggering rapid cytokine secretion. (2) Stress ligands, such as MICA/B and ULBPs, upregulated in adipocytes and macrophages under lipid stress, can be recognized by NKG2D receptors on the surface of γδT cells. Once activated, EAT-resident γδT cells rapidly produce large amounts of IL-17A and IFN-γ. These cytokines have dual functions: IL-17A promotes the local recruitment of neutrophils and monocytes, amplifying innate inflammation. Conversely, IFN-γ promotes local DC maturation and antigen presentation efficiency, and enhances the activation of αβT cells, especially Th1 cells. Therefore, γδT cells serve as key “bridge cells” between lipid metabolic stress (level 1) and classical adaptive immune activation (level 2) in the EAT microenvironment.

#### 4.3.3. Memory B-Cell Class Switching: A Humoral Immune Shift Driven by the EAT Cytokine Milieu

The EAT is the site of T-cell immune responses and provides a unique microenvironment for local B-cell activation and functional differentiation. B-cell infiltration has been confirmed in the EAT of patients with CHD, and its abundance is correlated with the degree of local inflammation. Under normal immune homeostasis, natural IgM antibodies against Ox-LDLs and OSEs, such as those of the E06/T15 lineage, perform important protective functions, including clearing apoptotic cell debris, neutralizing lipid toxicity, and inhibiting proinflammatory macrophage activation.

However, in response to sustained proinflammatory cytokine stimulation in the EAT microenvironment, memory B cells undergo class switching from a protective IgM to an IgG subclass. This process is driven by the following local EAT factors: (1) IL-21 secreted by T follicular helper cells (Tfh) is a key signal for B-cell class switching and affinity maturation; The infiltration of Tfh-like cells in the EAT of patients with CHD can promote local germinal center reaction. (2) BAFF (B-cell activating factor) and APRIL released by EAT stromal cells and activated macrophages provide key co-stimulatory signals for B-cell survival and class switching. (3) IFN-γ produced by γδT cells could directly promote the conversion of B cells to IgG1 and IgG3 subclasses.

IgG antibodies produced by class switching may play harmful roles in CHD: (1) IgG antibodies against Ox-LDLs form immune complexes with oxidized lipids in the plaque, activate macrophages through Fcγ receptors, promote the release of proinflammatory cytokines and the expression of matrix metalloproteinases (MMPs), and accelerate plaque instability. (2) Immune complexes can also activate the classical complement pathway to produce complement fragments such as C3a and C5a, which further recruit inflammatory cells. (3) After the replacement of IgM with IgG, the protective clearance function of IgM against OSEs was relatively insufficient, resulting in the accumulation of apoptotic debris and the expansion of the necrotic core.

#### 4.3.4. Bidirectional Paracrine Signaling Between EAT and the Coronary Artery: A Unique “Outside-In” Pathway

The key reason why the above immune response of EAT has special pathological significance for coronary atherosclerosis is the lack of a fascial barrier between EAT and the coronary artery. This implies that EAT-derived adipokines, cytokines, and immune cells can penetrate directly into the adventitia and the outer tunica media of the coronary artery, establishing a unique “outside-in” signaling pathway. This is complementary to the classic endothelium-driven “inside-out” inflammation. In particular:

(1) IL-17A, IFN-γ, and TNF-α released by γδT cells and macrophages activated in EAT diffuse to the adventitia and activate pro-inflammatory pathways in adventitial fibroblasts and smooth muscle cells to promote vessel wall remodeling.

(2) EAT-derived chemokines such as MCP-1 and RANTES can enter the coronary artery wall through the vasa vasorum and promote the recruitment of peripheral blood monocytes to the adventitial side of the plaque. This “outside-in” immune cell infiltration pathway has been confirmed in human coronary artery specimens.

(3) IgG antibodies produced by class-switched memory B cells in the EAT can enter the adjacent plaque microenvironment through local diffusion, form immune complexes in situ in the plaque, and activate the complement cascade.

Multimodal imaging combining coronary CT angiography (CCTA) and optical coherence tomography has demonstrated a significant association between the attenuation of pericoronary adipose tissue (an imaging marker of EAT inflammation), the extent of macrophage infiltration, and adverse clinical outcomes. The hypothesis of EAT microenvironment–coronary artery crosstalk was further supported at the clinical imaging level.

#### 4.3.5. Integrative Perspective: EAT as a Potential Target for Immunotherapy of CHD

Taken together, the EAT microenvironment provides a typical scenario to materialize the three-level framework of this review: ApoA-I dysfunction and lipid stress (tier 1) activate EAT-resident γδT cells and macrophages (tier 1–2 bridge), which in turn drive αβT-cell polarization and memory B-cell class switching through DC antigen presentation and the cytokine environment (tier 2). All these immune effects ultimately converge on the coronary plaque (third level) through “outside-in” paracrine signals, which affect plaque stability and clinical outcomes.

This integrated perspective has several significant translational implications. First, the noninvasive assessment of EAT volume and inflammation (pericoronary fat attenuation index measured by CCTA) is a promising novel biomarker for the state of the local immune microenvironment. Secondly, therapeutic strategies targeting the EAT immune microenvironment include restoring ApoA-I function (e.g., rHDL infusion therapy), regulating γδT-cell activity (e.g., antibodies or small molecules targeting the Vγ9Vδ2 TCR), and inhibiting local B-cell class switching (e.g., targeting the BAFF/APRIL signaling axis), which may represent a new direction for precision immune regulation beyond systemic anti-inflammatory strategies. However, current direct evidence for the interaction of immune cell subsets within EAT is mainly derived from bioinformatics predictions and limited histological observations, and there is a lack of systematic validation data using spatial transcriptomics or multiplex immunofluorescence in human EAT specimens. Future studies should prioritize high-resolution immune mapping of the EAT–coronary interface in patients with CAD using single-cell spatial omics technologies such as Visium, MERFISH, or CODEX to validate the above immune cell crosstalk model in the spatial dimension.

### 4.4. The Paradigm Shift: From Linear Inflammation to Multidimensional Immune Dysregulation

Traditional models of atherosclerosis have emphasized a linear inflammatory cascade, proceeding from lipid deposition through monocyte recruitment, foam cell formation, and plaque rupture. The evidence reviewed here necessitates a paradigm shift toward a multidimensional model of immune dysregulation that incorporates several additional layers of complexity.

Phenotypic plasticity and functional switching are now recognized as fundamental properties of immune cells in CHD. Macrophages exhibit M1/M2 polarization plasticity (Section 2.3.1) [36,112], NK cells undergo compartment-dependent functional switching between circulation and plaque tissue (Section 2.6.3) [163], and Tregs can be reprogrammed toward a mixed Th1/Th17/Treg phenotype in dyslipidemic individuals (Section 3.2.3) [230]. These observations indicate that the functional state of immune cells, rather than their mere presence or absence, determines disease trajectory. Metabolic–immune coupling has emerged as a critical regulatory layer. Expression of the GLP-1 receptor on Tregs correlates with coronary stenosis severity (Section 3.2.3) [229], the glycolytic enzyme Pfkfb3 exhibits gene dosage effects on monocyte inflammatory output (Section 2.2.5) [118], and the ox-LDL–mast cell–histamine axis links lipid metabolism directly to immune activation (Section 2.8.4) [193]. These findings position cellular metabolism as a master regulator of immune cell function in CHD, and suggest that metabolic drugs, such as GLP-1 receptor agonists, may exert cardiovascular benefits partly through immune modulation. The concept of biphasic immune dysfunction, in which the same cell type simultaneously exhibits local pro-inflammatory activity and systemic immunosuppression, has been demonstrated for T cells through the CD155 immune checkpoint (Section 3.2.6) [243], and is further suggested for NK cells by their peripheral depletion despite concurrent local plaque accumulation (Section 2.6.5) [167,168]. This paradoxical phenotype challenges simplistic pro-inflammatory or anti-inflammatory classifications and has direct implications for therapeutic design: broadly immunosuppressive strategies may worsen systemic immune deficiency, while targeted local interventions may be more appropriate.

The most compelling human evidence supporting the principle of pathway-specific rather than pan-immunosuppressive intervention comes from the juxtaposition of several landmark randomized controlled trials. The CANTOS trial (Canakinumab Anti-inflammatory Thrombosis Outcomes Study) demonstrated that targeted neutralization of IL-1β with canakinumab significantly reduced recurrent major adverse cardiovascular events (MACE) in post-myocardial infarction patients with residual inflammatory risk (hsCRP ≥ 2 mg/L), independent of any lipid-lowering effect, providing the first proof of concept that pharmacological inhibition of a single node in the innate immune inflammatory cascade—specifically the NLRP3 inflammasome—IL-1β–IL-6–CRP axis can reduce atherosclerotic cardiovascular events in humans [263]. Notably, this IL-1β/IL-6 axis is the same pathway hyperactivated by CHIP-associated TET2-deficient macrophages, providing a mechanistic bridge from clonal hematopoiesis through macrophage inflammatory reprogramming to a validated therapeutic target. Two subsequent trials of low-dose colchicine reinforced this paradigm. COLCOT demonstrated a significant reduction in ischemic cardiovascular events when colchicine was initiated within 30 days after myocardial infarction [264], while LoDoCo2 showed similar benefits in patients with chronic coronary disease [265]. The mechanism of action of colchicine involves the inhibition of the NLRP3 inflammasome, suppression of neutrophil chemotaxis, and microtubule-dependent inflammatory signaling, thereby engaging the same innate immune pathway targeted by canakinumab, but through a distinct pharmacological mechanism.

Critically, the Cardiovascular Inflammation Reduction Trial (CIRT) provided an essential negative control: low-dose methotrexate—a broadly acting immunosuppressant—failed to reduce cardiovascular events in stable patients with atherosclerosis and did not lower IL-1β, IL-6, or CRP levels [266]. The stark contrast between the positive CANTOS/COLCOT/LoDoCo2 results and the negative CIRT outcome empirically validates the concept articulated above: cardiovascular benefit is conferred by pathway-specific targeting of the innate immune inflammatory cascade (particularly the NLRP3/IL-1β/IL-6 axis) rather than by nonspecific immunosuppression, which may suppress beneficial immune surveillance without engaging the pathogenic inflammatory pathway. This principle is further supported by emerging IL-6-targeted approaches: the RESCUE trial demonstrated that ziltivekimab, an anti-IL-6 ligand antibody, substantially reduced biomarkers of inflammation and thrombosis in patients with CKD and elevated hsCRP levels [267]. Studies are currently underway to determine whether IL-6 inhibition reduces MACE.

Taken together, these landmark trials provide the essential translational framework for interpreting the immune cell–CHD associations reviewed in the preceding sections: while numerous innate and adaptive immune cell subsets are implicated in atherogenesis and plaque instability, therapeutic benefit has thus far been demonstrated only through selective engagement of the NLRP3 inflammasome/IL-1β/IL-6 pathway, underscoring the necessity of precision immune targeting in cardiovascular medicine. The biphasic immune dysfunction phenotype discussed above—simultaneous local inflammation and systemic immunosuppression—further explains why CIRT-type broad immunosuppression may be ineffective and potentially harmful, as it may exacerbate the systemic immune deficiency arm without adequately suppressing the local inflammatory arm.

### 4.5. Translational Advances: Biomarkers, Genetic Causality, and Therapeutic Targets

In the biomarker domain, composite inflammatory indices derived from routine blood counts, including MHR, neutrophil-to-lymphocyte ratio (NLR), NHR, NAR, SII, and MLR, have been independently associated with coronary stenosis severity, plaque vulnerability, post-PCI outcomes, and long-term mortality in multivariable-adjusted observational studies (Section 2.2.4 and Section 2.4.4) [31,48,55,87,144,268,269]. Their cost-effectiveness and accessibility render them particularly suitable for resource-limited settings. However, this study has several important limitations. First, the degree of confounder adjustment varied across studies, with inconsistent control for renal function, BMI, and concurrent medications. Second, indices incorporating HDL-C as a denominator (MHR and NHR) partly recapitulate the established lipid-associated risk, whereas indices that share cellular components (SII, NLR, and PLR) exhibit collinearity. Third, and most critically, for the majority of these ratios, formal incremental discrimination analyses—ΔC-statistic, NRI, and IDI—evaluating whether they improve risk prediction beyond established cardiovascular risk models (e.g., Framingham Risk Score, SCORE, and GRACE) have not been reported. The limited evidence available for NLR suggests only modest incremental discrimination when added to the GRACE score for ACS. Consequently, the current evidence supports these composite ratios as associative biomarkers of systemic immune-inflammatory status that may inform pathophysiological understanding rather than as validated additions to clinical risk prediction algorithms. Future studies should be specifically designed to evaluate the incremental predictive value in prospective cohorts with standardized confounder adjustment, head-to-head comparison against ratio components, and prespecified discrimination and reclassification endpoints. More sophisticated approaches that integrate these ratios with metabolic indices (TyG) and machine learning algorithms (XGBoost, LASSO, WGCNA) have reported improved diagnostic or prognostic accuracy for acute myocardial infarction and heart failure risk (Section 2.2.6 and Section 2.4.4), although external validation in independent cohorts remains necessary [42,121]. In the genetic causality domain, Mendelian randomization studies have established causal links between genetically predicted NLR and CHD risk (Section 2.4.2) [133], between basophil counts and CHD (Section Genetic Evidence and Clinical Phenotype Associations, OR = 0.92) [114], between HLA-DR^+^ NK cells and lipid-mediated vascular injury (Section 2.6.2) [162], between DC-associated immunophenotypes and cardiovascular disease susceptibility (Section 2.7.7) [189], and between CD4^+^ T-cell activation genes and cardiometabolic disease targets (Section 3.2.7) [252]. These MR findings elevate immune cell abnormalities from correlative observations to causal participants in CHD pathogenesis, providing a stronger rationale for immune-targeted interventions. However, several methodological caveats should be noted when interpreting these MR findings. Most of the cited MR analyses derived their genetic instrumental variables from a single, modestly powered, circulating immunophenotype GWAS comprising approximately 3700 subjects. To obtain a sufficient number of genetic instruments, several of these studies employed relaxed *p*-value thresholds (e.g., *p* < 1 × 10^−5^) rather than the conventional genome-wide significance threshold (*p* < 5 × 10^−8^), which increases the risk of weak instrument bias, potential pleiotropic confounding, and violations of the exclusion restriction assumption. Furthermore, the GWAS from which these instruments were derived measured peripheral blood immune cell counts, which may not accurately reflect the immune cell composition, activation state, or functional phenotype within atherosclerotic plaques or infarcted myocardium. The biological correspondence between circulating immunophenotypes and tissue-resident immune infiltrates remains largely uncharacterized, and a genetically determined increase in the circulating cell count does not necessarily imply a proportional increase in the infiltration or activity of the cell type within the plaque microenvironment. Therefore, while MR provides evidence for potential causal relationships that are more robust than observational associations alone, the specific MR findings cited herein should be regarded as hypothesis-generating rather than definitive and require validation through larger-scale multi-ancestry GWAS, simultaneous tissue-level immunophenotyping, and, ultimately, pathway-specific interventional studies, such as those discussed in Section 4. In the therapeutic domain, several promising approaches have emerged. Nanomedicine platforms delivering dual-gas payloads to the plaque microenvironment can modulate macrophage polarization states with spatial precision (Section 2.3.6) [112]. The PCSK9 inhibitor evolocumab is being evaluated for its capacity to reduce macrophage burden in non-culprit lesions (Section 2.3.6) [39]. Dimethyl fumarate inhibits DC maturation and migration, offering a proof of concept for DC-targeted immunotherapy (Section 2.7.8) [190]. CCR5 inhibition with DAPTA restores Treg function in dyslipidemic individuals (Section 3.2.3) [230]. Traditional Chinese medicine formulations, such as Compound Danshen Dripping Pills, suppress monocyte-mediated inflammatory pathways (Section 2.2.7) [123]. These diverse therapeutic modalities, spanning nanomedicine, biologics, small molecules, and traditional medicine, reflect the breadth of actionable immune targets now available in CHD.

### 4.6. From Laboratory to Clinic: Translational Challenges in Cardiovascular Immunology

This review synthesizes immunological evidence from animal models and human clinical studies; however, it must be acknowledged that there is a significant translational gap between these two types of evidence. Failure to recognize this gap may lead not only to overinterpretation of experimental results but also to misdirection of therapeutic strategy development.

#### 4.6.1. Species-Specific Immunological Differences: An Underestimated Barrier to Translation

There are multifaceted differences in the underlying makeup of the immune system between rodents and humans, which directly affect the extrapolability of immunological findings related to CHD.

(1) Fundamental differences in circulating leukocyte lineages. The peripheral blood of mice and rats is dominated by lymphocytes (approximately 75–90%), whereas that of humans is dominated by neutrophils (approximately 50–70%). This fundamental difference means that the baseline values and dynamic changes in composite biomarkers based on blood routine—such as the NLR and SII discussed in this review—in rodents are markedly different from those in humans and cannot be simply compared across species.

(2) Pathological differences in atherosclerotic plaques: Standard ApoE^−/−^ and Ldlr^−/−^ mouse models successfully recapitulate features of chronic atherosclerosis, such as early lipid deposition, foam cell formation, and fibrous cap formation, but rarely develop spontaneous plaque rupture and subsequent thrombosis, the core pathological event in human ACS. Thus, the immune cell infiltration patterns and functional status observed in these models may only be applicable to understanding chronic plaque progression and cannot be directly extrapolated to the immune mechanisms of plaque rupture and ACS in humans. Notably, the recently developed tandem stenosis model and certain genetically engineered mice, such as ApoE^−/−^Fbn1C1039G^+/−^ mice, can induce plaque rupture-like events, but the human relevance of their immunological features still needs further validation.

(3) Species differences in mast cell distribution. As described in Section 2.8.6, the shoulder region of human atherosclerotic plaques is rich in mast cells, whereas mouse plaques are sparsely populated with mast cells. This difference may explain the inconsistent results of previous studies regarding the effects of mast cells on atherosclerosis in mouse models.

(4) Differences in the proportion and function of T-cell subsets. The ratio of CD4/CD8 T cells in the spleen and peripheral blood, the definition of memory T-cell surface markers, and the functional characteristics of Tregs in mice differ from those in humans. For example, Foxp3, a classical marker of mouse Tregs, is transiently expressed in activated effector T cells in humans, introducing potential identification bias when cross-species comparisons rely solely on Foxp3 expression.

(5) The relationship between the myocardial injury model and human CHD. The LAD ligation and isoproterenol-induced injury models recapitulate the immune repair process after acute myocardial ischemia/infarction, rather than progressive myocardial ischemia caused by chronic atherosclerosis. The eosinophil-derived IL-4 secretion, basophil-mediated repair of fibrosis, and regulation of myocardial remodeling by Tregs observed in these models (see Section Dual and Conflicting Roles in Myocardial Infarction, Basophil-Driven Reparative Fibrosis and Section 4.4, respectively) are wound-healing responses rather than atherosclerotic plaque immune responses in nature. The corresponding disease stage must be specified when interpreting and citing these data.

#### 4.6.2. Translational Cases of Success and Failure: Pathway Specificity Is Crucial

The field of cardiovascular immunology has accumulated important translational experiences with both successes and failures that provide valuable lessons for future translation of the immune targets discussed in this review.

Success case—IL-1β pathway and CANTOS experiment: A large number of animal models have confirmed the critical pro-inflammatory role of the NLRP3–IL-1β axis in the progression of atherosclerosis. This fundamental finding ultimately led to the landmark CANTOS trial, in which the use of the specific anti-Il-1β monoclonal antibody canakinumab significantly reduced MACE in 10,061 patients with prior myocardial infarction and elevated hsCRP. Targeting specific inflammatory pathways was demonstrated for the first time in humans to improve cardiovascular outcomes independent of lipid-lowering therapy. The success of CANTOS illustrates that the success rate of translation is significantly increased when targets in animal models have a clear mechanistic chain and when human genetic evidence, such as the association of IL-1 cluster polymorphisms with the risk of CHD, provides independent validation.

Success stories—NLRP3/microtubule pathway and colchicine: The COLCOT and LoDoCo2 trials confirmed that low-dose colchicine, partly through the inhibition of NLRP3 inflammasome assembly and neutrophil chemotaxis, significantly reduces the risk of cardiovascular events, further supporting the value of targeting innate immune-inflammatory pathways in the secondary prevention of CHD.

Failure case—Broad-spectrum immunosuppression vs. the CIRT trial: In contrast, the CIRT trial, which used the broad-spectrum immunosuppressant methotrexate, which primarily inhibits purine synthesis and folate metabolism, did not reduce the risk of cardiovascular events or significantly reduce levels of IL-1β, IL-6, or CRP. This negative result clearly indicates that the benefit of immunotherapy in CAD results from the highly specific inhibition of inflammatory pathways rather than from broad systemic immunosuppression, which has important implications for the pathways to translation of all the potential immune targets discussed in this review.

Cases of failure—anti-TNF-α therapy and heart failure: Anti-Tnf-α biological agents (infliximab and etanercept), which are highly effective in rheumatoid arthritis, showed no benefit or even worsened outcomes in clinical trials of heart failure, suggesting that the same immune pathway may play distinct roles in different cardiovascular disease contexts, further emphasizing the need for mechanistic research and careful translation.

#### 4.6.3. Emerging Technologies and Strategies to Bridge the Translational Gap

Several technologies and strategies have been developed to overcome these barriers.

(1) Humanized mouse models. Transplantation of human hematopoietic stem cells or peripheral blood mononuclear cells into immunodeficient mice (e.g., NSG or NOG strains) can partially reconstitute the human immune system. The combination of humanized immune reconstitution with an atherosclerosis-prone genetic background, such as a human ApoB100 transgene, holds promise for creating CHD models that more closely resemble human immune-metabolic interactions.

(2) Organ-on-a-chip technology. Microfluidic chips can be used to reconstruct atherosclerotic microenvironments containing human endothelial cells, smooth muscle cells, monocytes/macrophages, and physiological blood-flow shear stress in vitro. Atherosclerosis-on-a-chip systems could be used to test the effects of immunomodulatory strategies while avoiding interference by species differences.

(3) Cross-species multi-omics validation. Cross-species comparison of single-cell RNA sequencing data from mouse models with data from human patients with CHD can help identify evolutionarily conserved immune cell states and regulatory networks, thereby prioritizing the clinical development of immune targets that show congruence in both species.

(4) The strategic deployment of large animal models. The coronary anatomy, hemodynamics, and atherosclerotic plaque morphology in pigs are highly similar to those in humans. Immune targets that have shown therapeutic potential in mouse models should be validated in porcine models of atherosclerosis, such as high-fat-fed Yucatan minipigs or familial hypercholesterolemia pigs, before progression to human clinical trials.

(5) Parallel development of biomarkers. The clinical translation of each potential immunotherapy target should be accompanied by the development of companion biomarkers that can be used for patient stratification. For example, if a particular T-cell subset is targeted, flow cytometric panels or serologic markers that can detect the frequency or functional status of that subset in the peripheral blood should be developed to identify the patient population most likely to benefit in clinical trials.

### 4.7. Herbal Medicines and Innate Immunomodulatory Compounds in CHD: Bridging Traditional Medicine with Evidence-Based Cardiology

This review cites preclinical evidence of several herbal medicines and natural compounds (e.g., Xin-Li formula and Acorus tatarinowii rhizome glucan) that modulate the immune microenvironment of CHD. In addition, metabolic risk factors such as hyperlipidemia and hyperhomocysteinemia are closely related to immune activation, and TCM prescriptions are often used as adjunctive treatments for these metabolic disorders. However, a substantial gap exists between preclinical immunomodulatory effects and their use in clinical practice. Here, we provide an objective and careful evaluation of these natural therapies in terms of three key dimensions: safety, standardization, and bioavailability.

#### 4.7.1. Safety Assessment: Non-Negligible Toxicological Considerations

Although many natural compounds have shown anti-inflammatory and immunomodulatory activities in vitro and in animal models, their safety—particularly in patients with coronary heart disease, a vulnerable population that often has multiple coexisting chronic conditions and is receiving multiple medications—should not be taken for granted. The following issues require special attention:

(1) Risk of hepatotoxicity. Drug-induced liver injury (DILI) has been reported for a variety of Chinese herbal medicine components, such as plants containing pyrrolizidine alkaloids and Polygonum multiflorum. Statins, which are often co-administered in patients with coronary heart disease, undergo hepatic metabolism. The additive effect of herbal ingredients and statins on liver metabolism may increase the risk of hepatotoxicity.

(2) Risk of bleeding. Patients with coronary artery disease almost universally receive antiplatelet therapy (aspirin, clopidogrel/ticagrelor), and some also receive anticoagulant therapy. Several natural compounds (e.g., tanshinones, Ginkgo biloba extract) are known to have anticoagulant or antiplatelet activity, and their use in combination with standard antithrombotic regimens may result in additive or synergistic increases in bleeding risk.

(3) The double-edged sword of immunomodulation. Long-term safety data are lacking regarding whether the immunomodulatory activity of natural compounds, while reducing atherosclerotic inflammation, may inhibit normal immune surveillance, such as tumor immune surveillance or anti-infective immunity.

(4) Lack of systematic toxicology studies. Most herbal formulations have only been evaluated for acute toxicity in short-term animal experiments, and there is a lack of complete toxicology data packages that meet international drug regulatory standards (ICH S1–S9 guidelines, https://database.ich.org/sites/default/files/S9_Guideline.pdf, accessed on 25 September 2026), including chronic toxicity, genotoxicity, reproductive toxicity, and carcinogenicity studies.

#### 4.7.2. Standardization: The Core Bottleneck from “Metaphysics” to “Science”

Standardization is the fundamental bottleneck for herbal and natural compounds to move from traditional experience to evidence-based medicine:

(1) Multi-component complexity. Unlike chemically synthesized single-molecule drugs, herbal formulations such as cardiac herbal formulas often contain dozens or even hundreds of known and unknown chemical components. The proportion and content of these components are strongly influenced by the geographical origin, growing season, harvesting time, processing technology, and storage conditions of the raw herbal materials. This inherent chemical heterogeneity means that products from different batches, different manufacturers, or even different batches of the same manufacturer may have significantly different chemical compositions and biological activities.

(2) Quality control methodology. At present, the commonly used methods for quality control of traditional Chinese medicine (TCM), such as thin-layer chromatography (TLC) identification and quantification of a single marker compound, are far from sufficient to ensure batch-to-batch consistency between batches. More advanced fingerprinting techniques, including HPLC, UPLC-MS/MS, and NMR chemical fingerprinting, combined with simultaneous quantitative analysis of multiple marker compounds, need to be introduced to establish a traceable quality standard system.

(3) Promotion of the concept of “effective component cluster.” From the perspective of immunoregulation, future research should focus on identifying the “effect component clusters” (such as specific polysaccharides, flavonoids, or alkaloids) that truly play an immunomodulatory role in herbal formulations and use these clusters as a standardized target for quality control, rather than relying on crude standardization of the whole prescription. For rhizome glucans from Acorus tatarinowii, the molecular weight distribution, linkage type, and branched chain structure of glucans should be clarified, because these structural characteristics directly determine their binding affinity to pattern recognition receptors (such as Dectin-1 and TLR-2/4) on the surface of immune cells and the direction of immune regulation.

#### 4.7.3. Bioavailability: The Pharmacokinetic Gap from Drug Administration to Target Tissue

Even if safety and standardization issues are addressed, many natural compounds face serious bioavailability challenges:

(1) Oral malabsorption. Most polyphenols, flavonoids, and macromolecular polysaccharides have very low oral bioavailability (typically < 5%) because of intestinal pH instability, poor gut wall permeability (high molecular weight, too hydrophilic, or too hydrophobic), gut enzymatic degradation, and metabolism by the gut microbiota.

(2) First-pass effect. Natural compounds absorbed through the portal system undergo extensive hepatic first-pass metabolism (phase I and phase II reactions) before reaching the systemic circulation, further reducing systemic exposure.

(3) Unknown distribution in target tissues. Limited data are available on whether natural compounds can reach target tissues, such as atherosclerotic plaques or epicardial adipose tissue, at therapeutically relevant concentrations, even when measurable drug concentrations are detected in plasma. In the absence of tissue distribution data, the micromolar-level concentrations used in the in vitro experiments may be much higher than the actual exposure of target tissues in vivo, leading to overestimation of potency.

(4) Novel delivery strategies. To overcome these bottlenecks, nanotechnology platforms are being introduced into the delivery of natural compounds. Delivery systems such as liposome encapsulation, poly (lactic-co-glycolic acid) (PLGA), chitosan nanocarriers, and exosome-like vesicles are expected to improve the targeting and bioavailability of natural compounds. For example, macrophage-targeting nanoparticles can specifically deliver anti-inflammatory natural compounds to macrophages within a plaque, resulting in local release at high concentrations. However, these delivery strategies themselves are in the early stages of development, and their clinical safety and accessibility need to be validated.

#### 4.7.4. Drug–Herb Interactions: Key Issues in the Context of Polypharmacy for Coronary Heart Disease

The standard treatment for patients with coronary heart disease usually includes the combination of multiple drugs (statins, antiplatelet drugs, ACEI/ARBs, β-blockers), forming a complex multi-drug treatment environment. The addition of herbal or natural compounds in this context may trigger interactions at both pharmacokinetic (PK) and pharmacodynamic (PD) levels:

(1) Interference with the CYP450 enzyme system. Various herbal constituents are known to inhibit or induce key metabolic enzymes such as CYP3A4, CYP2C19, and CYP2D6. For example, inhibition of CYP3A4 results in markedly elevated plasma concentrations of simvastatin or atorvastatin, increasing the risk of rhabdomyolysis. The inhibition of CYP2C19 may impair the bioactivation of clopidogrel to its active metabolite and reduce its antiplatelet efficacy.

(2) Interference with P-glycoprotein (P-gp) and other transporters. Some natural compounds can modulate the activity of efflux transporters such as P-gp and affect the absorption and clearance of drug substrates (such as dabigatran and digoxin).

(3) Additive pharmacodynamic effects. As mentioned above, natural compounds with anti-inflammatory, antiplatelet, or lipid-lowering activity, when combined with standard drugs, may have unexpected additive or synergistic effects, increasing the risk of adverse effects.

#### 4.7.5. Future Research Directions and Methodology Recommendations

For herbal medicines and innate immunomodulatory compounds to gain evidence-based acceptance in the field of coronary heart disease, future research should follow the following paths:

(1) Strict clinical trial design. Well-designed randomized, double-blind, placebo-controlled clinical trials with major adverse cardiovascular events (MACE) as the primary endpoint should be conducted, rather than relying solely on surrogate endpoints such as reduction in serum inflammatory markers. Trial reporting should follow the CONSORT Statement and its extended version for Chinese Herbal Medicines (CONSORT-CHM) to ensure transparency and reproducibility.

(2) Systemic pharmacokinetic/pharmacodynamic (PK/PD) studies. These studies should systematically evaluate the PK/PD parameters of natural compounds to clarify the direction and magnitude of drug–herb interactions in the multi-drug context of standard treatment regimens for coronary heart disease.

(3) Isolation and verification of effective components. Isolation and identification of active monomers or effector components with clear immunomodulatory targets from compounds, standardization, and pharmacological evaluation of these well-defined chemical entities should be conducted, rather than studying exclusively at the level of the whole compound formulation.

(4) Exploration of regulatory pathways. Draw on the experience of the US FDA Botanical Drug Guidance and the European Medicines Agency (EMA) traditional herbal product registration pathway to develop a standardized development and registration process suitable for multi-component natural products.

In conclusion, the preclinical evidence of herbal and natural compounds in the immunomodulation of CHD is encouraging, but there is still a long road from basic research to clinical practice. In the absence of high-quality clinical trial data and complete pharmacological evaluation, clinical recommendations for these natural remedies should be made with caution. Combining the wisdom of traditional medicine with the methodology of modern evidence-based medicine represents the essential path forward for advancing this field.

### 4.8. Current Limitations

Despite these advances, several overarching limitations must be acknowledged. (1) Peripheral blood–plaque tissue disconnect: The majority of clinical evidence derives from peripheral blood samples, which may not accurately reflect the local immune microenvironment within coronary plaques. This limitation applies across virtually all cell types reviewed, from monocytes and neutrophils to T cells, B cells, and mast cells. Peripheral eosinopenia may coexist with local eosinophil accumulation (Section Dual and Conflicting Roles in Myocardial Infarction) [44], and circulating NK cell depletion may reflect tissue recruitment rather than true systemic deficiency (Section 2.6.5) [167,168]. Future studies must integrate coronary plaque tissue analysis with peripheral blood profiling to resolve these discrepancies. (2) Subset definition heterogeneity: Different studies employ variable marker panels to define monocyte subsets (classical/intermediate/non-classical) [29,270], macrophage polarization states (M1/M2) [69,223], T-cell subpopulations (Treg, Th17, γδ T) [64,65,253], B-cell subsets (B-1, B-2, Bregs, naïve, memory) [200,201,204], and DC subtypes (cDC1, cDC2, pDC) [171,172]. This lack of standardization complicates cross-study comparisons and severely hinders meta-analytic synthesis. (3) Uneven evidence hierarchy: While Mendelian randomization (MR) studies provide causal inference and multi-omics analyses offer mechanistic depth, many findings, particularly those related to mast cells (Section 2.8.7) [35,42], eosinophils [44,45], and basophils [154,156], still rely predominantly on bioinformatics analyses, case reports, and indirect evidence. Large-scale prospective clinical trials and functional validation experiments are needed to confirm the therapeutic relevance of these observations. (4) Interspecies differences: in immune cell distribution and function—most notably for mast cells (Section 2.8.6)—limit the direct translatability of murine model findings to human disease.

### 4.9. Future Directions

To address these limitations, several priority areas for future research must be pursued: (1) Spatiotemporal multi-omics: The application of spatial transcriptomics and single-cell multi-omics to coronary plaque tissue will enable the characterization of immune cell subsets in their native microenvironment, resolving the peripheral blood–tissue disconnect and revealing spatiotemporal dynamics of immune remodeling at different disease stages [188,271]. (2) Prognostic model construction: The development of multi-omics prognostic models integrating macrophage polarization status, efferocytosis function, T-cell checkpoint expression, B-cell subset distribution, and adipose tissue interaction data will provide more comprehensive risk stratification tools than current single-marker approaches [42,121]. (3) Longitudinal cohorts: Longitudinal clinical cohorts with serial immune profiling will be essential for distinguishing cause from consequence among immune cell alterations, complementing the causal inference provided by MR studies with temporal resolution [133,156,162,189,252]. (4) Precision immunotherapy trials: Targeting specific immune pathways, such as CCR5 inhibition for Treg restoration [230], DC maturation blockade with dimethyl fumarate [190], or macrophage-targeted nanomedicine [112], should be designed with immune biomarker-guided patient selection to maximize therapeutic efficacy and minimize off-target immunosuppression. Furthermore, translating immunological findings into comprehensive cardiac repair requires interdisciplinary integration. For instance, myocardial structural remodeling following infarction frequently gives rise to fatal arrhythmias. In recent years, emerging conductive biomaterials (such as PEDOT:PSS-based hydrogels with excellent electrical properties) have shown great potential in bridging damaged cardiac tissue and preventing arrhythmias [272,273]. Combining these advanced biomaterials with local immune microenvironment regulation strategies may represent a highly promising new direction for future cardiac repair and arrhythmia prevention after myocardial infarction in CHD.

The “comorbidity immunology” perspective (Section 3.2.7), which recognizes shared immune mechanisms between CHD and systemic inflammatory diseases such as psoriasis, diabetes, and obesity [252], should be expanded to identify common therapeutic targets that transcend individual disease boundaries.

## 5. Conclusions

Immune dysregulation is not merely an accompaniment of coronary heart disease but a fundamental driver of its initiation, progression, and acute complications [203]. Research over the past five years has transformed our understanding of immune cells in CHD from a monocyte/macrophage-centric, linear inflammatory model to a multidimensional network encompassing all major innate and adaptive immune cell types. Each cell type exhibits context-dependent functional plasticity [112,163,230], metabolic–immune coupling [118,193,229], and bidirectional interactions with other immune and non-immune cells within the vascular microenvironment [42,188].

The convergence of composite inflammatory biomarkers [31,48,87,144], Mendelian randomization-based causal evidence [133,156,162,189,252], single-cell multi-omics [188,271], and emerging immunotherapeutic strategies [112,190,230] has created an unprecedented opportunity for precision immune intervention in CHD. However, realizing this potential will require overcoming the current limitations of peripheral blood-based evidence [44,167], heterogeneous subset definitions [29,171], and insufficient functional validation [35,42]. By integrating spatial multi-omics with longitudinal clinical cohorts and mechanism-driven therapeutic trials, the field is poised to translate the rich immunological insights of the past decade into tangible clinical benefits for patients with coronary heart disease.

## Data Availability

No new data were created or analyzed in this study. Data sharing is not applicable to this article.
